# Combining the Elicitor Up-Regulated Production of Unusual Linear Diterpene-Derived Variants for an In-Depth Assessment of the Application Value and Risk of the Medicinal and Edible Basidiomycete *Schizophyllum commune*

**DOI:** 10.3390/molecules29112608

**Published:** 2024-06-01

**Authors:** Ying Wang, Fei Cao, Luning Zhou, Hanwei Liu, Hua Gao, Ge Cui, Changshan Niu, Peng Zhang, Dehai Li, Songqi Liu, Yan Jiang, Guangwei Wu

**Affiliations:** 1 College of Chemical Engineering, Nanjing Forestry University, Nanjing 210037, China; 15861022729@163.com (Y.W.); 13485229495@163.com (H.G.); gecui@njfu.edu.cn (G.C.); liusongqi30@163.com (S.L.); 2 Key Laboratory of Pharmaceutical Quality Control of Hebei Province, Key Laboratory of Medicinal Chemistry and Molecular Diagnostics of Education Ministry of China, College of Pharmaceutical Sciences, Hebei University, Baoding 071002, China; caofei542927001@163.com; 3 Key Laboratory of Marine Drugs, Chinese Ministry of Education, School of Medicine and Pharmacy, Ocean University of China, 5 Yushan Road, Qingdao 266003, China; 18895692529@163.com (L.Z.); dehaili@ouc.edu.cn (D.L.); 4 Ningbo Customs District Technology Center, Ningbo 315100, China; 13646621936@163.com; 5 Department of Pharmacology and Toxicology, College of Pharmacy, University of Utah, Salt Lake City, UT 84112, USA; niucs88@gmail.com (C.N.); zhangpeng_cnu@163.com (P.Z.)

**Keywords:** *Schizophyllum commune*, combination of elicitors, diterpenes, antifungal activity

## Abstract

To better assess the practical value and avoid potential risks of the traditionally medicinal and edible basidiomycete *Schizophyllum commune*, which may arise from undescribed metabolites, a combination of elicitors was introduced for the first time to discover products from cryptic and low-expressed gene clusters under laboratory cultivation. Treating *S. commune* NJFU21 with the combination of five elicitors led to the upregulated production of a class of unusual linear diterpene-derived variants, including eleven new ones (**1**–**11**), along with three known ones (**12**–**14**). The structures and stereochemistry were determined by 1D and 2D NMR, HRESIMS, ECD, OR and VCD calculations. Notably, the elongation terminus of all the diterpenes was decorated by an unusual butenedioic acid moiety. Compound **1** was a rare monocyclic diterpene, while **2**–**6** possessed a tetrahydrofuran moiety. The truncated metabolites **4**, **5** and **13** belong to the trinorditerpenes. All the diterpenes displayed approximately 70% scavenging of hydroxyl radicals at 50 μM and null cytotoxic activity at 10 μM. In addition, compound **1** exhibited potent antifungal activity against the plant pathogenic fungi *Colletotrichum camelliae*, with MIC values of 8 μg/mL. Our findings indicated that this class of diterpenes could provide valuable protectants for cosmetic ingredients and the lead compounds for agricultural fungicide development.

## 1. Introduction

The basidiomycete *Schizophyllum commune* has generated considerable attention as a kind of valuable medicinal and edible mushroom [1,2,3]. The species *S. commune* is well-known for being rich in highly nutritious supplements, such as a wealth of amino acids and essential trace elements for the human body, high-quality protein, etc., and thus it is unusually regarded as a delicious dish [4]. *S. commune* has also shown great potential for drug development, particularly in the area of macromolecular exopolysaccharide substances [1]. A notable example is schizophyllan, an exopolysaccharide used as a biological-response modifier in combination with chemo and radiation therapy [4].

In 2023, the crude extract of *S. commune* was approved as a cosmetic ingredient for use as a skin moisturizer and protectant [5], wherein the exopolysaccharide plays a crucial role, by the China National Regulatory Commission. Unlike macromolecules in higher fungi, the potential applications of small molecules remain a mystery [6,7,8]. Currently, according to available bioinformatics data, this extremophilic fungus is known to encode at least 19 biosynthetic gene clusters, including those related to terpenes, non-ribosomal peptide synthetases (NRPSs), etc. [9]. To date, only four classes of small molecules, including terpenoids [10], alkaloids [4,11], aromatics [12] and polyketides [13], have been identified. It is evident that most chemotypes of small molecules, encoded by gene clusters that are cryptic and low-expressed, have not yet been characterized. Given the multiple values in drug development, and the uses of this organism as an edible mushroom and a raw material for cosmetics, it is necessary to explore the yet-undiscovered small molecules. It is also important to comprehensively assess the in-depth potentials and risks associated with the safety and new applications of higher fungi.

A variety of methods have been applied to activate natural products, including One Strain Many Compounds (OSMACs) [14,15], co-culture [16], chemical epigenetic regulation [17,18] and heterologous expression [19] methods, and so on. The High-Throughput Elicitors Screening (HITES) strategy is another highly effective approach for activating silent gene clusters. It offers wide applicability and represents a cutting-edge method for activating microbial secondary metabolites. Initially applied primarily to actinomycetes and bacteria, the use of HITES in fungi was first reported by Professor Mohammod in 2022 [20]. During the elicitor screening process, single-factor experiments are typically employed, which tend to be time-consuming and not conducive to high-throughput screening. The utilization of combined elicitors might offer a higher probability of activating new substances. This approach also has the potential to enhance efficiency when screening a large number of microorganisms. However, the application of a combinatorial elicitor strategy in microbial screening has not yet been reported.

**Figure 1:**
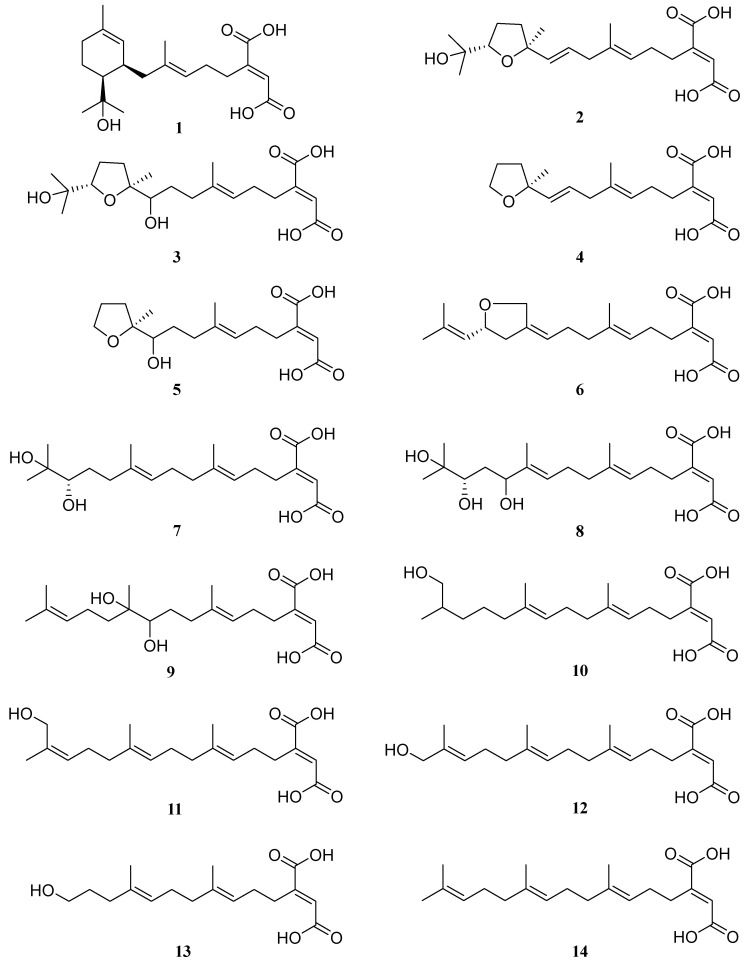
The structures of compounds **1**–**14**.

Herein, we developed a combination of elicitors to significantly enhance the likelihood of discovering previously undescribed small molecules in fungi. This approach was applied to activate and up-regulate the production of small molecules in *S. commune* NJFU21. As a result, a series of linear diterpene-derived variants **1**–**14** (Figure 1), were successfully produced in higher quantities. These small molecules enable us to more thoroughly investigate the practical value and assess the potential risks associated with the small molecules from *S. commune*, as well as their biological functions.

## 2. Results and Discussion

### 2.1. Selection and Screening

Previous studies have provided sufficient evidence that a single elicitor could activate the expression of metabolites of a cryptic or extremely low-expressed gene cluster. Thus, it is logically hypothesized that elicitors regulating gene expression in various manners could synergistically affect each other; the combination strategy of elicitors was able to activate and up-regulate the metabolite production as much as possible, once.

**Figure 2:**
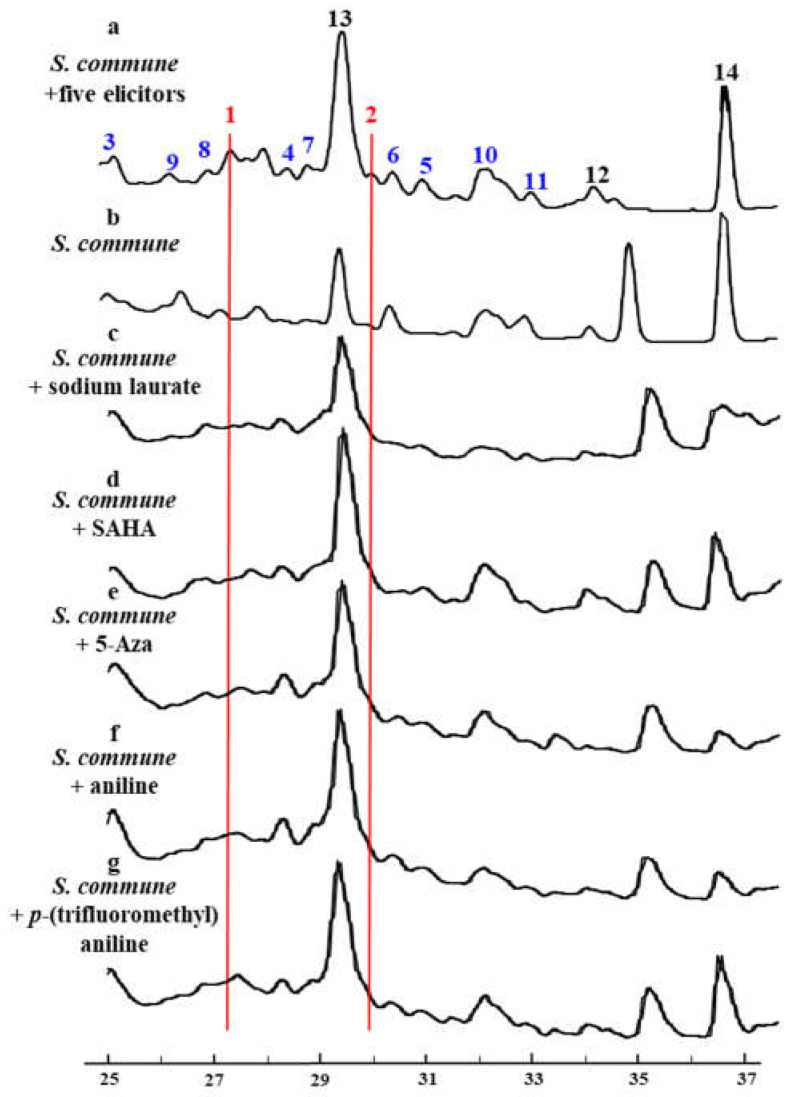
Changes in secondary metabolite profiles of *S. commune* NJFU21 analyzed by HPLC. a: Five elicitors were concurrently introduced into *S. commune* NJFU21 strain. b–g: Representing the individual *S. commune* NJFU21 and the elicitors added independently, respectively.

To verify the hypothesis, five function-verified elicitors–Sodium Laurate, Aniline, *p*-Trifluoromethylaniline, Vorinostat (SAHA) and 5-Azacytidine (5-Aza) were selected and eight concentration gradients were set to acquire the suitable work concentration of each elicitor and the universal applicability for most fungi. The mycelium growth and changes in secondary metabolite profiles were considered as indication signs. The change in secondary metabolite profile was analyzed by HPLC. As results, 0.1 mM of sodium laurate, aniline, and *p*-trifluoromethylaniline, respectively, and 0.075 mM of SAHA and 0.05 mM of 5-Azacytidine were determined as the suitable work concentration at which each elicitor does not affect the growth of fungi. When the combination of the five elicitors with individual concentrations was added into the culture of *S*. *commune* NJFU21, a series of new peaks with similar UV absorption appeared by HPLC analysis, indicating a class of secondary metabolites which was unregulated (Figure 2).

### 2.2. Structural Elucidation

Schizostatin B (**1**) was obtained as white powder. The molecular formula was determined as C_20_H_30_O_5_ based on the HR-ESI-MS ions at *m*/*z*, 351.2174 [M + H]^+^ (calcd for C_20_H_31_O_5_, 351.2166), indicating six degrees of unsaturation. Absorption bands at 3405 and 1647 cm^–1^ in the IR spectrum suggested the existence of hydroxy and carboxylic acid groups. Careful inspection of 1D NMR spectra (Table 1 and Table 2) suggested **1** possessed four methyls, five sp^3^ methylenes, five methines (two sp^3^ ones and three olefinic ones), six non-protonated carbons, including an oxygenated tertiary carbon at 73.4 (C-15), three olefinic carbons at 148.6 (C-3), 135.9 (C-7), 133.6 (C-11), and two carbonyl carbons at 170.1 (C-1), 169.0 (C-20), indicative of the fact that **1** is a monocyclic product.

**Figure 3:**
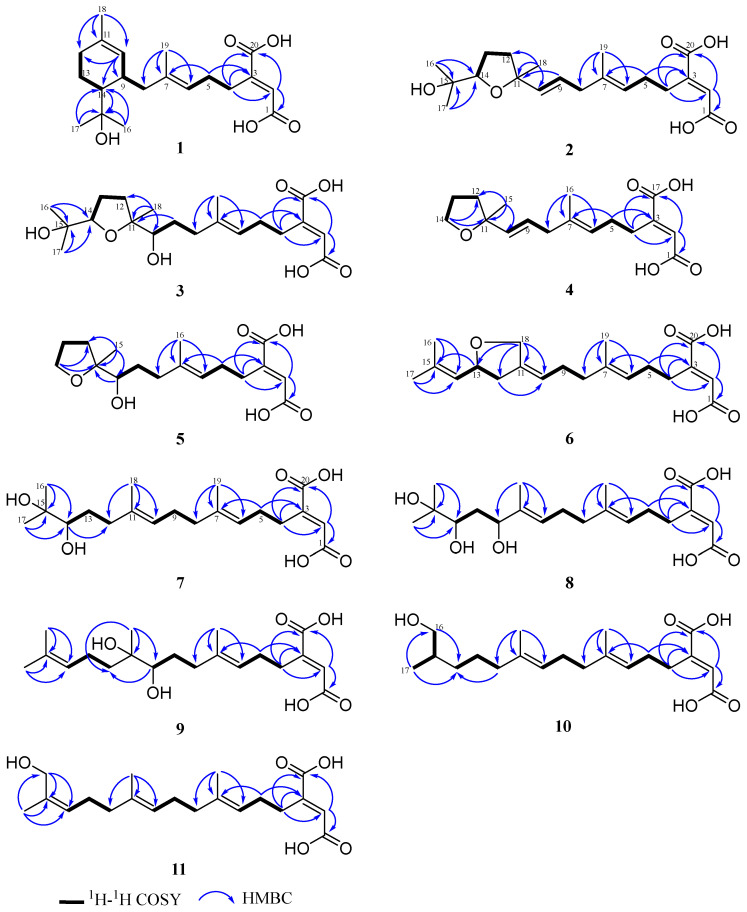
Key ^1^H-^1^H COSY, HMBC correlations of compounds **1**–**11**.

Based on the ^1^H-^1^H COSY spectrum, three spin systems of H_2_-4/H_2_-5/H-6, H-10/H-9/H_2_-8, H-14/H_2_-13/H_2_-12 were observed. The key HMBC correlations for H_3_-19 (*δ*_C/H_ 16.4/1.63) to C-6 (*δ*_C_ 125.6), C-7 (*δ*_C_ 135.9) and C-8 (*δ*_C_ 42.5) unambiguously established the connection between the fragment of H_2_-4/H_2_-5/H-6 and the fragment of H_2_-8/H-9/H-10 by a double-bond carbon of C-7 (Figure 3). Subsequently, a trisubstituted cyclohexene was established by the HMBC correlations for H_3_-18 (*δ*_C/H_ 23.7/1.62) to C-10 (*δ*_C_ 127.4), C-11 (*δ*_C_ 133.6) and C-12 (*δ*_C_ 32.9), in which the connectivity of C-10 and C-12 was determined through a double-bond carbon of C-11. The *gem*-dimethyl group was positioned to the oxygenated tertiary carbon C-15 (*δ*_C_ 73.4), and the latter carbon was attached to the C-14 of cyclohexene, evidenced by the HMBC correlations from H_3_-16 (*δ*_C/H_ 29.2/1.25)/17 (*δ*_C/H_ 27.7/1.23) to C-14 (*δ*_C_ 49.5), C-15 (*δ*_C_ 73.4) and H-14 (*δ*_C/H_ 49.5/1.55) to C-15 (*δ*_C_ 73.4). On the other end, the presence of the butenedioic acid moiety was confirmed by a combination of unsaturated-degree consideration and the key HMBC correlations of H-2 (*δ*_C/H_ 128.4/6.72) with C-1 (*δ*_C_ 170.1) and C-20 (*δ*_C_ 169.0), which was further attached to C-4, supported by the HMBC correlations from H-4 (*δ*_C/H_ 28.7/2.79) to C-3 (*δ*_C_ 148.6), C-2 (*δ*_C_ 128.3), C-1 (*δ*_C_ 170.1), as well as from H_2_-5 (*δ*_C/H_ 28.6/2.19) to C-3 (*δ*_C_ 148.6) (Figure 3). Accordingly, the planar structure of **1** was a monocyclic diterpene with an unusual butenedioic acid moiety.

**Figure 4:**
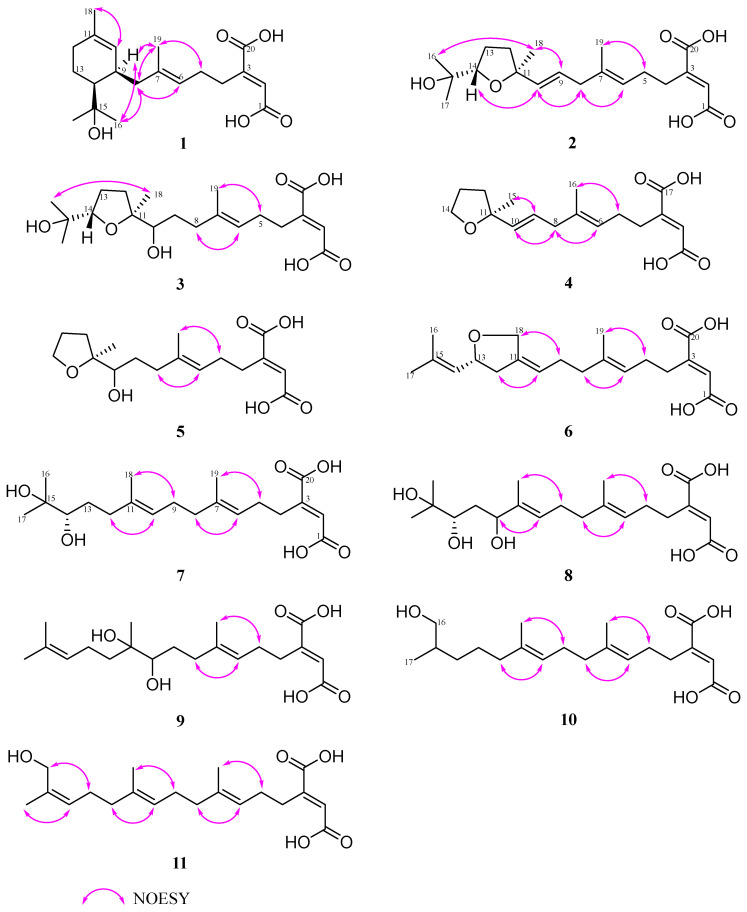
Key NOESY correlations of compounds **1**–**11**.

The relative stereochemistry of **1** was determined by the NOESY spectrum. The vital correlations from H-6 to H_2_-8 and from H_3_-19 to H_2_-5 indicated the *trans* configuration of the double bond at C-6 (Figure 4). However, the NOESY correlations of H_2_-8 with H_3_-16, and H-9 (*δ*_H_ 2.43) with H_3_-16 (*δ*_H_ 1.25) were observed, which failed to assign the relative configuration between C-9 and C-14 in the cyclohexene of **1**. To establish the relative configurations of C-9 and C-14, ^13^C NMR calculations were performed at the B3LYP/6-311+G (d, p) level. As a result, (9R*, 14S*)−**1** shows a 100% probability in contrast to (9S*, 14S*)−**1** (Figures S9 and S10).

Schizostatin C (**2**) was obtained as white powder, and the molecular formula was deduced as C_20_H_30_O_6_ from HR-ESI-MS ion peak at *m*/*z* 367.2108 [M + H]^+^ (calcd for C_20_H_31_O_6_, 367.2115), corresponding to six degrees of unsaturation. Detailed inspection of the 1D NMR data of **2** (Table 1 and Table 2) showed they were similar to those of **1**, suggesting **2** was also a diterpene compound. When 1D and 2D NMR data were analyzed, a distinct difference was found in the fragment of C-9 to C-14 between **2** and **1**. The presence of the three spin systems in **2** (Figure 3) was confirmed by the ^1^H-^1^H COSY correlations of H_2_-4/H_2_-5/H-6, H_2_-8/H-9/H-10 and H_2_-12/H_2_-13/H-14. Both spin systems were connected to an oxygenated tertiary carbon at C-11 (*δ*_C_ 84.2) by a C-C single bond of C-10-C-11 and C-11-C-12, respectively, as evidenced by key HMBC correlations for H_3_-18 (*δ*_C/H_ 27.4/1.30) to C-10 (*δ*_C_ 138.1), C-11 (*δ*_C_ 84.2) and C-12 (*δ*_C_ 38.5). A *gem*-dimethyl group was also confirmed to be localized at an oxygenated tertiary carbon C-15 (*δ*_C_ 72.7) and the group is attached to an oxygenated methine carbon of C-14, evidenced by HMBC correlations from H_3_-16 (*δ*_C/H_ 25.7/1.15)/17 (*δ*_C/H_ 25.8/1.17) to C-14 (*δ*_C_ 86.6), C-15 (*δ*_C_ 72.7). Considering one degree of unsaturation remaining, and the extreme downfield chemical shifts of two oxygenated carbons at *δ*_C_ 84.2 (C-11) and *δ*_C_ 86.6 (C-14) (Figure 3), a tetrahydrofuran moiety was deduced to establish the planar structure. Thus, compound **2** was a tetrahydrofuran-containing monocyclic diterpene.

The *E*-configuration of double bonds at C-6/C-7 and C-9/C-10 was easily assigned through significant spatial NOE correlations observed between H_3_-19/H_2_-5, H-6/H_2_-8, H_2_-8/H-10 and H-9/H_3_-18. The key NOESY correlations between H-10/H-14 and H_3_-18/H_3_-16 allowed us to determine the relative configuration of tetrahydrofuran as 11S*, 14S* (Figure 4).

**Figure 5:**
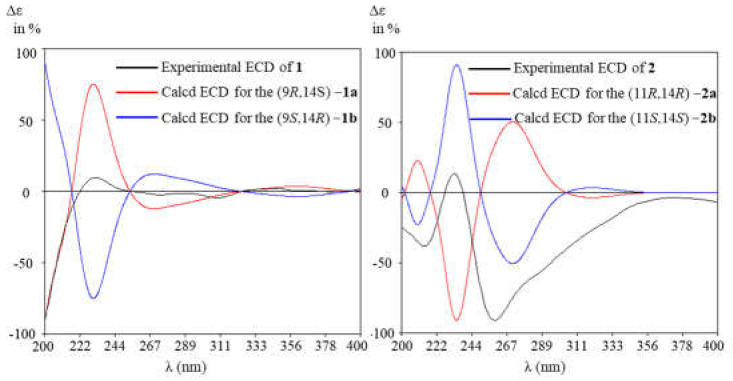
Experimental and calculated ECD spectra of **1** and **2** (in MeOH).

To determine the absolute configuration of **1** and **2**, the ECD calculations on the optimized conformation of (2*E*, 6*E*, 9*R*, 14*S*)−**1** and (2*E*, 6*E*, 9*E*, 11*R*, 14*R*)−**2** obtained at the B3LYP/6-31+G(d) level were performed. The overall pattern of the experimental ECD spectrum was in reasonable agreement with the calculated spectrum of (2*E*, 6*E*, 9*R*, 14*S*)−**1a** and (2*E*, 6*E*, 9*E*, 11*S*, 14*S*)−**2b** (Figure 5), thereby confirming the absolute configuration of (2*E*, 6*E*, 9*R*, 14*S*)−**1** and (2*E*, 6*E*, 9*E*, 11*S*, 14*S*)−2, respectively.

Schizostatin K (**3**) was isolated as yellow oil and the molecular formula was determined as C_20_H_32_O_7_ by the HR-ESI-MS ions at *m*/*z* 385.2220 [M + H]^+^ (calcd for C_20_H_33_O_7_, 385.2219), indicating five degrees of unsaturation. Comparison of NMR data of **3** and those of **2** revealed that the skeleton outline of **3** is very similar to that of **2**. The only difference was that the double bond at C-9/C-10 which disappeared in **2** was replaced by the OH-10 group at C-10 in **3**, supported by COSY correlations of H_2_-8/H_2_-9/H-10 and HMBC correlations of H_2_-9 (*δ*_C/H_ 77.1/1.34, 1.71) with C-11 (*δ*_C_ 86.8) and H_3_-18 (*δ*_C/H_ 23.0/1.13) with C-10 (*δ*_C_ 77.1)**.** The relative configuration of tetrahydrofuran moiety was deduced by the NOESY correlation of H_3_-18/H_3_-16. Considering the common biosynthetic origin, the absolute configuration of both C-14 and C-11 in **3** was proposed as *S*, which is consistent with those of **2**.

Schizostatin E (**4**) was obtained as white powder, and was found to be C_17_H_24_O_5_, based on positive HR-ESI-MS at *m*/*z* 309.1691 [M + H]^+^, indicating it was a truncated diterpene, like compound **13**. The unsaturation degree of compound **4** is 6. Analysis of the 1D NMR data of **4** (Table 3) with **2** revealed they shared a similar framework, and the tetrahydrofuran unit was also present, which was evidenced by the key HMBC correlations from H_2_-14 (*δ*_C/H_ 68.4/3.85) to C-11 (*δ*_C_ 83.9) and C-12 (*δ*_C_ 38.6). The major difference between the NMR spectra of **4** and those of **2** was that the *gem*-dimethyl group at C-15-C-17 which appeared in **2** disappeared in **4**, evidenced by the presence of oxygenated methylene carbon, rather than oxygenated methine carbon, as well as 2D data. The C-6/C-7 and C-10/C-11 double bonds could be assigned as *E*-configuration on the basis of NOESY correlations of H_3_-16/H_2_-5, H-6/H_2_-8, H_2_-8/H-10 and H-9/H_3_-15 (Figure 4).

**Figure 6:**
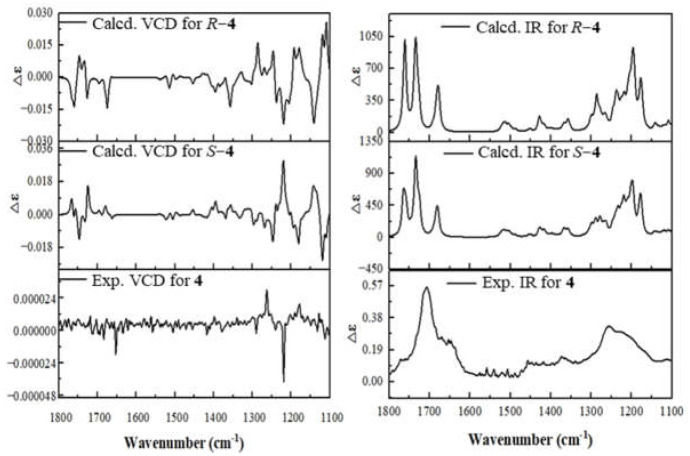
The VCD (left) and IR (right) spectra of compound **4** (measures in DMSO) with those calculated for 11*R*−**4**, 11*S*−**4**.

To assign the absolute configuration of **4**, the VCD calculations were performed. Conformational search for molecule **4** was carried out using the MMFF94 force field by the MOE 2019.01 software (Chemical Computing Group ULC). DFT calculations were used to optimize the conformers at the B3LYP/6-31G(d) and B3LYP/6-311+G(d) levels, respectively. We calculated IR and VCD spectra for (11*R*)−**4** and (11*S*)−**4**. The VCD calculations for **4** were carried out at the B3LYP/6-311+G(d) level in the gas phase. Boltzmann statistics were used for final simulations of the VCD for these molecules. The VCD calculation simulation shows that the signals at 1247~1412 cm^−1^ are related to the vibrations of the calculated chiral carbon positions, and a comparison of the calculated values of the VCD signals for the *R* and *S* configurations at 1247~1412 cm^−1^ with the experimental values at 1213~1300 cm^−1^ showed that the configuration *S* is more consistent with the experimental values [21,22] (Figure 6). Thus, the absolute configuration of **4** was determined as 2*E*, 6*E*, 10*E*, 11*S*.

Schizostatin F (**5**) was obtained as yellow oil. The molecular formula was determined as C_17_H_26_O_6_ based on the HR-ESI-MS ions at *m*/*z* 327.1800 ([M + H]^+^, calcd for C_17_H_27_O_6_, 327.1802). Detailed analyses of its ^1^H and ^13^C NMR (Table 1 and Table 2) and 2D NMR spectra revealed the structure of **5** was almost identical to that of **4**. The only difference between **5** and **4** is that the double-bond group of C-9/C-10 in **4** disappeared and was replaced by the OH-10 group at C-10 in **5**. The total *E*-geometry of the double bond of C-6/C-7 was confirmed by key NOESY correlations of H_3_-19/H_2_-5 and H-6/H_2_-8 (Figure 4). Thus, **5** was also a truncated and tetrahydrofuran-containing monocyclic diterpene. However, the Mosher reaction was not carried out, so that the absolute configuration of OH-10 was not determined. Considering the common biosynthesis between **5** and **2**, the absolute configuration of C-11 in **5** was proposed to be *S.*

Schizostatin G (**6**) was obtained as yellow oil. The molecular formula of C_20_H_28_O_5_ was determined according to the HR-ESI-MS ions at *m*/*z* 349.2006 [M + H]^+^ (calcd for C_20_H_29_O_5_, 349.2009). Detailed inspection of 1D NMR (Table 1 and Table 2) revealed the structure of **6** was similar to that of **14**. The main difference between NMR data **6** and **14** is that the methyl group of **14** is replaced with oxygenated methylene carbon at the C-18 position, and the sp^3^ methylene group at the C-13 position is replaced by oxygenated methine carbon. The HMBC correlations (Figure 3) from H_2_-18 (*δ*_C/H_ 69.0/4.38, 4.22) to C-12 (*δ*_C_ 40.2), C-11 (*δ*_C_ 139.7), C-10 (*δ*_C_ 121.3), C-13 (*δ*_C_ 77.5) and from H-13 (*δ*_C/H_ 77.5/4.57) to C-15 (*δ*_C_ 138.0), H_2_-12 (*δ*_C/H_ 40.2/2.59, 2.20) to C-10 (*δ*_C_ 121.3), along with the COSY correlations between H_2_-12, H-13 and H-14 allowed us to determine the presence of a tetrahydrofuran in compound **6**. The NOESY correlations of H_2_-18/H_2_-9 and H-10/H_2_-12 provided the evidence for *Z*-configuration of C-10/C-11. The optical rotation calculations were carried out to ascertain the exact configuration of C-1**3** at the B3LYP/6-31+G(d) level. Comparing the calculated optical rotation of *R*-**6** (+234.90) with the experimental data of **6**-(+4.21), the absolute configuration of C-1**3** was assigned as *R*. Thus, the absolute configuration of **6** was determined as 2*E*, 10*Z*, 13*R*.

All the compounds **7**–**11** were observed to contain twenty carbons and are noncyclic, as deduced from their HRESIMS and 1D NMR spectra (Table 1 and Table 2), indicative of the fact that they possessed an unabridged linear diterpene-type skeleton. The detailed interpretation of 2D NMR spectra of **7**–**11** established the identical fragments from C-1 to C-9, including the existence of the butenedioic acid moiety, and the *E*-configuration of the double bond of C-6/C-7 (Figure 4). The structures of **7**–**11** should be oxidized variants of schizostatin A (**14**), a potent squalene synthase inhibitor which was also co-isolated from the fungus.

Schizostatin H (**7**) and schizostatin I (**8**) were obtained as yellow oil and were found to be C_20_H_32_O_6_ and C_20_H_32_O_7_, based on positive HR-ESI-MS at *m*/*z* 351.2173 [M-H_2_O + H]^+^ and 385.2219 [M + H]^+^ (calcd for C_20_H_31_O_5_, 351.2166 for **7**; 385.2221 for **8**), respectively. A careful comparison of 1D NMR data between **7** and **15** showed an almost identical planar structure (Table 1 and Table 2), except for the replacement of the double bond of C-14/C-15 in **15** with the pinacol group in **7**, evidenced by the key HMBC correlations from H_3_-17 (*δ*_C/H_ 25.0/1.12)/ H_3_-16 (*δ*_C/H_ 25.6/1.15) to C-15 (*δ*_C_ 73.8) and C-14 (*δ*_C_ 79.1), as well as the COSY correlations of H_2_-12/H_2_-13/H-14 (Figure 3). The structure of schizostatin I (**8**) was determined to be very similar to that of **7**, except for the loss of the signal corresponding to the methylene carbon at C-12, and replacement with an oxygenated methine carbon in **8**. It was confirmed by correlations of H-12/H_2_-13/H-14 in the COSY spectrum and of Me-18 (*δ*_C/H_ 11.0/1.61) with C-10 (*δ*_C_ 128.2), C-11 (*δ*_C_ 137.0) and C-12 (*δ*_C_ 78.0) in the HMBC spectrum. The absolute configuration of C-14 in both **7** and **8** was proposed as *S*, which was deduced by the biosynthetically related product **2**.

The molecular formula of C_20_H_32_O_6_ of schizostatin J (**9**) was same as that of Schizostatin H (**7**). Comparison of NMR data between **9** and **7** indicated that the structure of **9** is similar to that of **7.** The difference is that the pinacol group was positioned at C-10 and C-11 and the double bond at C-14/C-15 remained, supported by the related COSY and HMBC correlations. Due to the limited amount, the absolute configuration of C-10 and C-11 was unresolved.

Schizostatin K (**10**) was obtained as yellow oil with the molecular formula of C_20_H_32_O_5_ from the HR-ESI-MS (*m*/*z*, 353.2325 [M + H]^+^, calcd for C_20_H_33_O_5_, 353.2322). According to 1D and 2D NMR, compound **10** is structurally very similar to compound **14**, except for the loss of the signal of the double bond at C-14/C-15, and replacement with two sp^3^ carbons, evidenced by COSY correlations of H-12 to H-17 and the key HMBC correlation from H_2_-12 (*δ*_C/H_ 40.8/2.08), H_2_-16 (*δ*_C/H_ 68.5/3.30, 3.41) and H_3_-17 (*δ*_C/H_ 17.1/0.89) to C-14 (*δ*_C_ 33.9) (Figure 3). Moreover, a methyl group was oxidized to a hydroxymethyl group, which was determined via the COSY correlation of H-16/H-15/H-17, and the HMBC correlation of H_3_-17 (*δ*_C/H_ 17.1/0.89) with C-16 (*δ*_C_ 68.5). Due to limited stockage and lack of an adjacent chromophore group at C-15 (*δ*_C_ 36.8), the absolute configuration of C-15 was unresolved.

The molecular formula of C_20_H_30_O_5_ of schizostatin L (**11**) was the same as that of **12**. Comparison of NMR data between **11** and **12** indicated that the structure of **11** was almost identical to that of **12,** with the difference in chemical shifts of C-16 (*δ*_C/H_ 61.4/4.05 for **11** vs. *δ*_C/H_ 69.0/3.9 for **12**) and C-17 (*δ*_C/H_ 21.5/1.75 for **11** vs. *δ*_C/H_ 13.7/1.6 for **12**). The strong NOESY correlations of H_3_-17/H-14 and H_2_-16/H_2_-13 allowed us to assign the *Z*-configuration of C-14/C-15.

In addition, three known compounds, (2E)-2-[(3E, 7E, 11E)-13-Hydroxy-4,8,12-trimethyl-3,7,11-tridecatrien-1-yl]-2-butenedioic acid (**12**), (2E)-2-[(3E,7E)-11-Hydroxy-4,8-dimethyl-3,7-undecadien-1-yl]-2-butenedioic acid (**13**) [22], and schizostatin A [23] (**14**), were also co-isolated and were determined based on comparisons of NMR data with previously reported data.

Compounds **1**–**14** were tested for cytotoxic, antimicrobial and OH scavenging activities. None of them displayed any cytotoxic activity at 10 µM. Compound **1** showed potent antifungal activity against plant pathogenic fungi *Colletotrichum camelliae* with MIC values of 8 μg/mL, while others did not exhibit any antifungal activity at a concentration of 64 μg/mL.

At a concentration of 50 μM, the inhibition rates of compounds **1**–**14** ranged from 42.3% to 76.0%. For comparison, the inhibition rate of the positive control, V_C,_ was 81.69%, which is shown in Table 4. The hydroxyl radical scavenging data suggested that compounds **1**–**14** are beneficial for the interest in preservation of foodstuffs, drug products and cosmetics.

## 3. Materials and Methods

### 3.1. General Experimental Procedures

NMR spectra (in ppm, *J* in Hz) were measured using a Bruker AVANCE Neo 600 MHz spectrometer (Bruker BioSpin, Fällanden, Switzerland), with CD_3_OD and TMS as the internal standard. HR-ESI-MS spectra were acquired with a Q-Exactive Orbitrap tandem mass spectrometer (Q-Exactive Orbitrap-MS) (Thermo Scientific, Bremen, Germany). Electric Circular Dichroism (ECD) spectra were obtained using a Jasco J1500 spectrometer (Jasco Inc., Tokyo, Japan). A JASCO P-1020 digital polarimeter (JASCO Corporation, Tokyo, Japan) was used to obtain optical rotations. IR spectra were acquired using Fourier Transform Infrared Spectrometer IEC/EN 60825-1 (Thermo Fisher Scientific, Madison, WI, USA). High-performance liquid chromatography (HPLC) 1260 system (Agilent, Santa Clara, CA, USA), and a chromatographic column (xbridge-C18, Thermo Fisher Scientific, Waltham, MA, USA) with a pipe diameter of 5 μm, 4.6 mm × 250 mm, were used to analyze the samples at room temperature. An ODS column (PRC-ODS EC0704, 50 mm × 250 mm, 15 μm, 8 mL/min, Shimadzu, Kyoto, Japan) served as the stationary phase in preparative HPLC and a C18 column (FWXB12S05-2510, 10 mm × 250 mm, 5 µm, 4 mL/min, Thermo Fisher Scientific, Waltham, MA, USA) was used for the stationary phases in semi-preparative HPLC. The 200–300-mesh silica gel (Nuotai Biotechnology Co., Ltd, Shan Xi, China), ODS (50 μm, YMC, Kyoto, Japan), and TOYOPEARL HW-40F (TOSOH, Tokyo, Japan) were employed for column chromatography (CC). The solvents for CC were of analytical grade.

### 3.2. Fungi Materials

The strain NJFU21 was isolated from the larval intestines of the cutworm, which was collected from the Zi Jin Mountain in Nanjing, Jiangsu province, China, in 2020. The voucher specimen has been deposited in our laboratory collection. The fungus was identified as *Schizophyllum commune* through sequence-based rDNA ITS region analysis, as evidence by its GenBank accession number OP761877 (Supporting Information).

### 3.3. The Screening of Elicitors

Five elicitors with various regulatory mechanisms were selected in the study, including sodium laurate, SAHA, 5-Aza, *p*-(trifluoromethyl)aniline and aniline. Each elicitor was screened individually to determine a suitable concentration for *S. commune* NJFU21, acting as a control. Following this, the selected elicitors were combined and added into the fermentation of *S. commune* NJFU21 in the suitable concentration, individually. HPLC analysis was conducted to analyze and compare the changes in the metabolites of *S. commune* NJFU21 among themselves.

### 3.4. Fermentation and Extraction

*S. commune* NJFU21 was cultured on potato dextrose agar at 28 °C for 7 days. Fresh mycelium blocks (3 mm × 3 mm, 1 block per flask) were then added to seventy 1000 mL Erlenmeyer flasks, each containing 80.0 g of rice, 120 mL of H_2_O, and a combination of five selected elicitors: 0.1 mM (Sodium Laurate, Aniline and *p*-Trifluoromethylaniline), 0.075 mM (SAHA), and 0.05 mM (5-Aza). After 30 days of cultivation, the secondary metabolites were extracted with an equal volume of ethyl acetate, three times. The ethyl acetate extract was then concentrated under reduced pressure to yield 60.4 g of crude extract.

The crude extract was separated by CC with gradient elution of CH_2_Cl_2_–MeOH to obtain eight fractions (Fr.1–Fr.8). The targeted fractions Fr. 6 (7.38 g) and Fr. 7 (6.50 g) were further separated and purified by silica gel ODS with gradient elution of H_2_O–MeOH, to give Fr. 6-1 to Fr. 6-12 and Fr. 7-1 to Fr. 7-12, respectively. Subsequently, Fr. 6-2 (533.8 mg) and Fr. 7-1 (356.8 mg) were subjected to further purification using silica gel HW-40F and 100% MeOH (1.5 L) to yield targeted fractions Fr. 6-2-10 (316.7 mg), Fr. 6-2-15 (**14**, 125.6 mg) and Fr. 7-1-8 (106.3 mg).

Fr. 6-2-10 was separated by preparative HPLC (MeOH–H_2_O, 55–85%, 8 mL/min) to obtain six subfractions (Fr. 6-2-10-1–Fr. 6-2-10-6), followed by the purifying of Fr. 6-2-10-1–Fr. 6-2-10-5 by semi-preparative HPLC (35:65 MeCN–H_2_O, 4 mL/min) to afford compounds **2** (1.9 mg, t_R_ = 31 min), **4** (5.6 mg, t_R_ = 33 min), **13** (4.4 mg, t_R_ = 30 min), **1** (3.5 mg, t_R_ = 43 min), **7** (2.9 mg, t_R_ = 28 min) and **9** (2.4 mg, t_R_ = 31 min). Fr. 6-2-10-6 was purified by semi-preparative HPLC (42:58, MeCN–H_2_O, 4 mL/min) to afford compounds **12** (3.5 mg, t_R_ = 41 min), **11** (1.3 mg, t_R_ = 49 min), **5** (1.7 mg, t_R_ = 43 min) and **6** (1.9 mg, t_R_ = 56 min). Likewise, Fr. 7-1-8 was further purified by preparative HPLC eluting with a linear gradient (MeOH–H_2_O, 55–85%), giving three subfractions (Fr. 7-1-8-1–Fr. 7-1-8-3), followed by the purifying of Fr. 7-1-8-1–Fr. 7-1-8-3 by semi-preparative HPLC (23:77 MeCN–H_2_O, 4 mL/min) to obtain compounds **8** (2.1 mg, t_R_ = 39 min), **3** (3.5 mg, t_R_ = 45 min), and **10** (2.4 mg, t_R_ = 57 min).

Schizostatin B (**1**): white powder (MeOH); [*α*] ^25^_D_ + 14.86(*c* 0.35, MeOH); UV (CH_3_CN) *λ*_max_ (log *ε*) 210 (1.67) nm; ECD (0.5 mM, MeOH) *λ*_max_ (Δ *ε*) 219 (−3.05) nm, 240 (−0.5) nm, 240 (-0.5) nm; IR (KBr) *ν*_max_: 3405, 1647, 1454, 1053, 1032, 1018 and 656 cm^−1^; ^1^H and ^13^C NMR data, see Table 1 and Table 2; HR-ESI-MS: [M + H]^+^ *m*/*z*, 351.2174 (calcd, 351.2166).

Schizostatin C (**2**): yellow oil (MeOH); [*α*] ^25^_D_ + 29.47(*c* 0.19, MeOH); *λ*_max_ (log *ε*) 202 (2.12) nm; ECD (0.6 mM, MeOH) *λ*_max_ (Δ *ε*) 233 (0.48) nm; IR (KBr) *ν*_max_: 3389, 1648, 1453, 1032 and 657 cm^−1^; ^1^H and ^13^C NMR data, see Table 1 and Table 2; HR-ESI-MS: [M + H]^+^ *m*/*z*, 367.2108 (calcd, 367.2115).

Schizostatin D (**3**): yellow oil (MeOH); [*α*] ^25^_D_ 30(*c* 0.24, MeOH); *λ*_max_ (log *ε*) 200 (2.52) nm; IR (KBr) *ν*_max_: 3405, 2518, 2075, 1647, 1020 and 641 cm^−1^; ^1^H and ^13^C NMR data, see Table 1 and Table 2; HRESI-MS: [M + H]^+^ *m*/*z*, 385.2220 (calcd, 385.2219).

Schizostatin E (**4**): white powder (MeOH); [*α*] ^25^_D_ + 29.47(*c* 0.1, MeOH); *λ*_max_ (log *ε*) 200 (2.13) nm; IR (KBr) *ν*_max_: 3386, 1650, 1454, 1413, 1032 and 657 cm^−1^; ^1^H and ^13^C NMR data, see Table 3; HRESI-MS: [M + H]^+^ *m*/*z*, 309.1691 (calcd, 309.1696).

Schizostatin F (**5**): yellow oil (MeOH); [*α*] ^25^_D_ 1.9(*c* 0.21, MeOH); *λ*_max_ (log *ε*) 194 (3.10) nm; IR (KBr) *ν*_max_: 3447, 1636, and 537 cm^−1^; ^1^H and ^13^C NMR data, see Table 3; HRESI-MS: [M + H]^+^ *m*/*z*, 327.1800 (calcd, 327.1802).

Schizostatin G (**6**): yellow oil (MeOH); [*α*] ^25^_D_ 4.21(*c* 0.19, MeOH); *λ*_max_ (log *ε*) 200 (1.63) nm; IR (KBr) *ν*_max_: 3405, 2949, 2843, 1648, 1053, 1033, 1019 and 656 cm^−1^; ^1^H and ^13^C NMR data, see Table 1 and Table 2; HRESI-MS: [M + H]^+^ *m*/*z*, 349.2006 (calcd, 349.2009).

Schizostatin H (**7**): yellow oil (MeOH); [*α*] ^25^_D_ −16.55(*c* 0.29, MeOH); *λ*_max_ (log *ε*) 200 (1.01) nm; IR (KBr) *ν*_max_: 3440, 1640, 1054, 1033, 1015 and 599 cm^−1^; for ^1^H and ^13^C NMR data, see Table 1 and Table 2; HRESI-MS: [M-H_2_O + H]^+^ m/z, 351.2173 (calcd, 351.2166).

Schizostatin I (**8**): yellow oil (MeOH); [*α*] ^25^_D_ 3.43(*c* 0.35, MeOH); *λ*_max_ (log *ε*) 200 (4.49) nm; IR (KBr) *ν*_max_: 3452, 1640, 1054, 1033, 1015 and 591 cm^−1^; ^1^H and ^13^C NMR data, see Table 1 and Table 2; HRESI-MS: [M + H]^+^ *m*/*z*, 385.2219 (calcd, 385.2221).

Schizostatin J (**9**): yellow oil (MeOH); [*α*] ^25^_D_ −7.03(*c* 0.74, MeOH); *λ*_max_ (log *ε*) 200 (2.14) nm; IR (KBr) *ν*_max_: 3451, 1636, 1054, 1033, 1015 and 576 cm^−1^; ^1^H and ^13^C NMR data, see Table 1 and Table 2; HRESI-MS: [M-H_2_O + H]^+^ *m*/*z*, 351.2159 (calcd, 351.2166).

Schizostatin K (**10**): white powder (MeOH); [*α*] ^25^_D_ 20(*c* 0.03, MeOH); *λ*_max_ (log *ε*) 200 (2.46) nm; IR (KBr) *ν*_max_: 3444, 1640, 1053, 1033, 1016 and 626 cm^−1^; ^1^H and ^13^C NMR data, see Table 1 and Table 2; HRESI-MS: [M + H]^+^ *m*/*z*, 353.2325 (calcd, 353.2322).

Schizostatin L (**11**): white powder (MeOH); [*α*] ^25^_D_ 8(*c* 0.07, MeOH); *λ*_max_ (log *ε*) 200 (1.26) nm; IR (KBr) *ν*_max_: 3386, 2949, 2843, 1648, 1454, 1032 and 656 cm^−1^; ^1^H and ^13^C NMR data, see Table 1 and Table 2; HRESI-MS: [M + H]^+^ *m*/*z*, 351.2175 (calcd, 351.2166).

### 3.5. Nuclear Magnetic Resonance (NMR) Calculation Assay

Conformational searches were employed by Spartan’14 (Wavefunction Inc., Irvine, CA, USA), based on the MMFF94. All conformers were optimized with DFT calculations at the B3LYP/6-31+G(d) level by using the Gaussian 09 program. NMR calculation of **1** was performed at B3LYP/6-31+G (d)//B3LYP/6-311+G (d, p) levels and further checked by DP4+ probability [24].

### 3.6. Electric Circular Dichroism (ECD) Calculation Assay

Conformational searches were run employing the ‘systematic’ procedure implemented in Spartan’14, based on the MMFF (Merck Molecular Force Field). All conformers were further optimized with Density functional theory (DFT) calculations at the B3LYP/6-31+G(d) level by using the Gaussian 09 program [25].

### 3.7. Regarding the Optical Rotation (OR) Calculation Assay

For OR computation, all conformers of compound **6** were first optimized at the B3LYP/6-31+G(d) in MeOH (PCM). DFT at the B3LYP/6-31+G(d) level in Gaussian 09 was used to theoretically calculate the OR in MeOH for each conformer of compound **6** [26].

### 3.8. Vibrational Circular Dichroism (VCD) Calculation Assay

The conformational search for the molecule *R*-compound-**4** and *S*-compound-**4** was carried out using the MMFF94 force field by the MOE 2019.01 software (Chemical Computing Group ULC). A total of 129 stable conformers for *R*-compound-**4** and 140 stable conformers for *S*-compound-**4** were recorded with relative energy within a 5 kcal/mol energy window. DFT calculations were used to optimize the conformers at the B3LYP/6-31G(d) and B3LYP/6-311+G(d) levels, respectively. The VCD calculations for the stable conformers were performed by Gaussian 09 (Gaussian Inc., Wallingford, CT, USA) software. VCD calculations for *R*-compound-**4** and *S*-compound-**4** were carried out at the B3LYP/6-311+G(d) level in the gas phase. Boltzmann statistics were used for final simulations of the VCD for these molecules. The VCD spectrum obtained from *R*-compound-**4** and *S*-compound-**4** both have a half-peak width of 0.16 and a wavelength range of 1100 to 1800 [27].

### 3.9. Assay of Antimicrobial Activity

The major plant pathogenic fungi *C*. *camelliae* was used to evaluate the antifungal activity of each compound, with carbendazim serving as the benchmark control at a concentration of 64 μg/mL in DMSO. The compounds were dissolved in DMSO to generate 128 mg/mL stock solutions. A total of 1 × 10^5^ cells/mL of *C*. *camelliae* were inoculated into each well of a 96-well plate [28]. Subsequently, the stock solutions were then serially diluted with PDB liquid medium to afford working concentrations of 128 to 2 μg/mL. Following a 48 h incubation at 28 °C, we determined the minimum inhibitory concentration (MIC) based on the growth outcomes in the 96-well plates. Carbendazim was used as a positive control.

### 3.10. Hydroxyl Radical Scavenging Assay

The salicylate technique was utilized to assess the hydroxyl radical scavenging capacity [29,30]. The hydroxyl radical scavenging test was performed in 96-well microplates. Twenty-five microliters of samples (200 μM) was mixed with 25 μL of FeSO_4_·7H_2_O (9 mM) and 25 μL of ethanolic salicylic acid (9 mM), which were thoroughly mixed using a vortex mixer. Subsequently, 25 μL of H_2_O_2_ (8.8 mM) was added to the reaction mixture and then incubated at 37 °C for 30 min. Vitamin C was prepared as positive control and the absorbance was measured at 510 nm.

The hydroxyl radical scavenging percentage was calculated using the following equation:

Scavenging activity(%) = [OD_(control)_ − (OD_(sample)_ − OD_(blank)_)]/OD_(control)_ × 100%

## 4. Conclusions

In conclusion, with the aim of assessing the practical value and potential risk of the traditionally medicinal and edible higher fungus *Schizophyllum commune*, induced by natural undescribed small molecules, we implemented a combination strategy of elicitors to characterize products of cryptic and extremely low-expressed gene clusters. The resulting combination of five elicitors with different concentrations and induction mechanism was selected and added into fermentation medium. As a result, we were able to significantly upregulate a class of linear diterpene-derived metabolites. In total, fifteen linear diterpene-derived variants were isolated and identified, including eleven new ones and three known ones. All the isolated terpenes contain an unusual butenedioic acid moiety in the elongation terminus. Compound **1** was a rare monocyclic diterpene, while **2**–**6** possessed a tetrahydrofuran moiety. The compounds **4**, **5** and **13** are considered as trinorditerpene-type metabolites. In contrast to polycyclic terpene products, the linear terpene-derived products are unusual in nature. Recently, the Zou group [31] had been characterized as a globin-like enzyme, TutaA, in the *Schizophyllum commune*, which is responsible for truncating schizostain A (**14**) to form trinorditerpene product 2-butenedioic acid (**13**), indicating that the production of the new truncated compounds **4** and **5** is probably involved in a similar formation mechanism.

All the diterpene compounds displayed the ability for the scavenging of hydroxyl radicals and showed null cytotoxic activity at 10 µM. Considering that the crude extract of *Schizophyllum commune* has been approved as a cosmetic ingredient in China, the diterpenes would be beneficial protectants for a cosmetic ingredient. In addition, compound **1** showed potent antifungal activity, which highlights the potential of **1** as a lead compound for a novel agricultural fungicide development.

## Figures and Tables

**Table 1:** ^1^H NMR (600 MHz) Spectroscopic Data for compounds **1**–**3** and **6**–**11** in CD_3_OD (*δ* in ppm, *J* in Hz).

	1	2	3	6	7	8	9	10	11
Position	*δ*_H_, (*J* in Hz)	*δ*_H_, (*J* in Hz)	*δ*_H_, (*J* in Hz)	*δ*_H_, (*J* in Hz)	*δ*_H_, (*J* in Hz)	*δ*_H_, (*J* in Hz)	*δ*_H_, (*J* in Hz)	*δ*_H_, (*J* in Hz)	*δ*_H_, (*J* in Hz)
2	6.72, s	6.72, s	6.72, s	6.71, s	6.71, s	6.72, s	6.71, s	6.72, s	6.71, s
4	2.79, t (7.6)	2.80, t (7.6)	2.79, t (7.5)	2.79, t (7.6)	2.78, t (7.7)	2.79, t (7.6)	2.78, t (7.6)	2.78, t (7.6)	2.78, t (7.6)
5	2.19, q, (7.6)	2.20, q, (7.5)	2.19, m	2.18, m	2.17, q, (7.6)	2.18, m	2.18, m	2.17, q, (7.6)	2.17, m
6	5.11, t, (7.5)	5.18, t, 7.4	5.21, m	5.17, m	5.14, m	5.18, t, (7.4)	5.22, t, (7.4)	5.15, m	5.12, m
8	1.77, m, 2.43, m ^a^	2.66, d, (6.6)	2.02, m, 2.21, m	2.01, m ^a^	1.97, m ^a^, 2.08, m ^a^	2.13, m ^a^, 2.02, m ^a^	1.98, m, 2.24, m	1.97, m ^a^	1.97, m ^a^
9	2.43, m ^a^	5.55, m	1.34, m, 1.71, m	2.01, m ^a^	1.97, m ^a^, 2.08, m ^a^	2.13, m ^a^, 2.02, m ^a^	1.37, m, 1.73, m	2.07, m, 1.97, m ^a^	2.07, q, (7.5)
10	5.33, m	5.49, m	3.39, m	5.30, m	5.17, m	5.43, t, (6.6)	3.26, m	5.11, m	5.16, m
12	1.99, m	1.87, m ^a^, 1.68, m	1.59, m, 2.04, m	2.59, m, 2.20, m	2.22, m, 1.99, m	4.22, t, (7.0)	1.45, m, 1.52, m	2.08, q, (7.3)	1.97, m ^a^
13	1.55, m, 1.75, m	1.87, m ^a^	1.82, m	4.57, m	1.33, m, 1.70, m	1.78, m, 1.54, m	2.07, m	1.46, m, 1.37, m ^a^	2.14, m,
14	1.58 (m)	3.80 (t, 6.7)	3.75, dd (9.7, 5.9)	5.20 (m)	3.22, m	3.28 (m)	5.11, m	1.05, m, 1.37, m ^a^	5.26, m
15								1.55, m	
16	1.25, s	1.15, s	1.17, s	1.70, s	1.15, s	1.15, s	1.62, s	3.30, m, 3.41, m	4.05, m
17	1.23, s	1.17, s	1.14, s	1.73, s	1.12, s	1.13, s	1.67, s	0.89, d, (6.7)	1.75, q, (1.3)
18	1.61, s	1.30, s	1.13, s	4.38, m, 4.22, m	1.62, s	1.61, s	1.10, s	1.59, s	1.59, s
19	1.63, s	1.57, s	1.61, s	1.59, s	1.60, s	1.61, s	1.61, s	1.59, s	1.60, s

^a^ Signals were overlapped.

**Table 2:** ^13^C NMR (150 MHz) Spectroscopic Data for compounds **1**–**3** and **6**–**11** in CD_3_OD (*δ* in ppm).

	1	2	3	6	7	8	9	10	11
Position	*δ*_C_, Type	*δ*_C_, Type	*δ*_C_, Type	*δ*_C_, Type	*δ*_C_, Type	*δ*_C_, Type	*δ*_C_, Type	*δ*_C_, Type	*δ*_C_, Type
1-COOH	170.1, C	170.1, C	170.2, C	170.2 ^a^, C	170.2 ^a^, C	170.3, C	170.3, C	170.2, C	170.3, C
2	128.3, CH	128.4, CH	128.4, CH	128.6, CH	128.4, CH	128.4, CH	128.4, CH	128.3, CH	128.6, CH
3	148.6, C	148.4, C	148.4, C	148.3 ^a^, C	148.5, C	148.5, C	148.4, C	148.5, C	148.5, C
4	28.7, CH_2_	28.6, CH_2_	28.8, CH_2_	28.8, CH_2_	28.8, CH_2_	28.7, CH_2_	28.8, CH_2_	28.8, CH_2_	28.8, CH_2_
5	28.6, CH_2_	28.6, CH_2_	28.6, CH_2_	28.6, CH_2_	28.7, CH_2_	28.6, CH_2_	28.7, CH_2_	28.7, CH_2_	28.7, CH_2_
6	125.6, CH	125.0, CH	124.6, CH	124.9, CH	124.5, CH	124.5, CH	124.5, CH	125.4, CH	125.7, CH
7	135.9, C	136.3, C	137.2, C	136.7, C	137.2, C	137.6, C	137.4, C	137.2, C	137.2, C
8	42.5, CH_2_	43.4, CH_2_	37.5, CH_2_	40.3, CH_2_	40.8, CH_2_	40.4, CH_2_	38.0, CH_2_	41.0, CH_2_	40.8, CH_2_
9	35.5, CH	126.8, CH	30.9, CH_2_	28.9, CH_2_	27.6, CH_2_	27.2, CH_2_	30.4, CH_2_	27.6, CH_2_	27.6, CH_2_
10	127.4, CH	138.1, CH	77.1, CH	121.3, CH	125.6, CH	128.2, CH	78.2, CH	124.4, CH	124.4, CH
11	133.6, C	84.2, C	86.8, C	139.7, C	136.1, C	137.0, C	75.5, C	136.1, C	135.7, C
12	32.9, CH_2_	38.5, CH_2_	34.7, CH_2_	40.2, CH_2_	37.9, CH_2_	78.0, CH	39.4, CH_2_	40.8, CH_2_	41.0, CH_2_
13	20.5, CH_2_	27.5, CH_2_	27.8, CH_2_	77.5, CH	30.9, CH_2_	36.9, CH_2_	23.0, CH_2_	26.5, CH	27.2, CH_2_
14	49.5, CH	86.6, CH	88.1, CH	125.9, CH	79.1, CH	78.0, CH	126.1, CH	33.9, CH_2_	128.6, CH
15	73.4, C	72.7, C	72.3, C	138.0, C	73.8, C	73.4, C	132.0, C	36.8, CH	135.7, C
16	29.2, CH_3_	25.7, CH_3_	26.4, CH_3_	18.3, CH_3_	25.6, CH_3_	25.6, CH_3_	17.7, CH_3_	68.5, CH_2_	61.4, CH_2_
17	27.7, CH_3_	25.8, CH_3_	25.1, CH_3_	25.9, CH_3_	25.0, CH_3_	24.9, CH_3_	25.9, CH_3_	17.1, CH_3_	21.5, CH_3_
18	23.7, CH_3_	27.4, CH_3_	23.0, CH_3_	69.0, CH_2_	16.2, CH_3_	11.0, CH_3_	21.9, CH_3_	16.1, CH_3_	16.1, CH_3_
19	16.4, CH_3_	16.1, CH_3_	16.0, CH_3_	16.0, CH_3_	16.1, CH_3_	16.1, CH_3_	16.1, CH_3_	16.0, CH_3_	16.1, CH_3_
20-COOH	169.0, C	169.0, C	169.1, C	169.2 ^a^, C	169.0 ^a^, C	169.2, C	169.2, C	169.0, C	169.2, C

^a^ The chemical shifts were determined by HMBC.

**Table 3:** ^1^H (600 MHz) and ^13^C (150 MHz) NMR Data for compounds **4**, **5** in CD_3_OD (*δ* in ppm, *J* in Hz).

	4		5	
Position	*δ*_C_, Type	*δ*_H_, (*J* in Hz)	*δ*_C_, Type	*δ*_H_, (*J* in Hz)
1-COOH		170.0, C		170.2, C
2	6.72, s	128.4, CH	6.71, s	128.4, CH
3		148.5, C		148.4, C
4	2.80, t, (7.5)	28.6, CH_2_	2.78, t, (7.6)	28.6, CH_2_
5	2.20, q, (7.5)	28.6, CH_2_	2.19, m ^a^	28.8, CH_2_
6	5.18, m	125.1, CH	5.21, m	124.6, CH
7		136.2, C		137.2, C
8	2.65, d, (6.4)	43.4, CH_2_	2.21, m ^a^, 2.00, m ^a^	37.6, CH_2_
9	5.54, m	127.3, CH	1.68, m, 1.34, m	31.1, CH_2_
10	5.50, m	137.8, CH	3.3, m	77.0, CH
11		83.9, C		86.8, C
12	1.72, m, 1.90, m ^a^	38.6, CH_2_	2.02, m ^a^, 1.58, m	34.5, CH_2_
13	1.92, m ^a^	26.5, CH_2_	1.93, m	27.1, CH_2_
14	3.85, m	68.4, CH_2_	3.84, m, 3.79, m	69.0, CH_2_
15	1.29, s	26.8, CH_3_	1.12, s	22.3, CH_3_
16	1.56, s	16.1, CH_3_	1.6, s	16.1, CH_3_
17-COOH		168.9		169.1

^a^ Signals were overlapped.

**Table 4:** Hydroxyl radical scavenging activities of compounds **1**–**14**.

Compounds	% Inhibition (50 µM)	Compounds and V_C_^a^	% Inhibition (50 µM)
1	76.0	9	71.2
2	70.4	10	68.0
3	71.4	11	69.6
4	69.2	12	67.7
5	71.0	13	71.7
6	71.7	14	73.4
7	71.4	V_C_^a^	81.7
8	72.6		

^a^ Positive control.

## Data Availability

The original contributions presented in the study are included in the article or Supplementary Material.

## Supplementary Materials

The following supporting information can be downloaded at: https://www.mdpi.com/article/10.3390/molecules29112608/s1, The NMR, HRESIMS, IR and VCD data for **1**–**14**.

## References

[1] Du B., Yang Y., Bian Z., Xu B. (2017). Characterization and Anti-Inflammatory Potential of an Exopolysaccharide from Submerged Mycelial Culture of *Schizophyllum commune*. Front. Pharmacol..

[2] Basso V., Schiavenin C., Mendonça S., de Siqueira F.G., Salvador M., Camassola M. (2020). Chemical features and antioxidant profile by *Schizophyllum commune* produced on different agroindustrial wastes and byproducts of biodiesel production. Food. Chem..

[3] Du B., Yang Y., Bian Z., Xu B. (2017). Molecular weight and helix conformation determine intestinal anti-inflammatory effects of exopolysaccharide from *Schizophyllum commune*. Carbohyd. Polym..

[4] Liu X., Frydenvang K., Liu H., Zhai L., Chen M., Olsen C.E., Christensen S.B. (2015). Iminolactones from *Schizophyllum commune*. J. Nat. Prod..

[5] (2023). National Medical Products Administration. https://www.nmpa.gov.cn.

[6] Gao J.M. (2006). New biologically active metabolites from Chinese higher fungi. Curr. Org. Chem..

[7] Tang H.Y., Yin X., Zhang C.C., Jia Q., Gao J.M. (2015). Structure diversity, synthesis, and biological activity of cyathane diterpenoids in higher fungi. Curr. Med. Chem..

[8] Bai R., Zhang C.C., Yin X., Wei J., Gao J.M., Striatoids A.-F. (2015). Cyathane Diterpenoids with Neurotrophic Activity from Cultures of the Fungus Cyathus striatus. J. Nat. Prod..

[9] Ohm R.A., de Jong J.F., Lugones L.G., Aerts A., Kothe E., Stajich J.E., de Vries R.P., Record E., Levasseur A., Baker S.E. (2010). Genome sequence of the model mushroom *Schizophyllum commune*. Nat. Biotechnol..

[10] Kogen H., Tago K., Kaneko S., Hamano K., Onodera K., Haruyama H., Minagawa K., Kinoshita T., Ishikawa T., Tanimoto T. (1996). Schizostatin, a novel squalene synthase inhibitor produced by the mushroom, *Schizophyllum commune*. II. Structure elucidation and total synthesis. J. Antibiot..

[11] Chunyu W.X., Ding Z.G., Zhao J.Y., Wang Y.X., Han X.L., Li M.G., Wen M.L. (2017). Two new diketopiperazines from the tin mine tailings-derived fungus *Schizophyllum commune* YIM DT 10058. Nat. Prod. Res..

[12] Chen G.G., Zhu Q.F., Long X.M., Lu Q., Li K.Y., Chen Q., Zhou M., Liao G.-S., Xu G.B. (2022). Antibacterial activities of the chemical constituents of *Schizophyllum commune* MST7-3 collected from coal area. Nat. Prod. Res..

[13] Wang C.F., Yang X.Q., Sun J., Wang T., Cui H.R., Yang Y.B. (2022). New Metabolites, Antifeedant, Insecticidal Activities, and Reciprocal Relationship Between Insect and Fungus from Endophyte *Schizophyllum commune*. Chem. Biodivers..

[14] Chen Y., Yang W., Zou G., Wang G., Kang W., Yuan J., She Z. (2022). Cytotoxic Bromine- and Iodine-Containing Cytochalasins Produced by the Mangrove Endophytic Fungus *Phomopsis* sp. QYM-13 Using the OSMAC Approach. J. Nat. Prod..

[15] Zhang Q., Wang S.Q., Tang H.Y., Li X.J., Zhang L., Xiao J., Gao Q.Y., Zhang A.L., Gao J.M. (2013). Potential allelopathic indole diketopiperazines produced by the plant endophytic Aspergillus fumigatus using the one strain-many compounds method. J. Agric. Food. Chem..

[16] Kim H., Kim J.-Y., Ji C.-h., Lee D., Shim S.H., Joo H.-S., Kang H.S. (2023). Acidonemycins A–C, Glycosylated Angucyclines with Antivirulence Activity Produced by the Acidic Culture of *Streptomyces indonesiensis*. J. Nat. Prod..

[17] Wu G., Zhou H., Zhang P., Wang X., Li W., Zhang W., Liu X., Liu H.-W., Keller N.P., An Z. (2016). Polyketide Production of Pestaloficiols and Macrodiolide Ficiolides Revealed by Manipulations of Epigenetic Regulators in an Endophytic Fungus. Org. Lett..

[18] Tang H.Y., Zhang Q., Gao Y.Q., Zhang A.L., Gao J.M. (2015). Miniolins A–C, novel isomeric furanones induced by epigenetic manipulation of *Penicillium minioluteum*. RSC. Advances..

[19] Zhang W.Y., Zhong Y., Yu Y., Shi D.F., Huang H.Y., Tang X.L., Wang Y.H., Chen G.D., Zhang H.P., Liu C.L. (2020). 4-Hydroxy Pyridones from Heterologous Expression and Cultivation of the Native Host. J. Nat. Prod..

[20] Lee S.R., Seyedsayamdost M.R. (2022). Induction of Diverse Cryptic Fungal Metabolites by Steroids and Channel Blockers. Angew Chem. Int. Ed. Engl..

[21] Smyrniotopoulos V., Merten C., Firsova D., Fearnhead H., Tasdemir D. (2020). Oxygenated Acyclic Diterpenes with Anticancer Activity from the Irish Brown Seaweed *Bifurcaria bifurcata*. Mar. Drugs.

[22] Yun B.S., Lee I.K., Woo E.E., Kim Y.J., Lee J.Y., Wo S.K., Jin K.S., Seon K.J., Ja S.S. (2018). Antimicrobial Composition Including Extract of *Schizophyllum commune* Strain Culture and Preparing Method Thereof. KR Patent.

[23] Tanimoto T., Tsujita Y., Hamano K., Haruyama H., Kinoshita T., Hosoya T., Kaneko S., Tago K., Kogen H. (1995). Schizostatin, a potent squalene synthase inhibitor from *Schizophyllum commune*: Isolation, structure elucidation, and total synthesis. Tetrahedron. Lett..

[24] Anjum K., Huang X., Zhou L., Zhu T., Che Q., Zhang G., Li D. (2023). New cyclic dipeptide discovered from deep-sea derived *Aspergillus* sp. HDN20-1401. Nat. Prod. Res..

[25] Chang Y., Xing L., Sun C., Liang S., Liu T., Zhang X., Zhu T., Pfeifer B.A., Che Q., Zhang G. (2020). Monacycliones G-K and ent-Gephyromycin A, Angucycline Derivatives from the Marine-Derived *Streptomyces* sp. HDN15129. J. Nat. Prod..

[26] Mándi A., Kurtán T. (2019). Applications of OR/ECD/VCD to the structure elucidation of natural products. Nat. Prod. Rep..

[27] Smyrniotopoulos V., Merten C., Kaiser M., Tasdemir D. (2017). Bifurcatriol, a New Antiprotozoal Acyclic Diterpene from the Brown Alga *Bifurcaria bifurcata*. Mar. Drugs.

[28] Jeong S.Y., Alishir A., Zhang S., Zhang Y., Choi S., Pang C., Bae H.Y., Jung W.H., Kim K.H. (2023). Identification of Obscurolide-Type Metabolites and Antifungal Metabolites from the Termite-Associated *Streptomyces neopeptinius* BYF101. J. Nat. Prod..

[29] Wang F., Yang J. (2012). A comparative study of caffeic acid and a novel caffeic acid conjugate SMND-309 on antioxidant properties in vitro. Lwt. Food. Sci. Technol..

[30] Wang H., Gao X.D., Zhou G.C., Cai L., Yao W.B. (2008). In vitro and in vivo antioxidant activity of aqueous extract from *Choerospondias axillaris* fruit. Food. Chem..

[31] Liu L., Chen X., Zou Y. (2022). Genome mining of fungal globin-like enzymes for catalyzing the synthesis of linear terpenes. Chin. J. Nat. Med..

