# Supplementary material for: Combining the Elicitor Up-Regulated Production of Unusual Linear Diterpene-Derived Variants for an In-Depth Assessment of the Application Value and Risk of the Medicinal and Edible Basidiomycete Schizophyllum commune
Source: Molecules. 2024 Jun 1;29(11):2608. doi: 10.3390/molecules29112608 (PMC11173764; doi:10.3390/molecules29112608)
Supplement: Supplementary file 1 [file molecules-29-02608-s001.zip › molecules-2950760-supplementary.pdf]

Supporting Information

Combining the Elicitor Up-Regulated Production of Unusual Linear Diterpene-Derived Variants for an In-Depth Assessment of the Application Value and Risk of the Medicinal and Edible Basidiomycete *Schizophyllum commune*

Ying Wang[1], Fei Cao[2], Luning Zhou[3], Hanwei Liu[4], Hua Gao[1], Ge Cui[1], Changshan Niu[5], Peng Zhang[5], Dehai Li[3], Songqi Liu[1], Yan Jiang[1,*], Guangwei Wu[1,*]

[1] College of Chemical Engineering, Nanjing Forestry University, Nanjing 210000, China

[2] Key Laboratory of Pharmaceutical Quality Control of Hebei Province, Key Laboratory of Medicinal Chemistry and Molecular Diagnostics of Education Ministry of China, College of Pharmaceutical Sciences, Hebei University, Baoding 071002, China

[3] Key Laboratory of Marine Drugs, Chinese Ministry of Education, School of Medicine and Pharmacy, Ocean University of China, 5 Yushan Road, Qingdao 266003, China.

[4] Ningbo Customs District Technology Center, Ningbo 315100, China

[5] Department of Pharmacology and Toxicology, College of Pharmacy, University of Utah, Salt Lake City, Utah 84112, USA.

* Corresponding author

E-mail addresses: gweiwu@njfu.edu.cn (G. W.); jiangyancpu@126.com (Y. J.)

Contents

Figure S1. ^{1}H NMR Spectrum of compound **1** in MeOH (600 MHz). 6
Figure S2. ^{13}C NMR spectrum of compound **1** in MeOH (150 MHz)..........................7
Figure S3. ^{1}H-^{1}H COSY spectrum of compound **1** in MeOH.....................................8
Figure S4. HMBC spectrum of compound **1** in MeOH. 9
Figure S5. HSQC spectrum of compound **1** in MeOH. 10
Figure S6. NOESY spectrum of compound **1** in MeOH..11
Figure S7. IR spectrum of compound **1** in MeOH. 12
Figure S8. HR-ESI-MS spectrum of compound **1** in MeOH. 13
Figure S9. Correlation plots of experimental ^{13}C NMR chemical shifts versus the corresponding calculated data for (9*R**, 14*S**)−**1** and (9*S**, 14*S**)−**1**. 14
Figure S10. sDP4+, uDP4+ and DP4+ probabilities (%) for (9*R**, 14*S**)−**1** (Isomer 2) and (9*S**, 14*S**)−**1** (Isomer 1). 15
Figure S11. ^{1}H NMR spectrum of compound **2** in MeOH (600 MHz). 16
Figure S12. ^{13}C NMR spectrum of compound **2** in MeOH (150 MHz).....................17
Figure S13. ^{1}H-^{1}H COSY spectrum of compound **2** in MeOH.................................18
Figure S14. HMBC spectrum of compound **2** in MeOH. 19
Figure S15. HSQC spectrum of compound **2** in MeOH. 20
Figure S16. NOESY spectrum of compound **2** in MeOH..21
Figure S17. IR spectrum of compound **2** in MeOH. 22
Figure S18. HR-ESI-MS spectrum of compound **2** in MeOH. 23
Figure S19. ^{1}H NMR spectrum of compound **3** in MeOH (600 MHz).......................24
Figure S20. ^{13}C NMR spectrum of compound **3** in MeOH (150 MHz).....................25
Figure S21. ^{1}H-^{1}H COSY spectrum of compound **3** in MeOH.................................26
Figure S22. HMBC spectrum of compound **3** in MeOH. 27
Figure S23. HSQC spectrum of compound **3** in MeOH. 28
Figure S24. NOESY spectrum of compound **3** in MeOH..29
Figure S25. IR spectrum of compound **3** in MeOH. 30

Figure S26. HR-ESI-MS spectrum of compound **3** in MeOH. 31
Figure S27. ^{1}H NMR spectrum of compound **4** in MeOH (600 MHz). 32
Figure S28. ^{13}C NMR spectrum of compound **4** in MeOH (150 MHz). 33
Figure S29. ^{1}H-^{1}H COSY spectrum of compound **4** in MeOH. 34
Figure S30. HMBC spectrum of compound **4** in MeOH. 35
Figure S31. HSQC spectrum of compound **4** in MeOH. 36
Figure S32. NOESY spectrum of compound **4** in MeOH. 37
Figure S33. IR spectrum of compound **4** in MeOH. 38
Figure S34. HR-ESI-MS spectrum of compound **4** in MeOH. 39
Figure S35. ^{1}H NMR spectrum of compound **5** in MeOH (600 MHz). 40
Figure S36. ^{13}C NMR spectrum of compound **5** in MeOH (150 MHz). 41
Figure S37. ^{1}H-^{1}H COSY spectrum of compound **5** in MeOH (150 MHz). 42
Figure S38. HMBC spectrum of compound **5** in MeOH. 43
Figure S39. HSQC spectrum of compound **5** in MeOH. 44
Figure S40. NOESY spectrum of compound **5** in MeOH. 45
Figure S41. IR spectrum of compound **5** in MeOH. 46
Figure S42. HR-ESI-MS spectrum of compound **5** in MeOH. 47
Figure S43. ^{1}H NMR spectrum of compound **6** in MeOH (600 MHz). 48
Figure S44. ^{13}C NMR spectrum of compound **6** in MeOH (150 MHz). 49
Figure S45. ^{1}H-^{1}H COSY spectrum of compound **6** in MeOH. 50
Figure S46. HMBC spectrum of compound **6** in MeOH. 51
Figure S47. HSQC spectrum of compound **6** in MeOH. 52
Figure S48. NOESY spectrum of compound **6** in MeOH. 53
Figure S49. IR spectrum of compound **6** in MeOH. 54
Figure S50. HR-ESI-MS spectrum of compound **6** in MeOH. 55
Figure S51. ^{1}H NMR spectrum of compound **7** in MeOH (600 MHz). 56
Figure S52. ^{13}C NMR spectrum of compound **7** in MeOH (150 MHz). 57
Figure S53. ^{1}H-^{1}H COSY spectrum of compound **7** in MeOH. 58
Figure S54. HMBC spectrum of compound **7** in MeOH. 59

Figure S55. HSQC spectrum of compound **7** in MeOH. 60

Figure S56. NOESY spectrum of compound **7** in MeOH. 61

Figure S57. IR spectrum of compound **7** in MeOH. 62

Figure S58. HR-ESI-MS spectrum of compound **7** in MeOH. 63

Figure S59. ^{1}H NMR spectrum of compound **8** in MeOH (600 MHz). 64

Figure S60. ^{13}C NMR spectrum of compound **8** in MeOH (150 MHz). 65

Figure S61. ^{1}H-^{1}H COSY spectrum of compound **8** in MeOH. 66

Figure S62. HMBC spectrum of compound **8** in MeOH. 67

Figure S63. HSQC spectrum of compound **8** in MeOH. 68

Figure S64. NOESY spectrum of compound **8** in MeOH. 69

Figure S65. IR spectrum of compound **8** in MeOH. 70

Figure S66. HR-ESI-MS spectrum of compound **8** in MeOH. 71

Figure S67. ^{1}H NMR spectrum of compound **9** in MeOH (600 MHz). 72

Figure S68. ^{13}C NMR spectrum of compound **9** in MeOH (150 MHz). 73

Figure S69. ^{1}H-^{1}H COSY spectrum of compound **9** in MeOH. 74

Figure S70. HMBC spectrum of compound **9** in MeOH. 75

Figure S71. HSQC spectrum of compound **9** in MeOH. 76

Figure S72. NOESY spectrum of compound **9** in MeOH. 77

Figure S73. IR spectrum of compound **9** in MeOH. 78

Figure S74. HR-ESI-MS spectrum of compound **9** in MeOH. 79

Figure S75. ^{1}H NMR spectrum of compound **10** in MeOH (600 MHz). 80

Figure S76. ^{13}C NMR spectrum of compound **10** in MeOH (150 MHz). 81

Figure S77. ^{1}H-^{1}H COSY spectrum of compound **10** in MeOH. 82

Figure S78. HMBC spectrum of compound **10** in MeOH. 83

Figure S79. HSQC spectrum of compound **10** in MeOH. 84

Figure S80. NOESY spectrum of compound **10** in MeOH. 85

Figure S81. IR spectrum of compound **10** in MeOH. 86

Figure S82. HR-ESI-MS spectrum of compound **10** in MeOH. 87

Figure S83. ^{1}H NMR spectrum of compound **11** in MeOH (600 MHz). 88

Figure S84. ^{13}C NMR spectrum of compound **11** in MeOH (150 MHz). 89

Figure S85. ^{1}H-^{1}H COSY spectrum of compound **11** in MeOH. 90

Figure S86. HMBC spectrum of compound **11** in MeOH. 91

Figure S87. HSQC spectrum of compound **11** in MeOH. 92

Figure S88. NOESY spectrum of compound **11** in MeOH. 93

Figure S89. IR spectrum of compound **11** in MeOH. 94

Figure S90. HR-ESI-MS spectrum of compound **11** in MeOH. 95

Figure S91. ^{1}H NMR spectrum of compound **12** in MeOH (600 MHz). 96

Figure S92. ^{1}H NMR spectrum of compound **13** in MeOH (600 MHz). 97

Figure S93. ^{1}H NMR spectrum of compound **14** in MeOH (600 MHz). 98

ITS sequences of *Schizophyllum commune* NJFU21 99

Table S1. The coordinate for the lowest-energy conformer compound **4**-*S* in VCD calculations. 100

Table S2. The coordinate for the lowest-energy conformer compound **4**-*R* in VCD calculations. 102

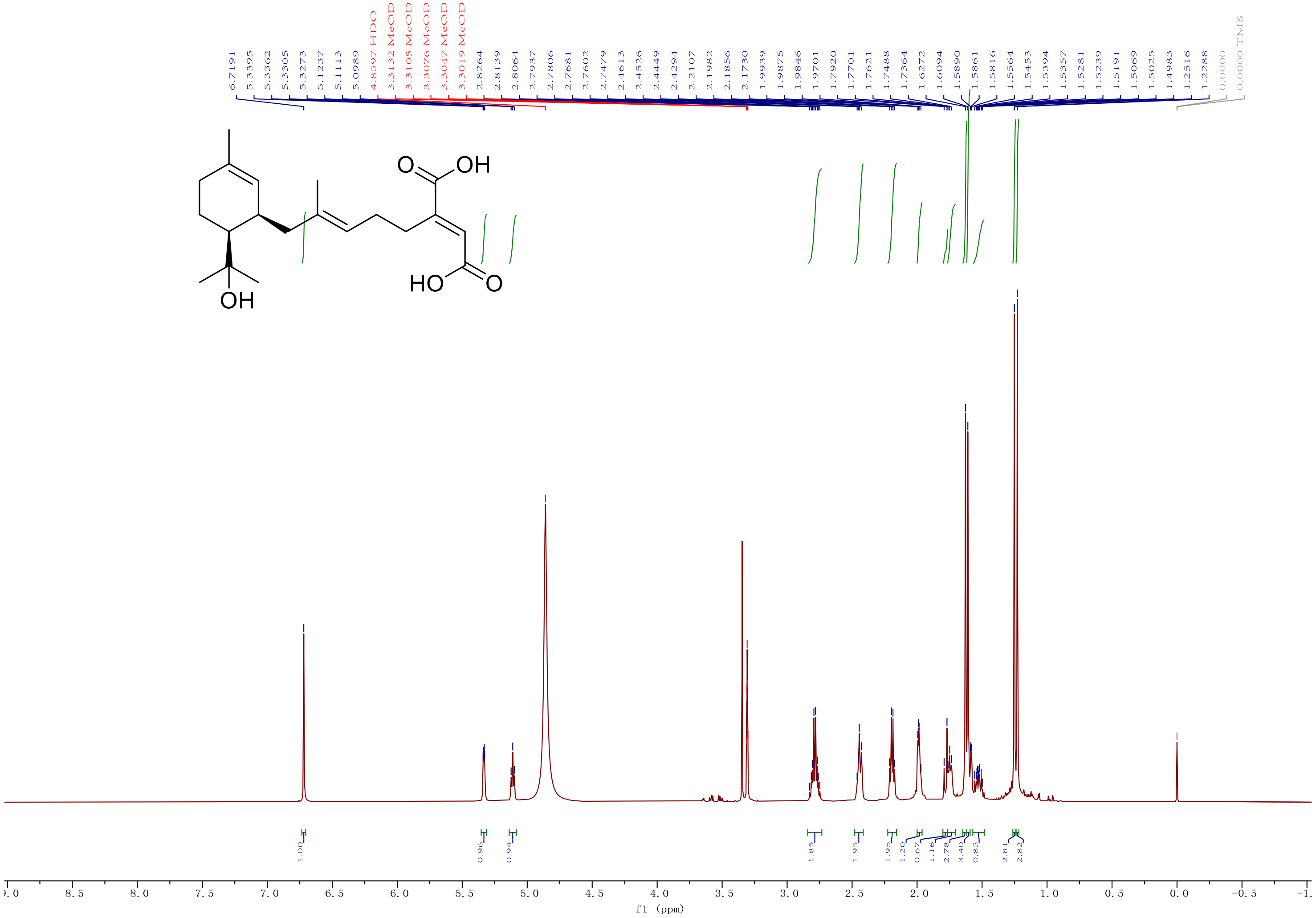

Figure S1. ^{1}H NMR Spectrum of compound **1** in MeOH (600 MHz).

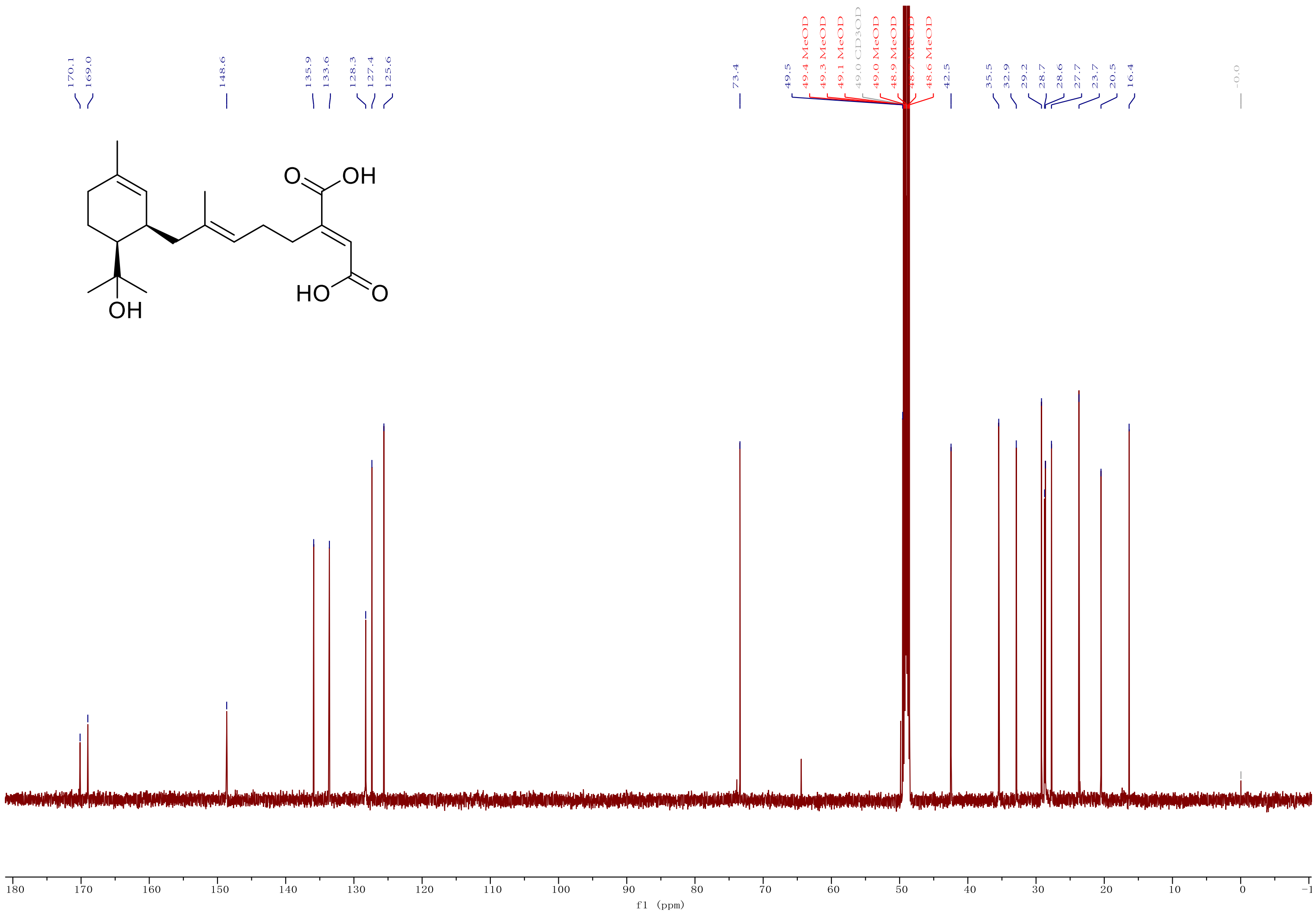

Figure S2. ^{13}C NMR spectrum of compound **1** in MeOH (150 MHz).

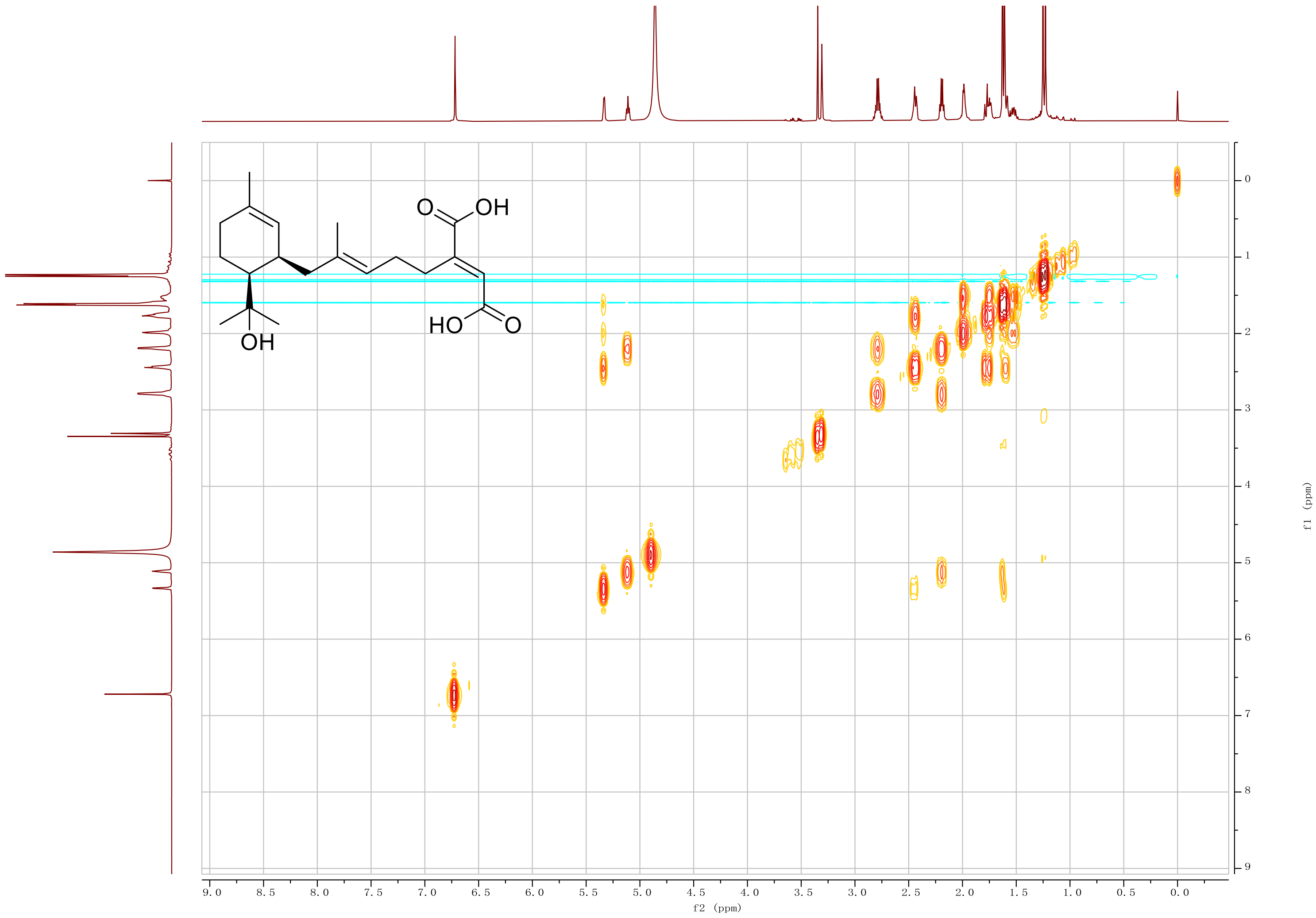

Figure S3. ¹H-¹H COSY spectrum of compound **1** in MeOH.

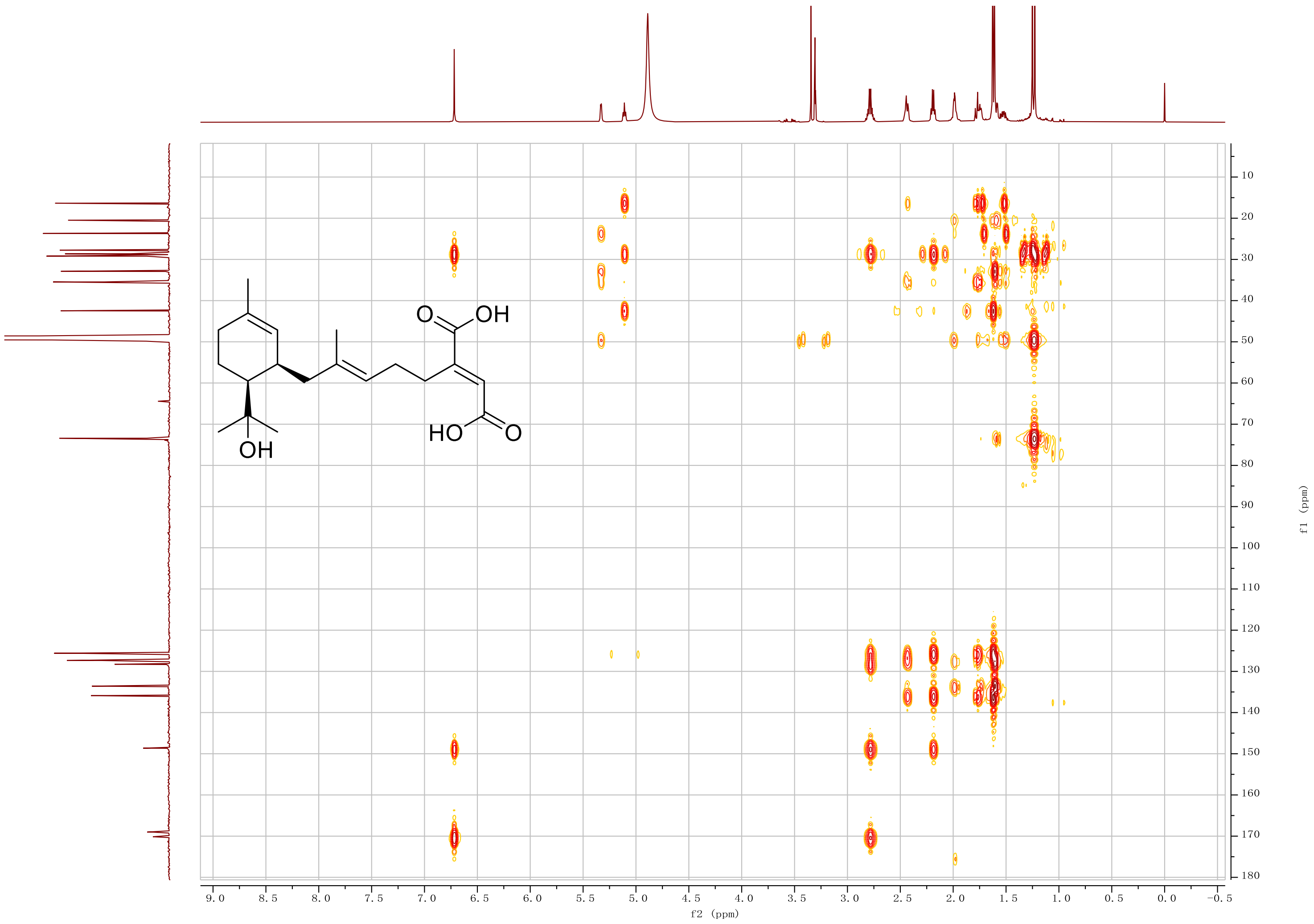

Figure S4. HMBC spectrum of compound **1** in MeOH.

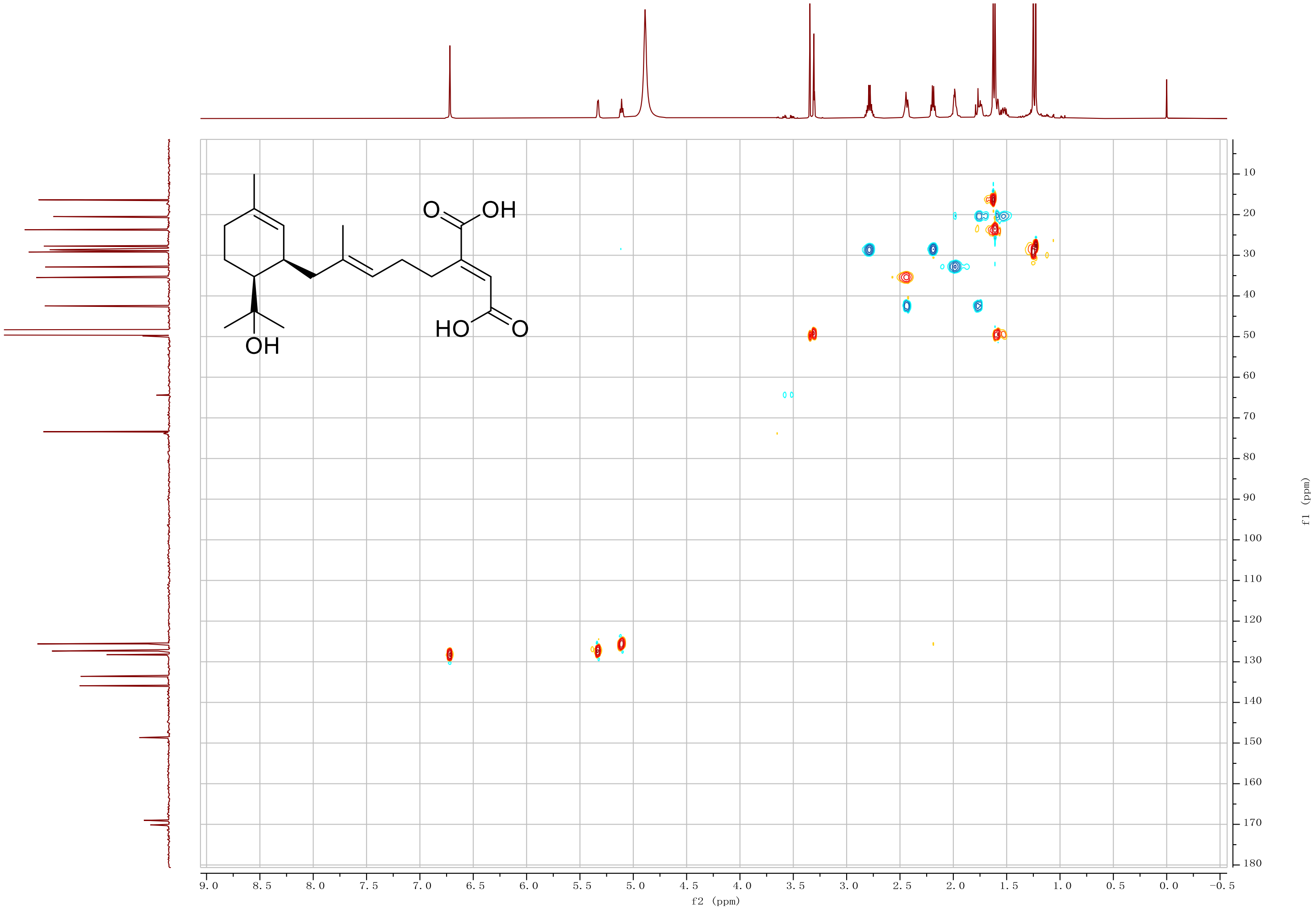

Figure S5. HSQC spectrum of compound **1** in MeOH.

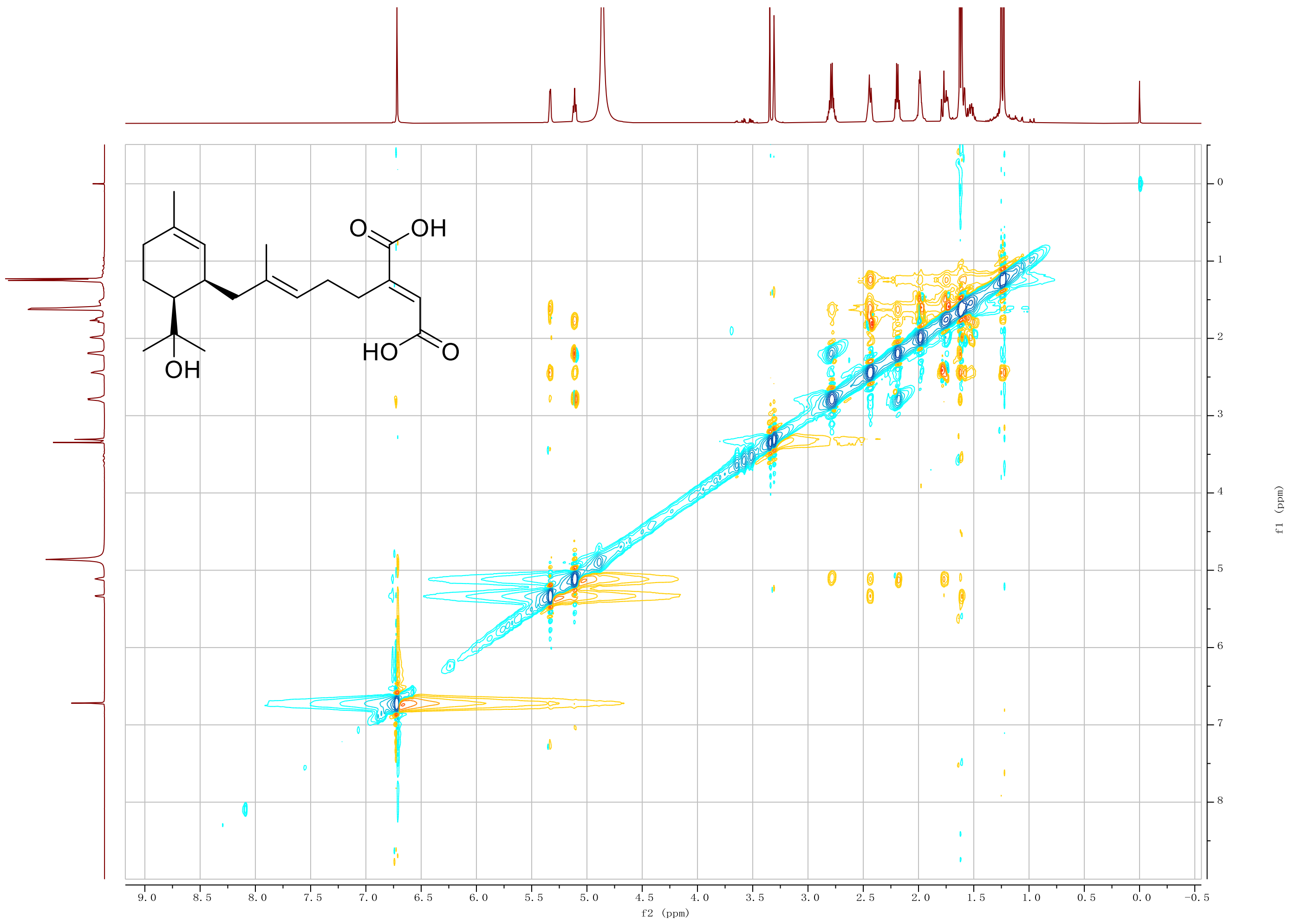

Figure S6. NOESY spectrum of compound **1** in MeOH.

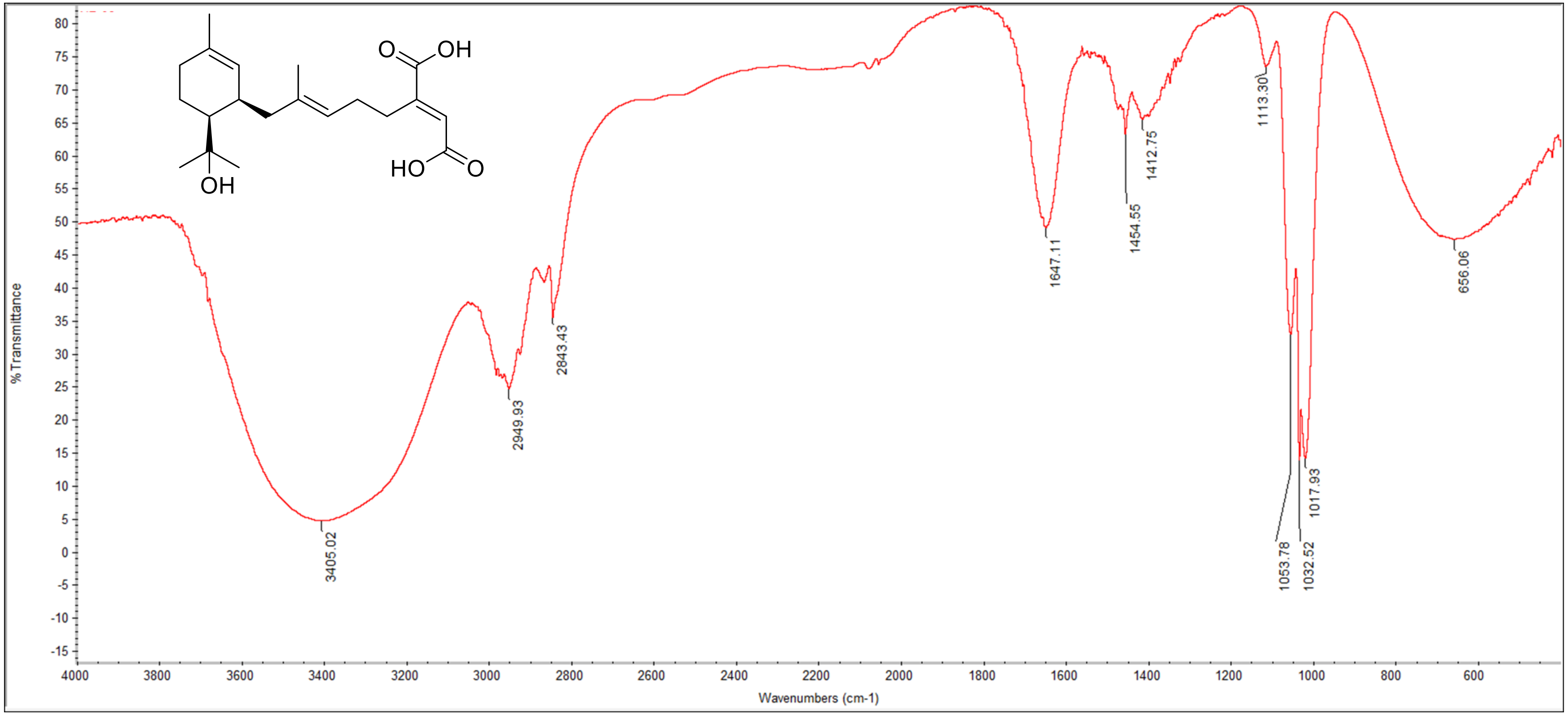

Figure S7. IR spectrum of compound **1** in MeOH.

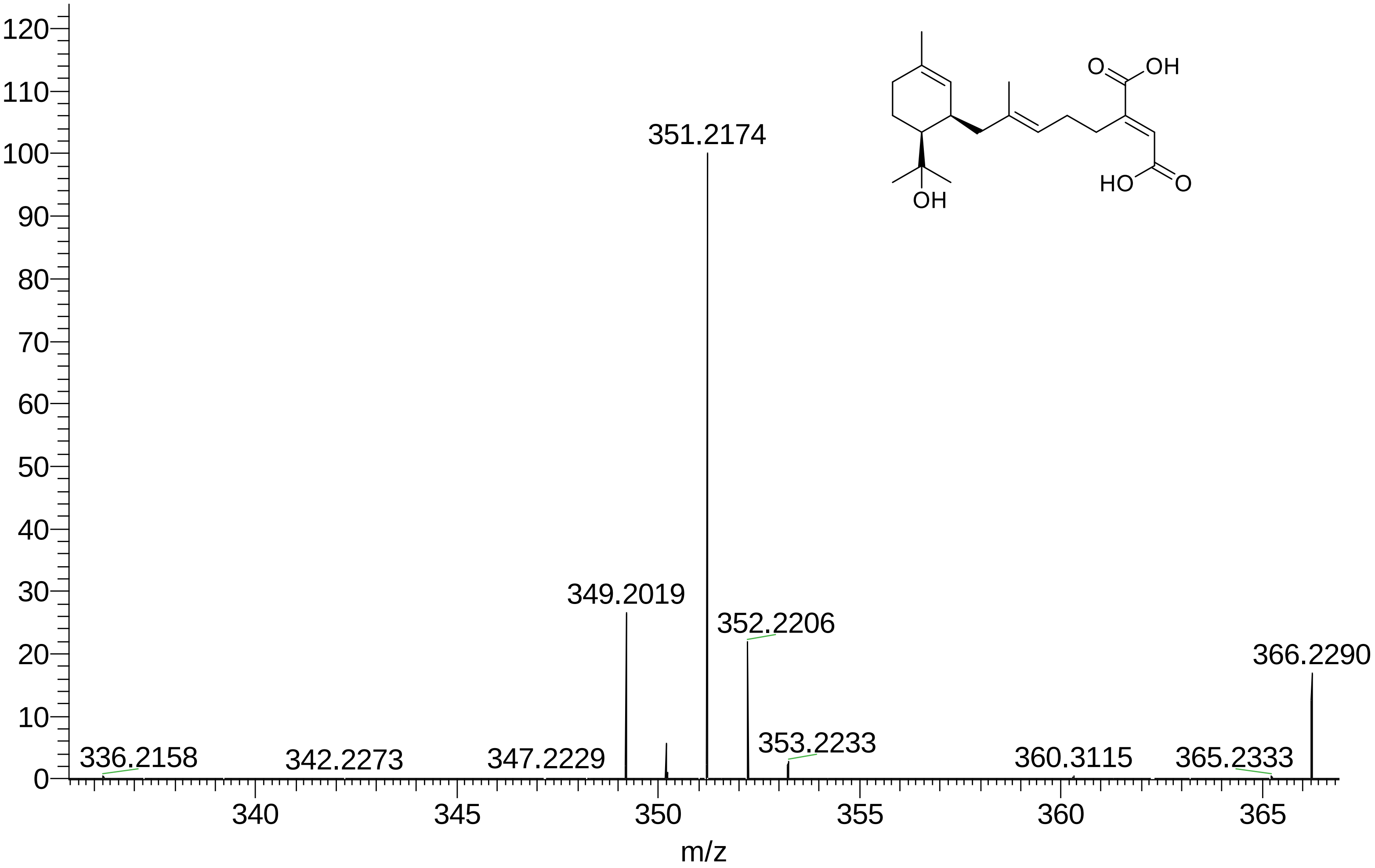

Figure S8. HR-ESI-MS spectrum of compound **1** in MeOH.

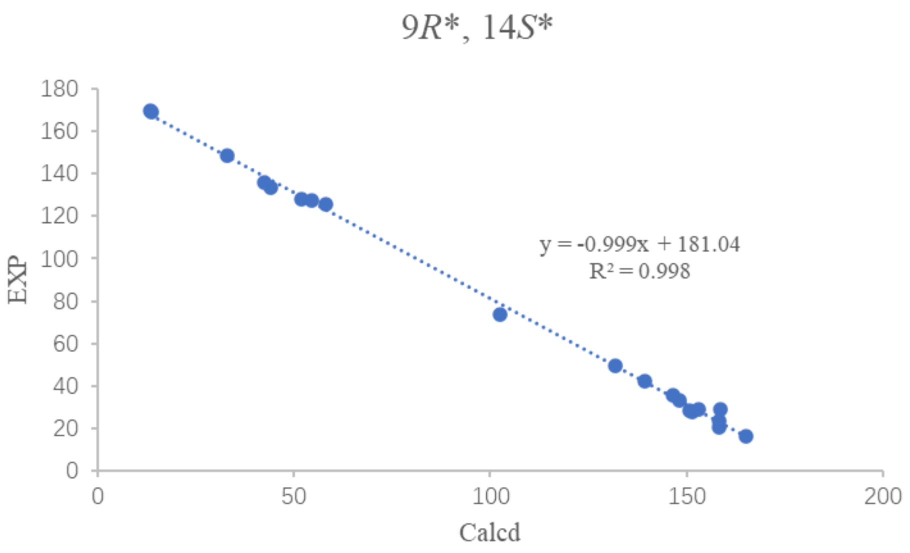

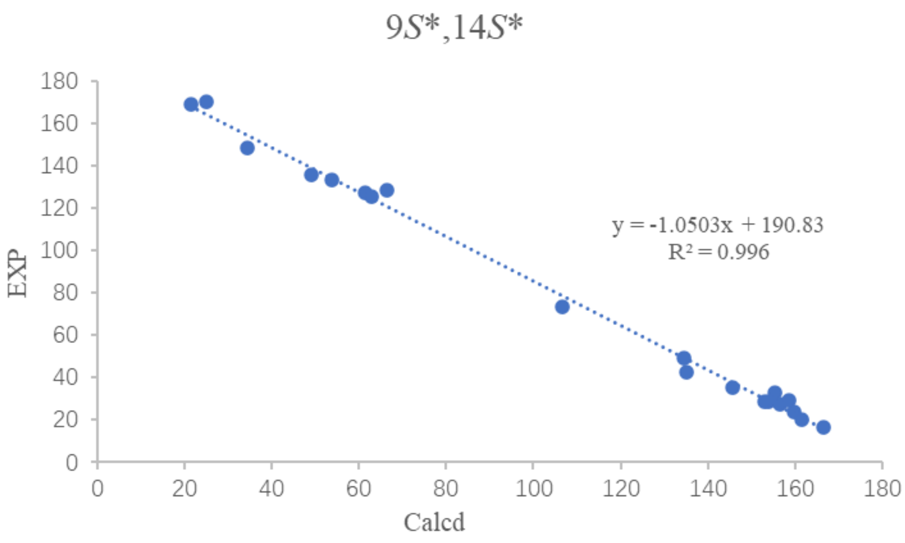

Figure S9. Correlation plots of experimental ^{13}C NMR chemical shifts versus the corresponding calculated data for (9*R**, 14*S**)−**1** and (9*S**, 14*S**)−**1**.

Functional	Solvent?	Basis Set	Type of Data
B3LYP	PCM	6-311+G(d,p)	Shielding Tensors

	Isomer 1	Isomer 2	Isomer 3	Isomer 4	Isomer 5	Isomer 6
sDP4+ (H data)	0.05%	99.95%	–	–	–	–
sDP4+ (C data)	0.00%	100.00%	–	–	–	–
sDP4+ (all data)	0.00%	100.00%	–	–	–	–
uDP4+ (H data)	0.05%	99.95%	–	–	–	–
uDP4+ (C data)	0.00%	100.00%	–	–	–	–
uDP4+ (all data)	0.00%	100.00%	–	–	–	–
DP4+ (H data)	0.00%	100.00%	–	–	–	–
DP4+ (C data)	0.00%	100.00%	–	–	–	–
DP4+ (all data)	0.00%	100.00%	–	–	–	–

Figure S10. sDP4+, uDP4+ and DP4+ probabilities (%) for (9*R**, 14*S**)−**1** (Isomer 2) and (9*S**, 14*S**)−**1** (Isomer 1).

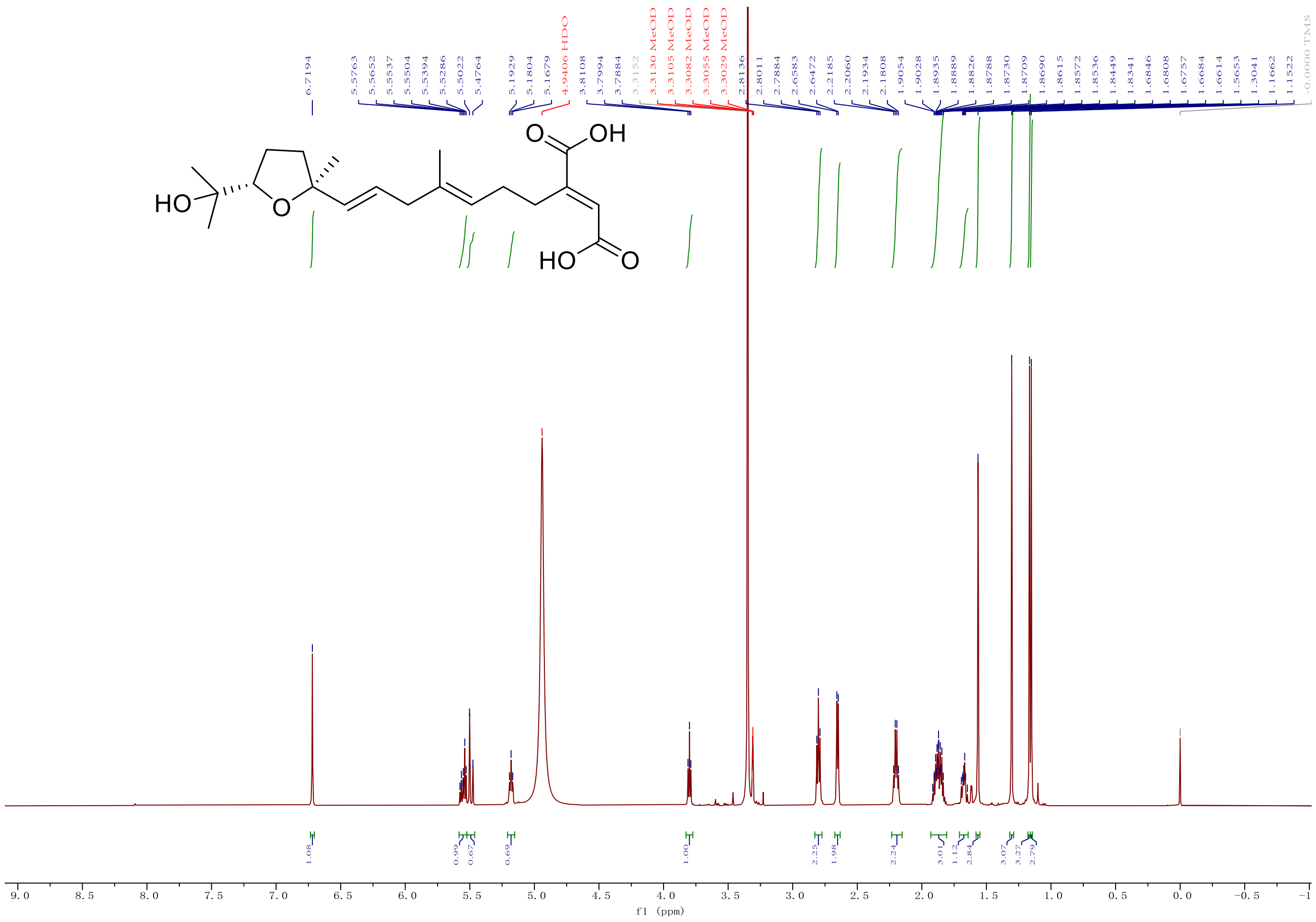

Figure S11. ^{1}H NMR spectrum of compound **2** in MeOH (600 MHz).

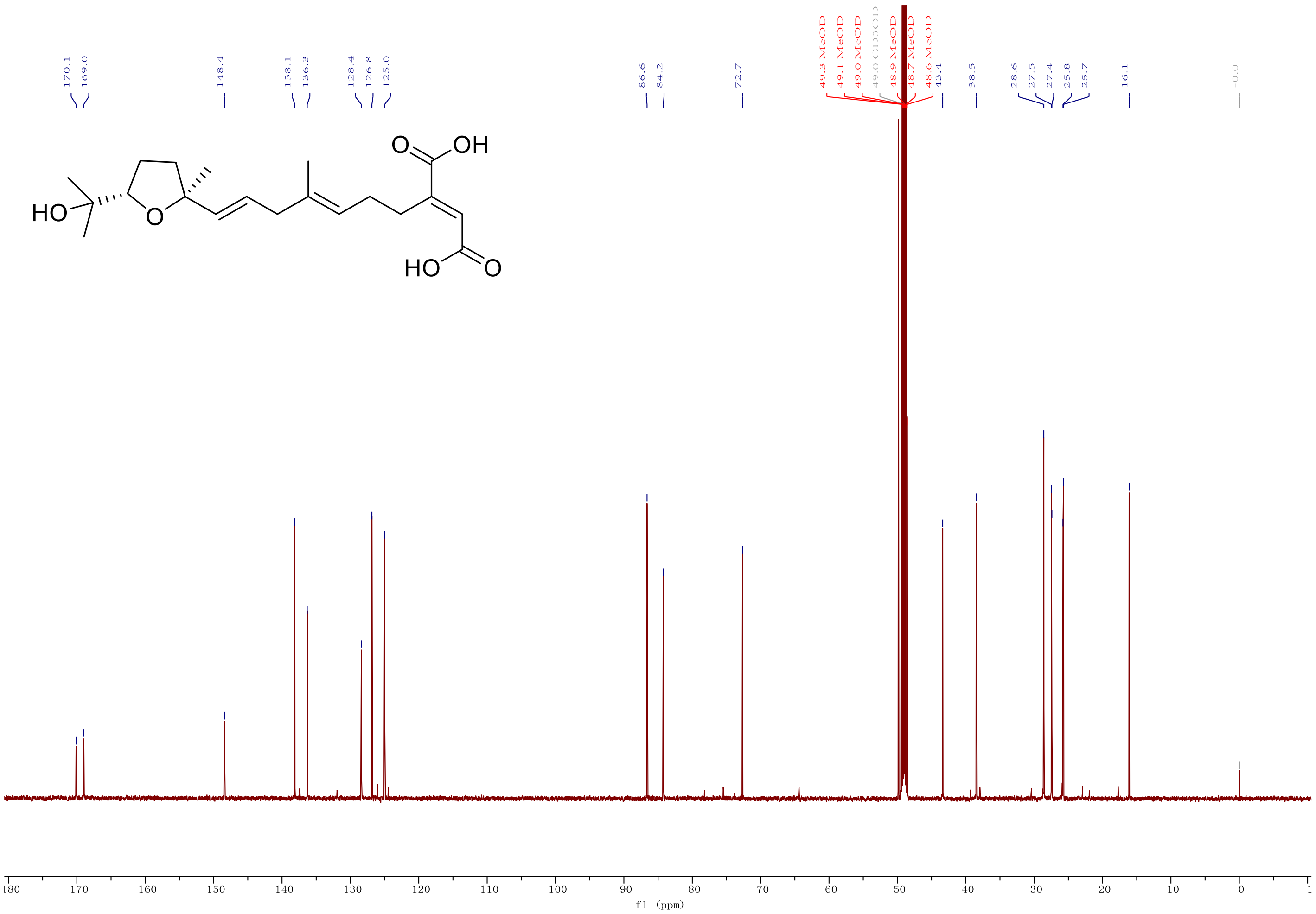

Figure S12. ^{13}C NMR spectrum of compound **2** in MeOH (150 MHz).

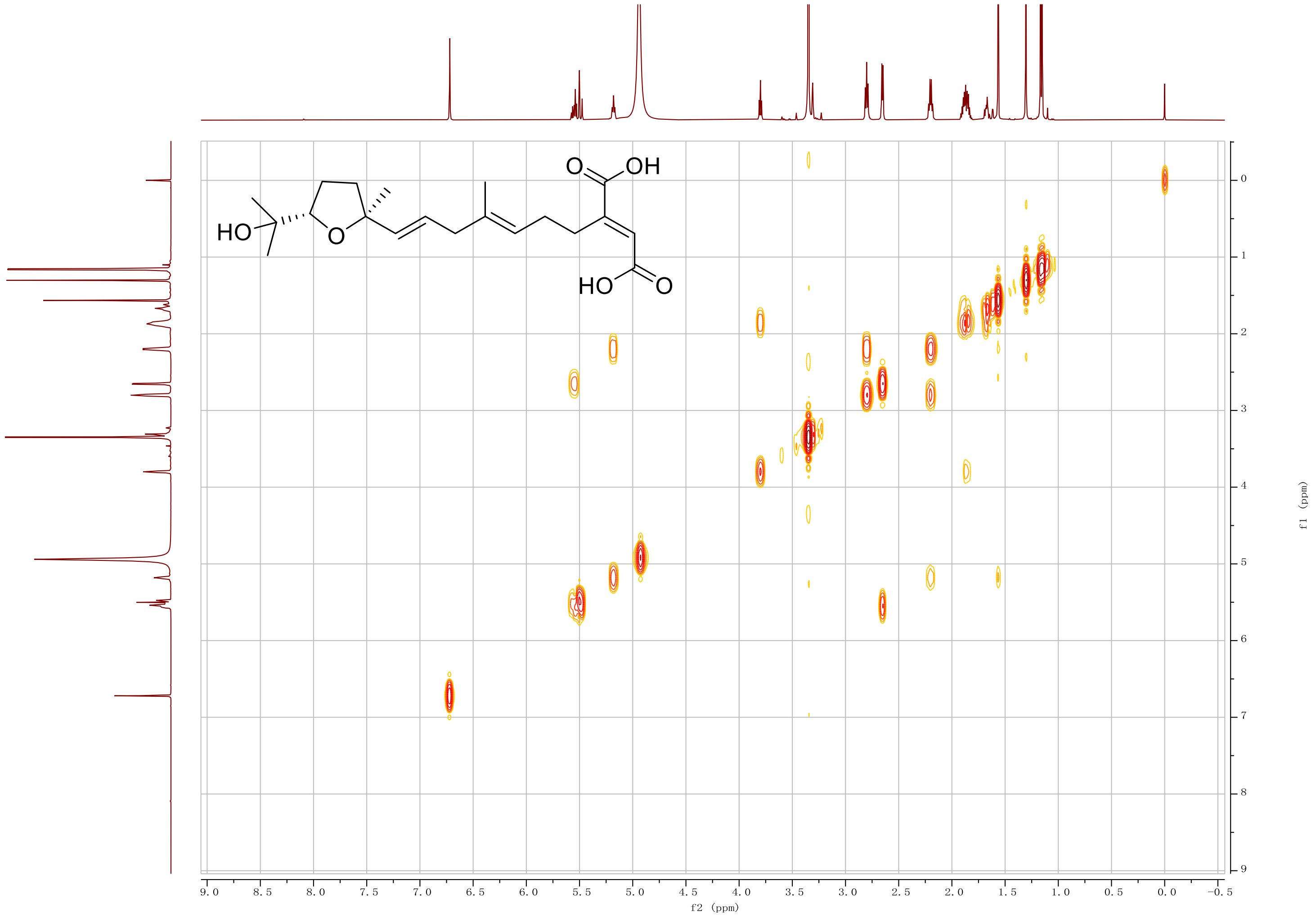

Figure S13. ^{1}H-^{1}H COSY spectrum of compound **2** in MeOH.

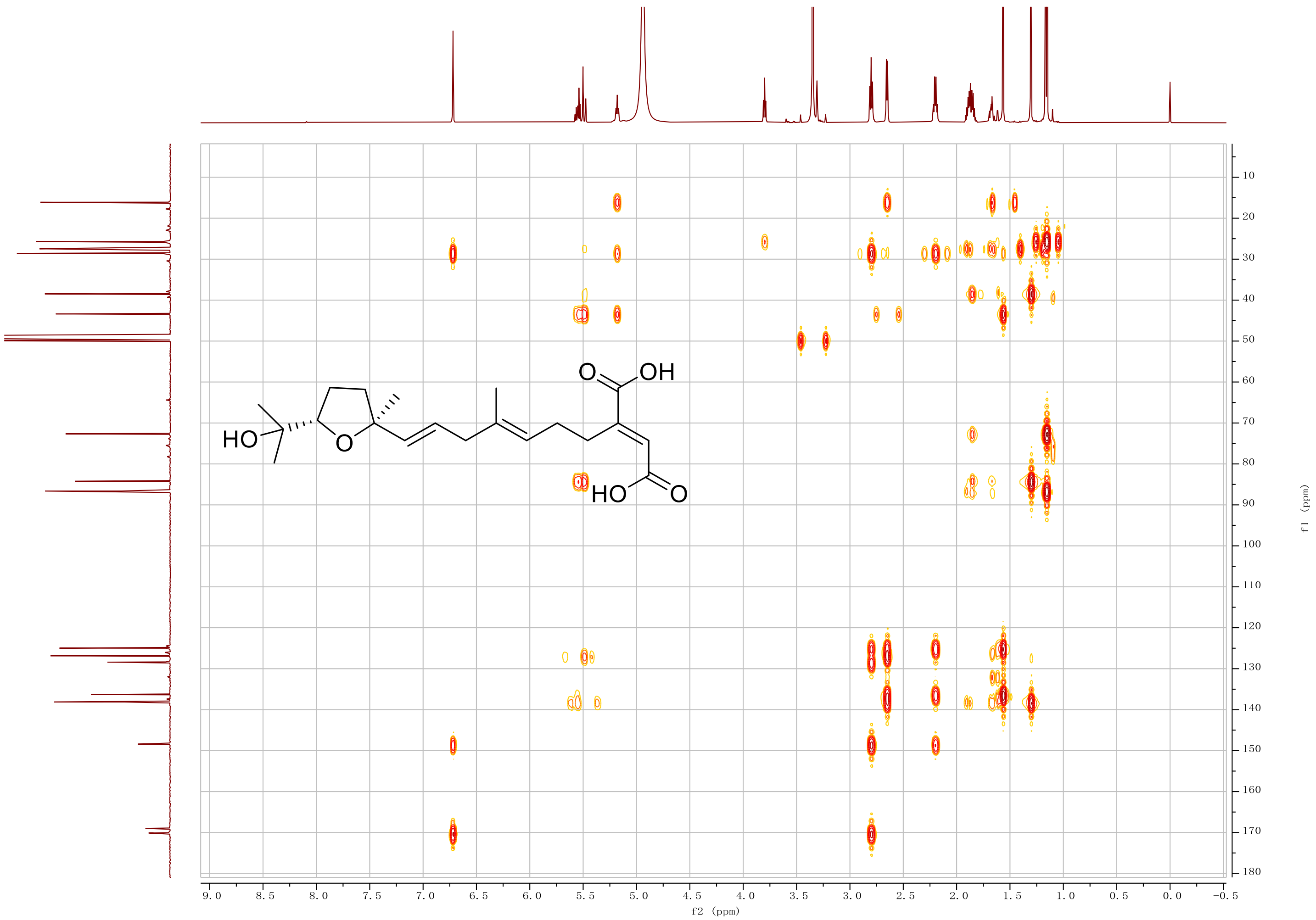

Figure S14. HMBC spectrum of compound **2** in MeOH.

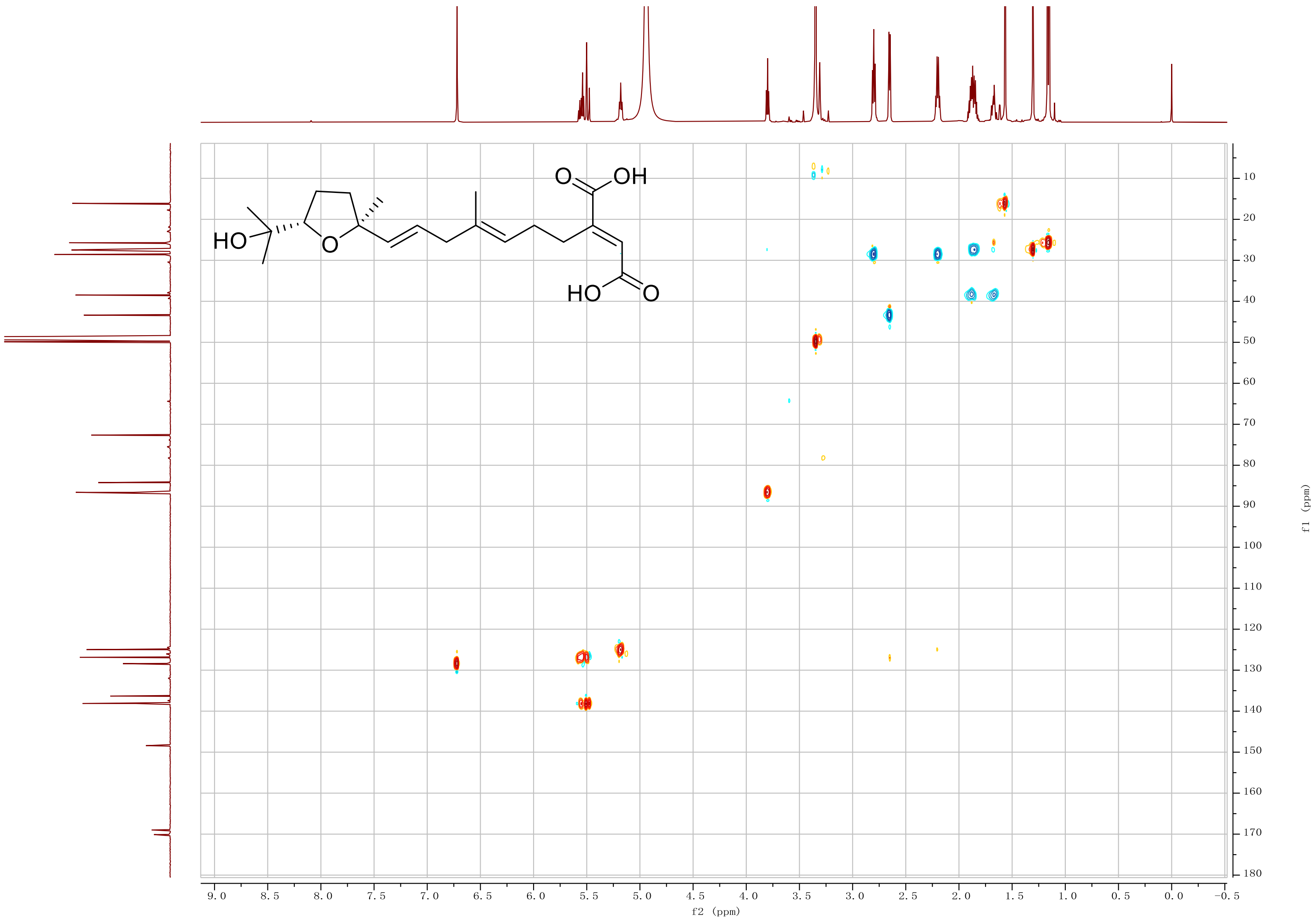

Figure S15. HSQC spectrum of compound **2** in MeOH.

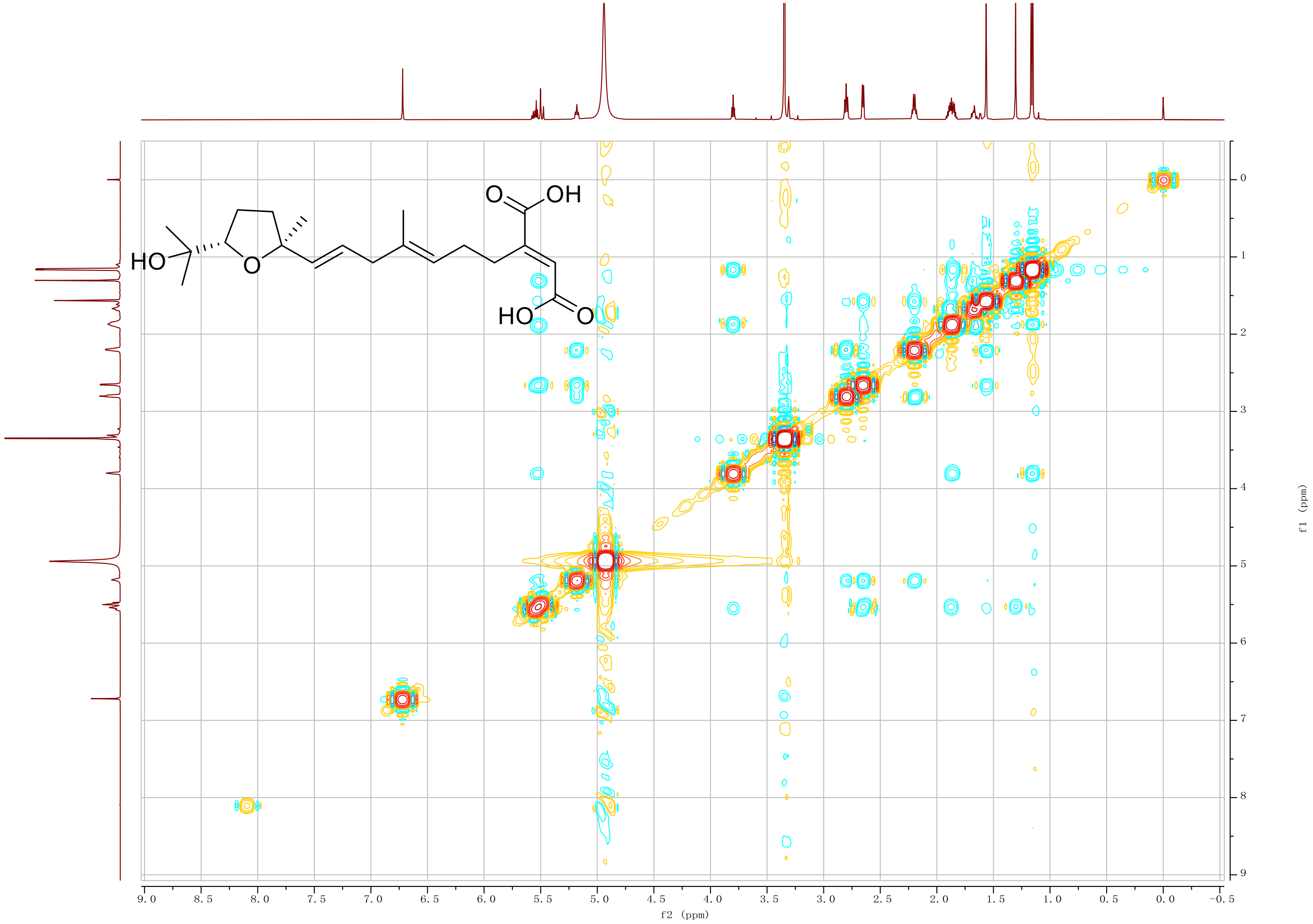

Figure S16. NOESY spectrum of compound **2** in MeOH.

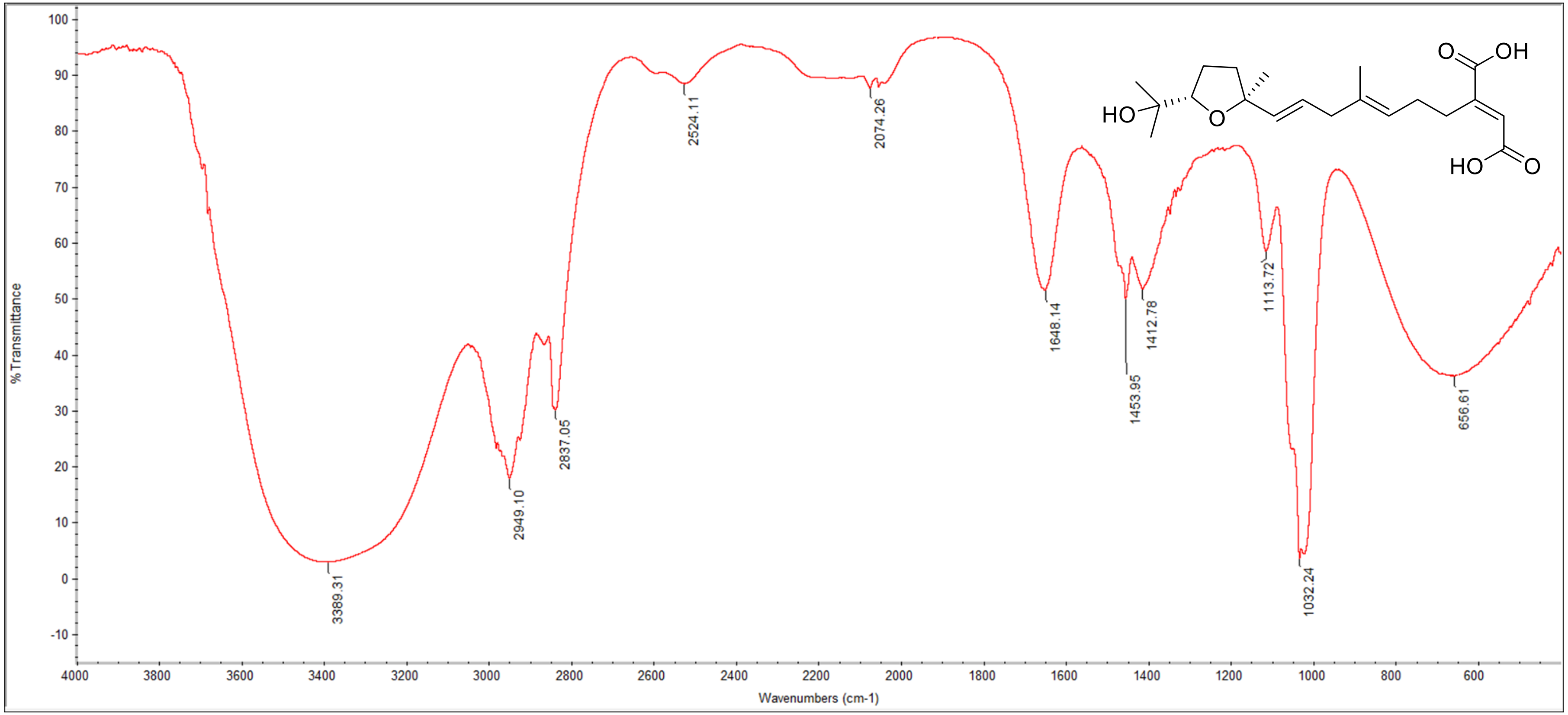

Figure S17. IR spectrum of compound **2** in MeOH.

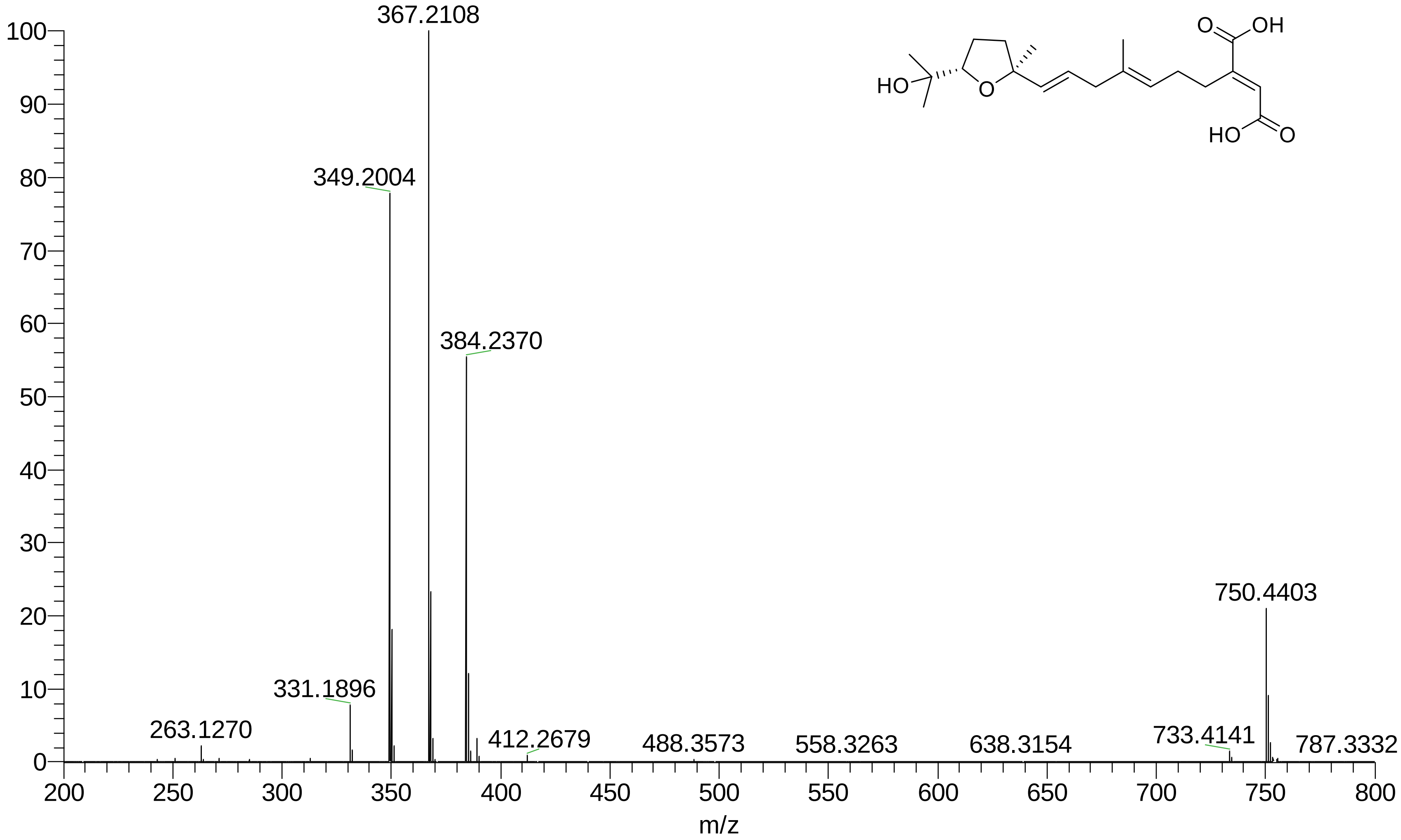

Figure S18. HR-ESI-MS spectrum of compound **2** in MeOH.

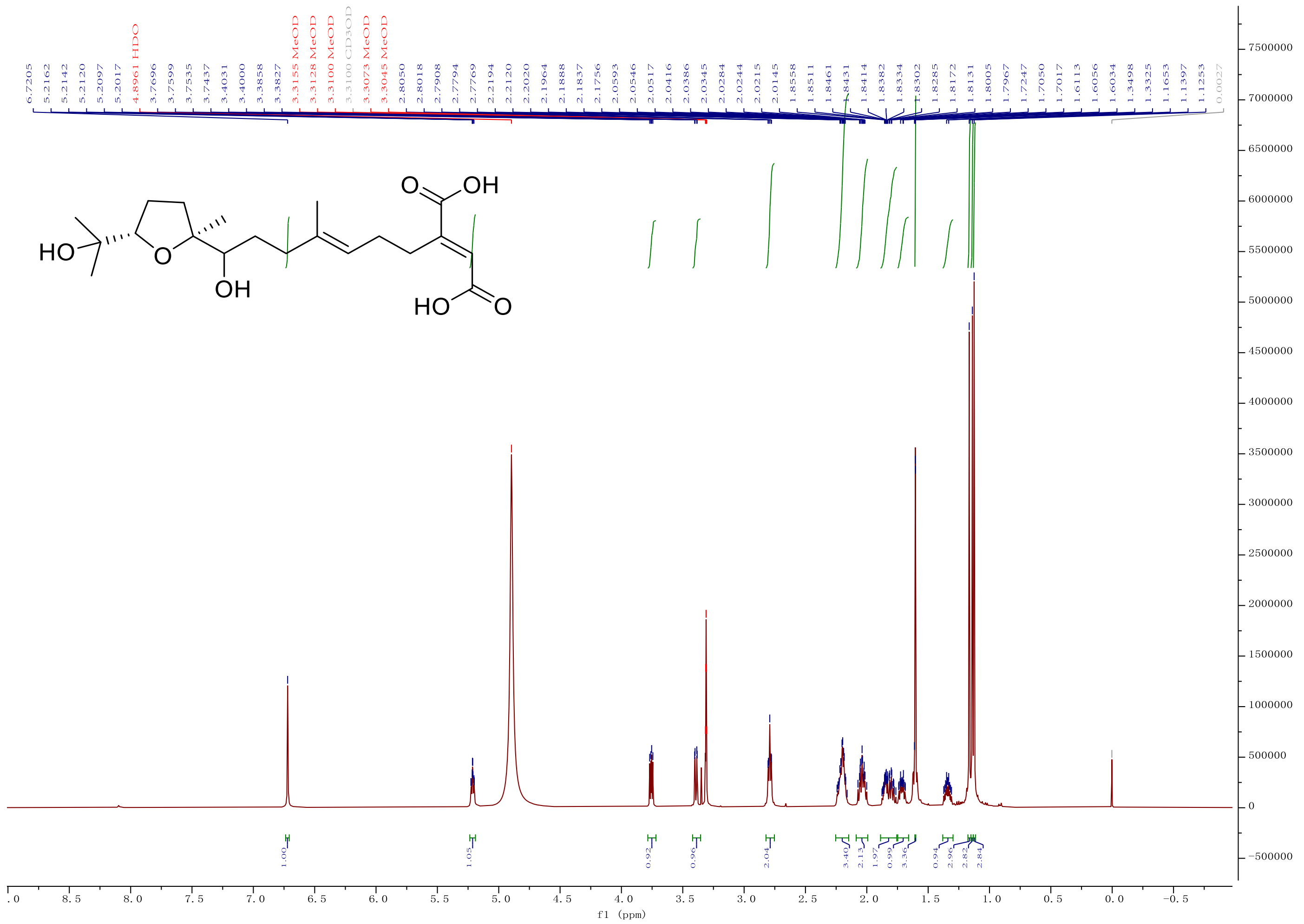

Figure S19. ^{1}H NMR spectrum of compound **3** in MeOH (600 MHz).

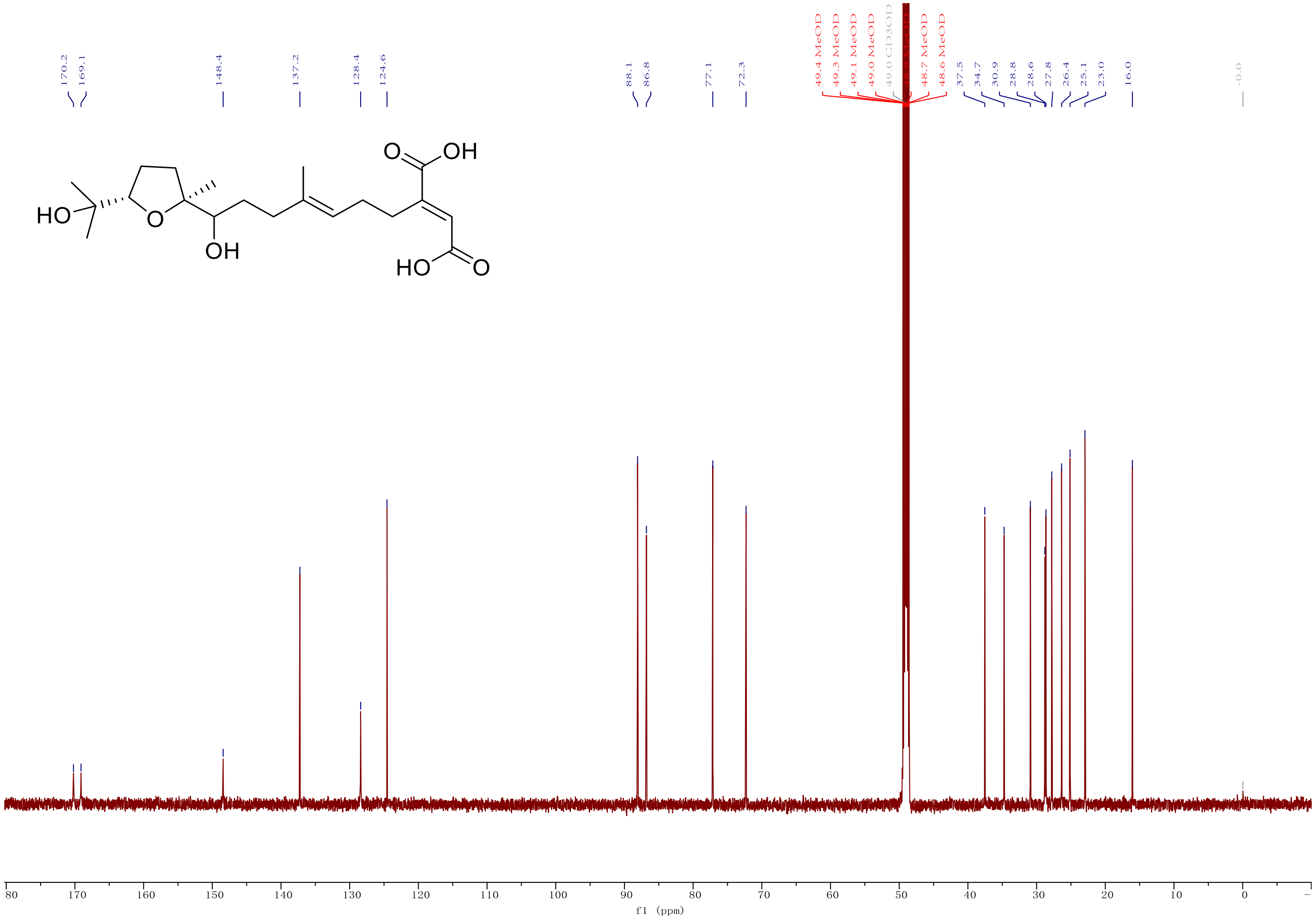

Figure S20. ^{13}C NMR spectrum of compound **3** in MeOH (150 MHz).

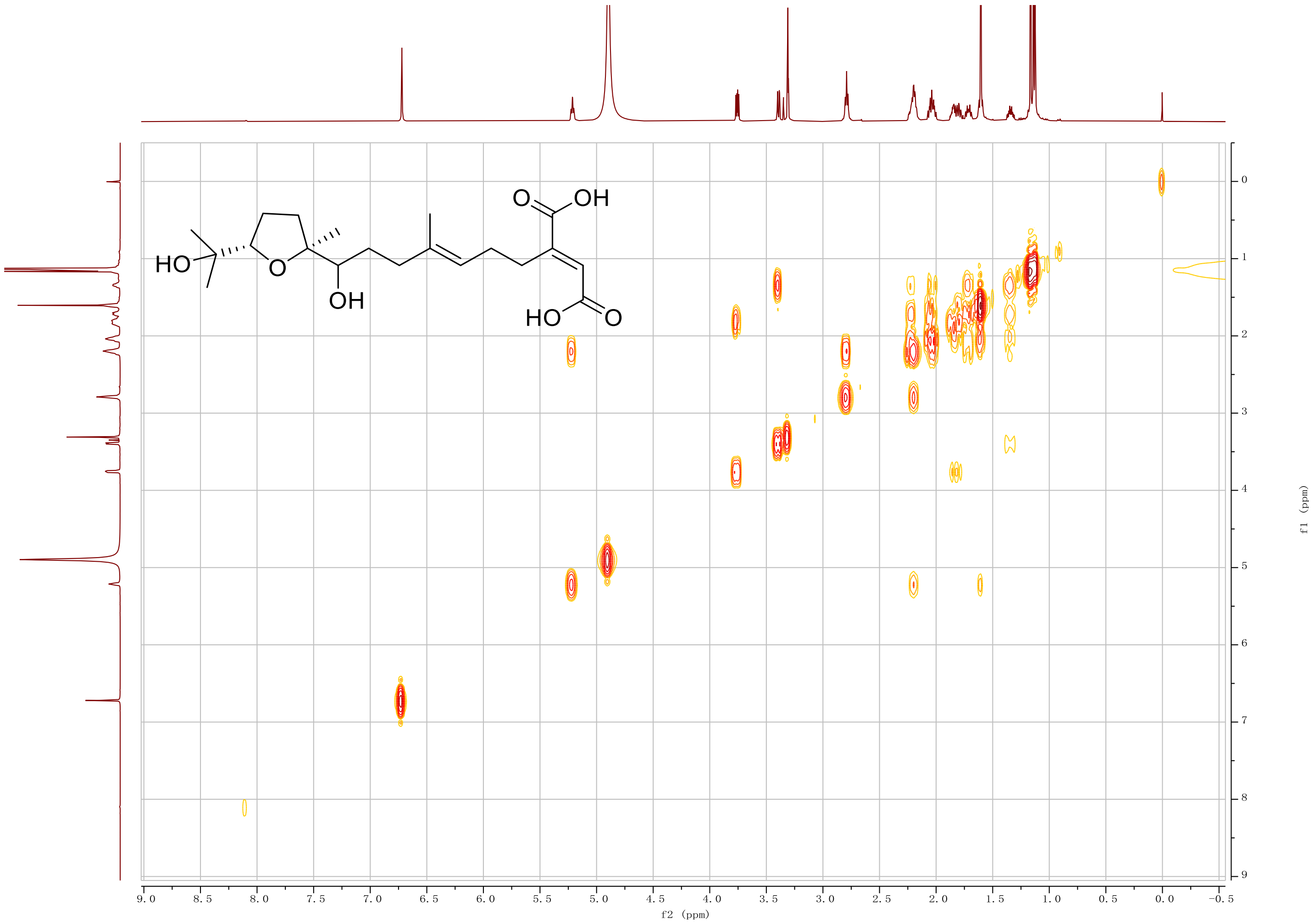

Figure S21. ^{1}H-^{1}H COSY spectrum of compound **3** in MeOH.

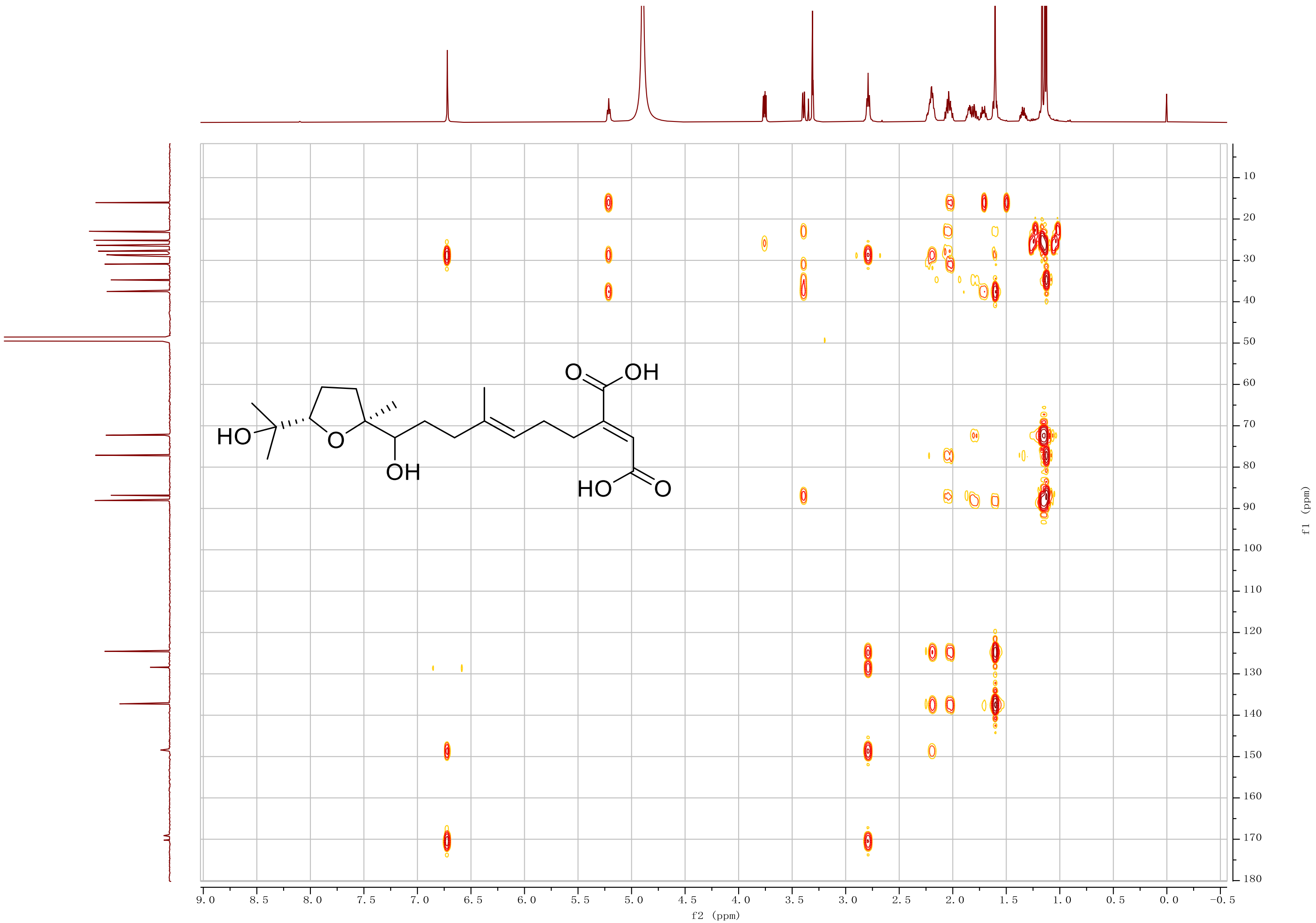

Figure S22. HMBC spectrum of compound **3** in MeOH.

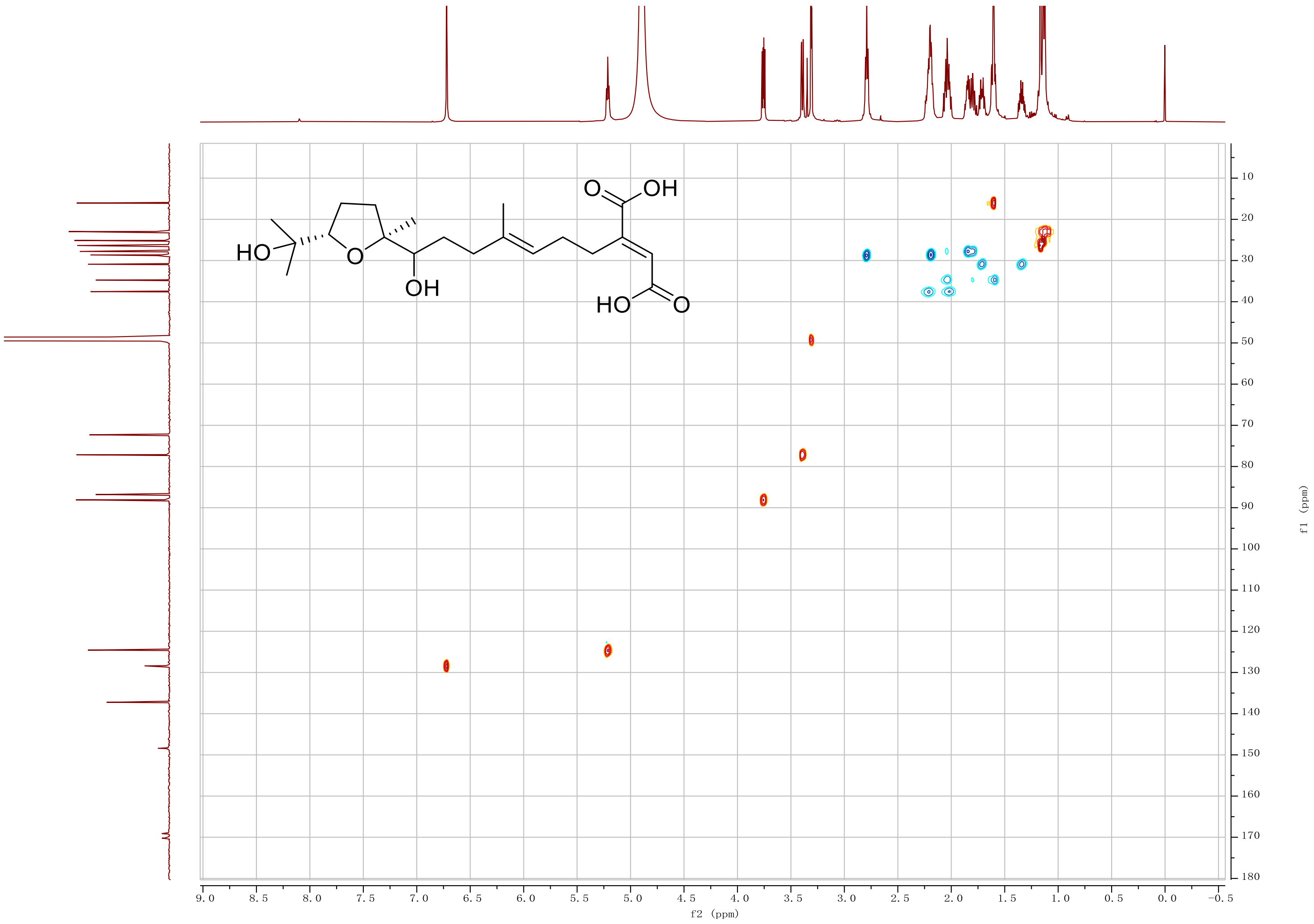

Figure S23. HSQC spectrum of compound **3** in MeOH.

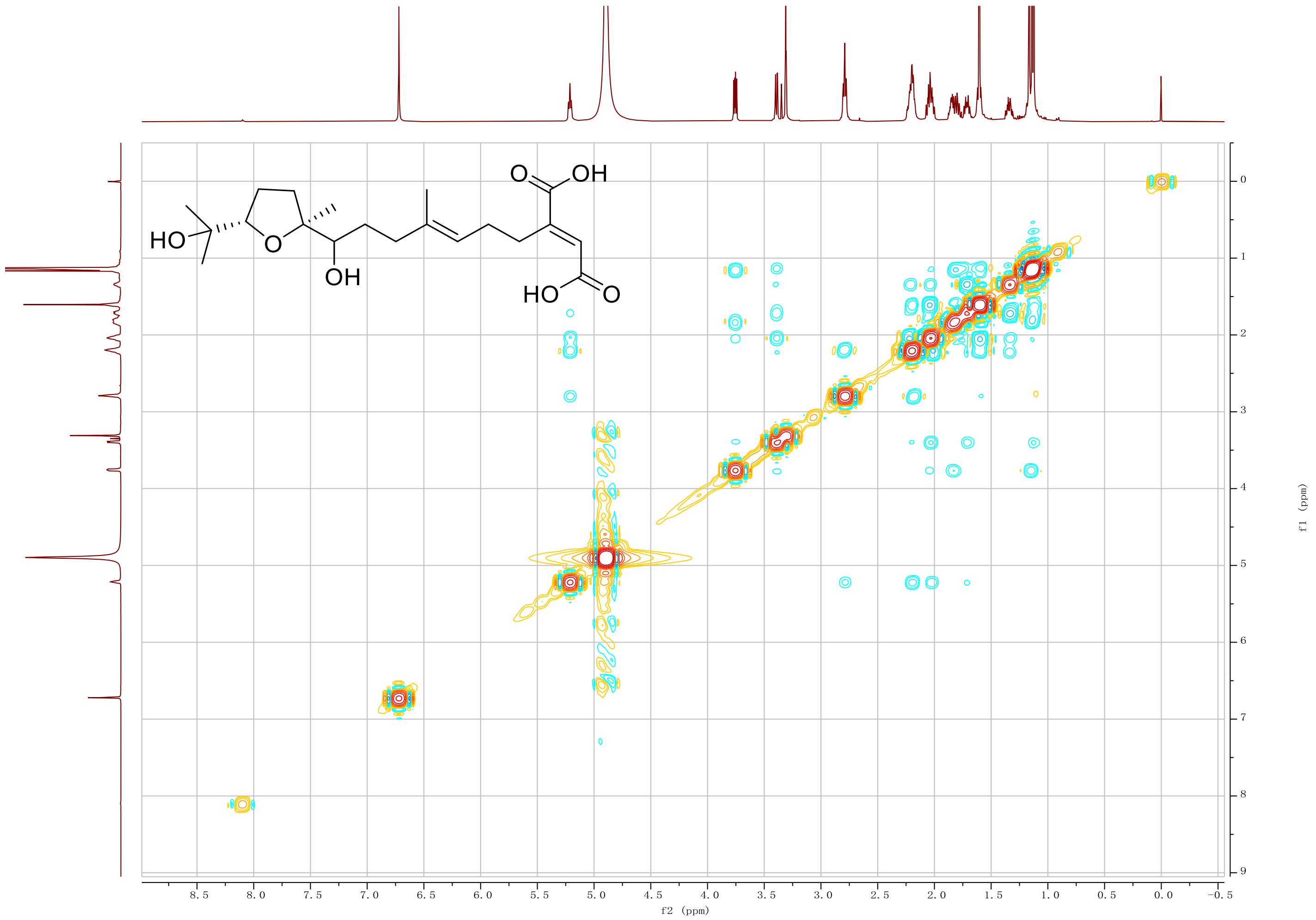

Figure S24. NOESY spectrum of compound **3** in MeOH.

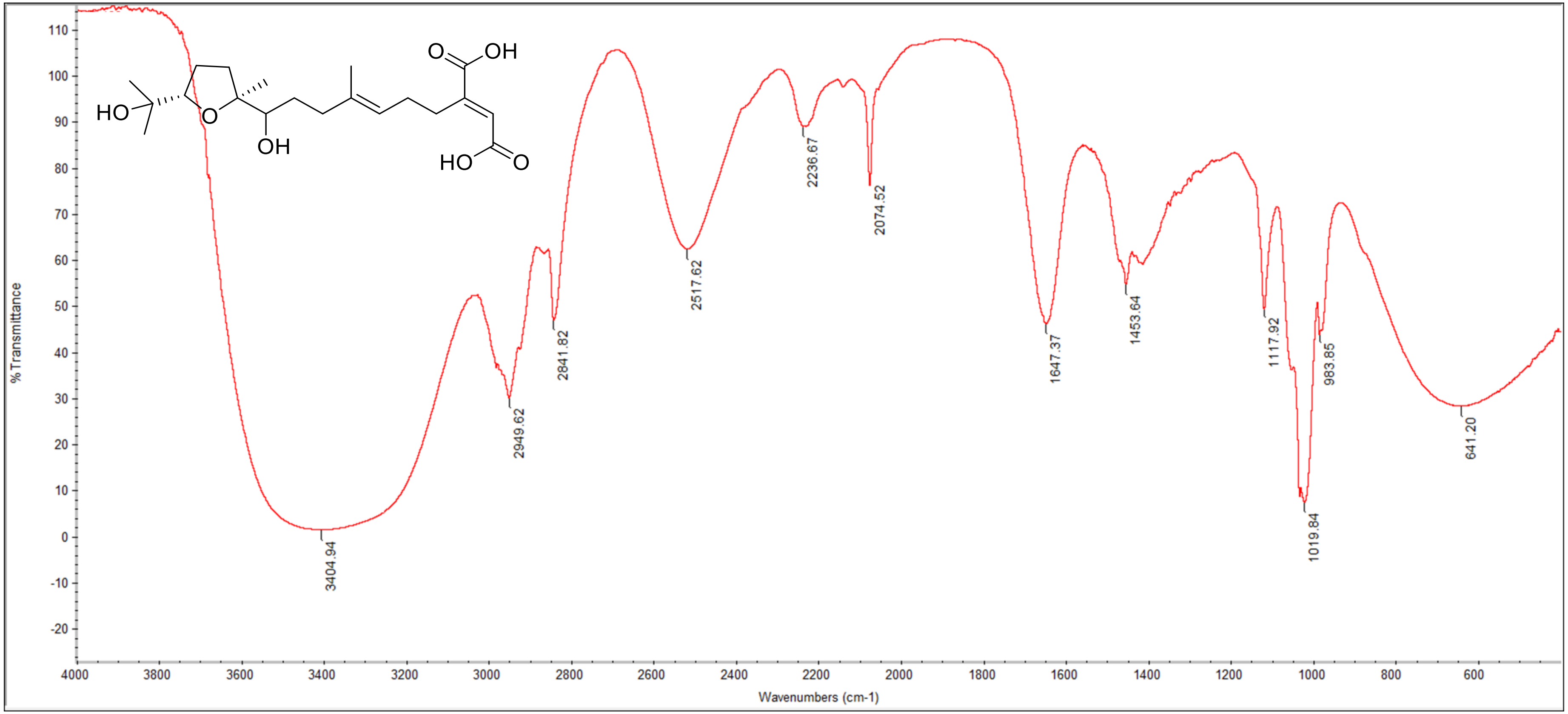

Figure S25. IR spectrum of compound **3** in MeOH.

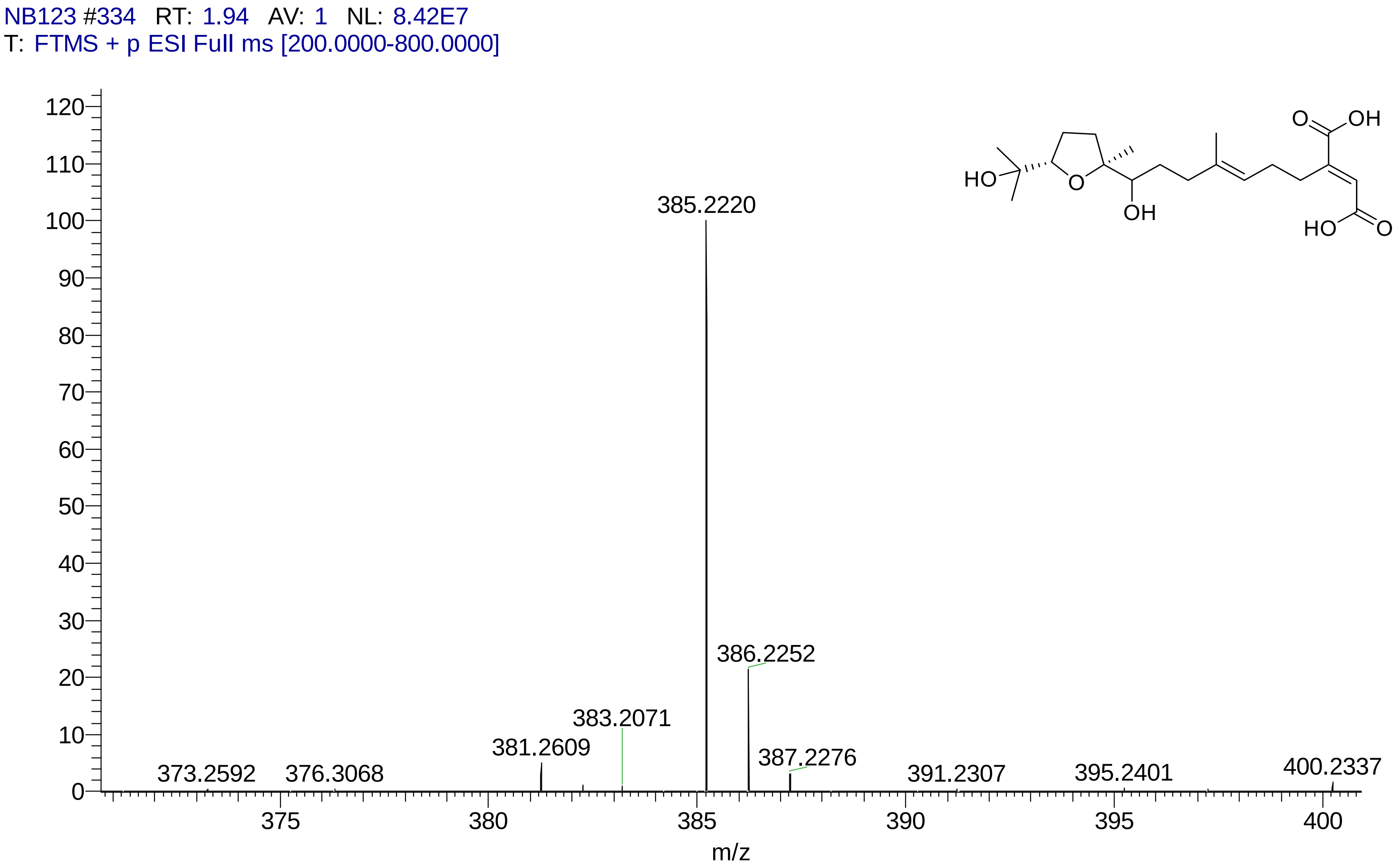

Figure S26. HR-ESI-MS spectrum of compound **3** in MeOH.

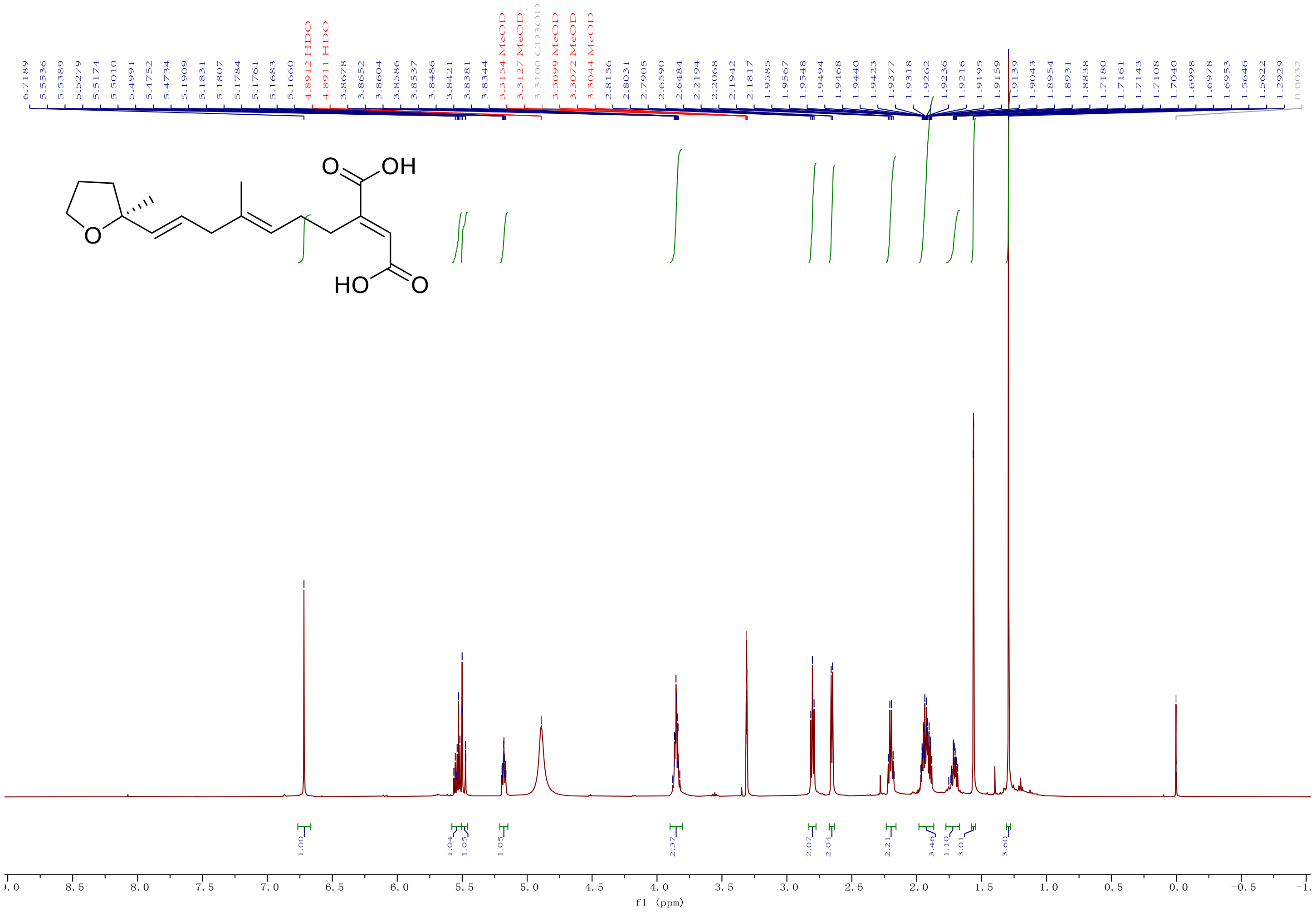

Figure S27. ^{1}H NMR spectrum of compound **4** in MeOH (600 MHz).

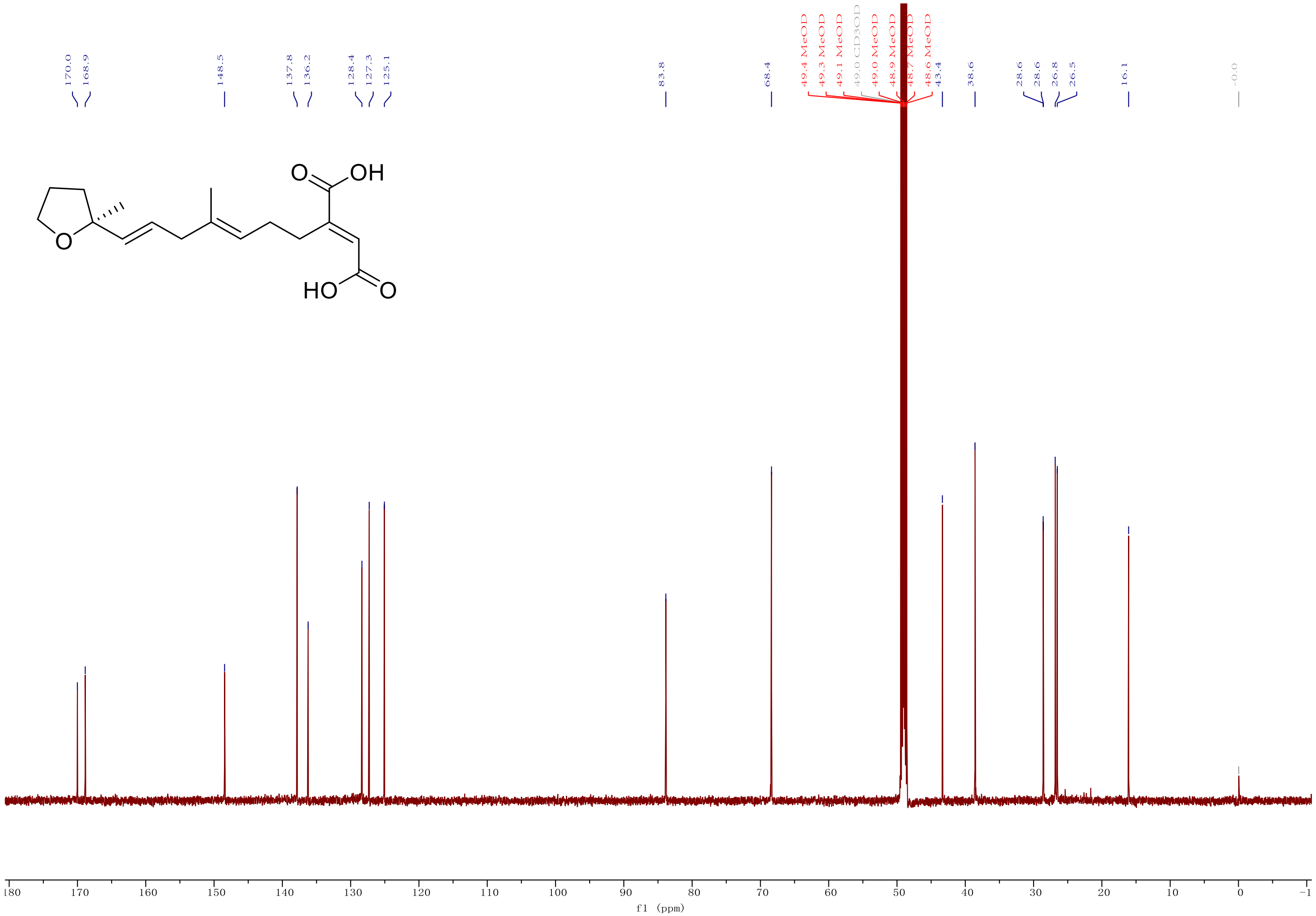

Figure S28. ^{13}C NMR spectrum of compound **4** in MeOH (150 MHz).

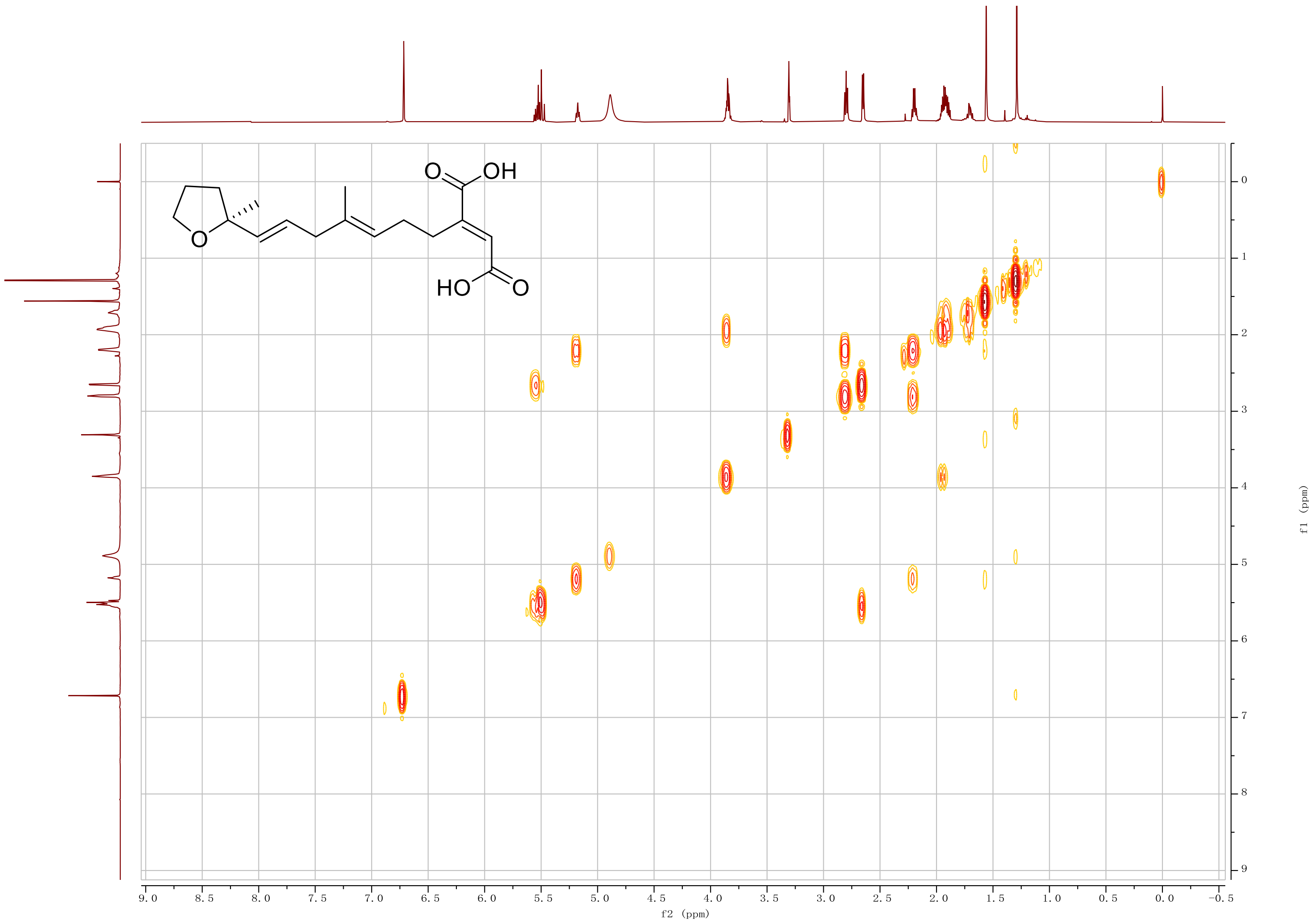

Figure S29. ^{1}H-^{1}H COSY spectrum of compound **4** in MeOH.

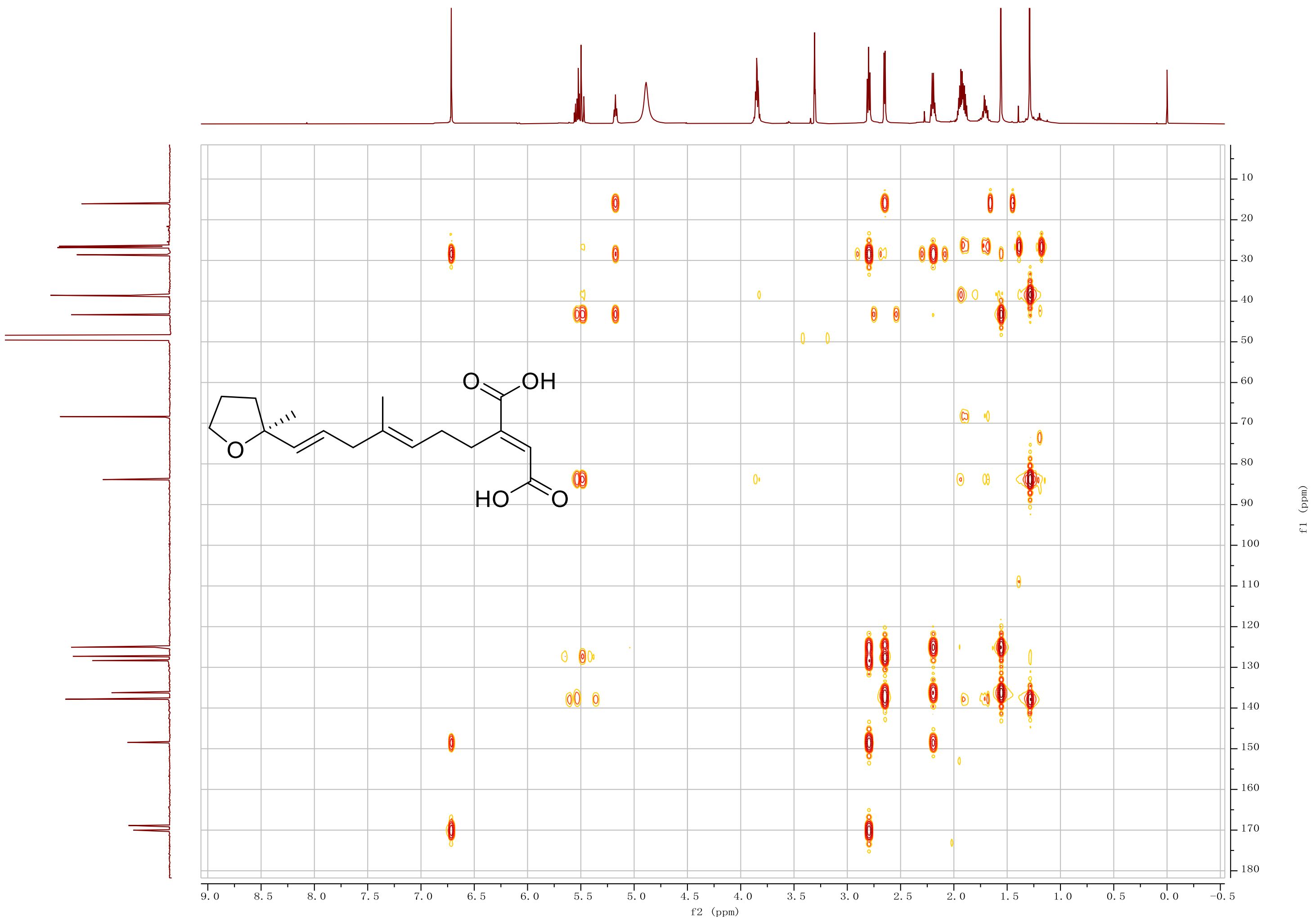

Figure S30. HMBC spectrum of compound **4** in MeOH.

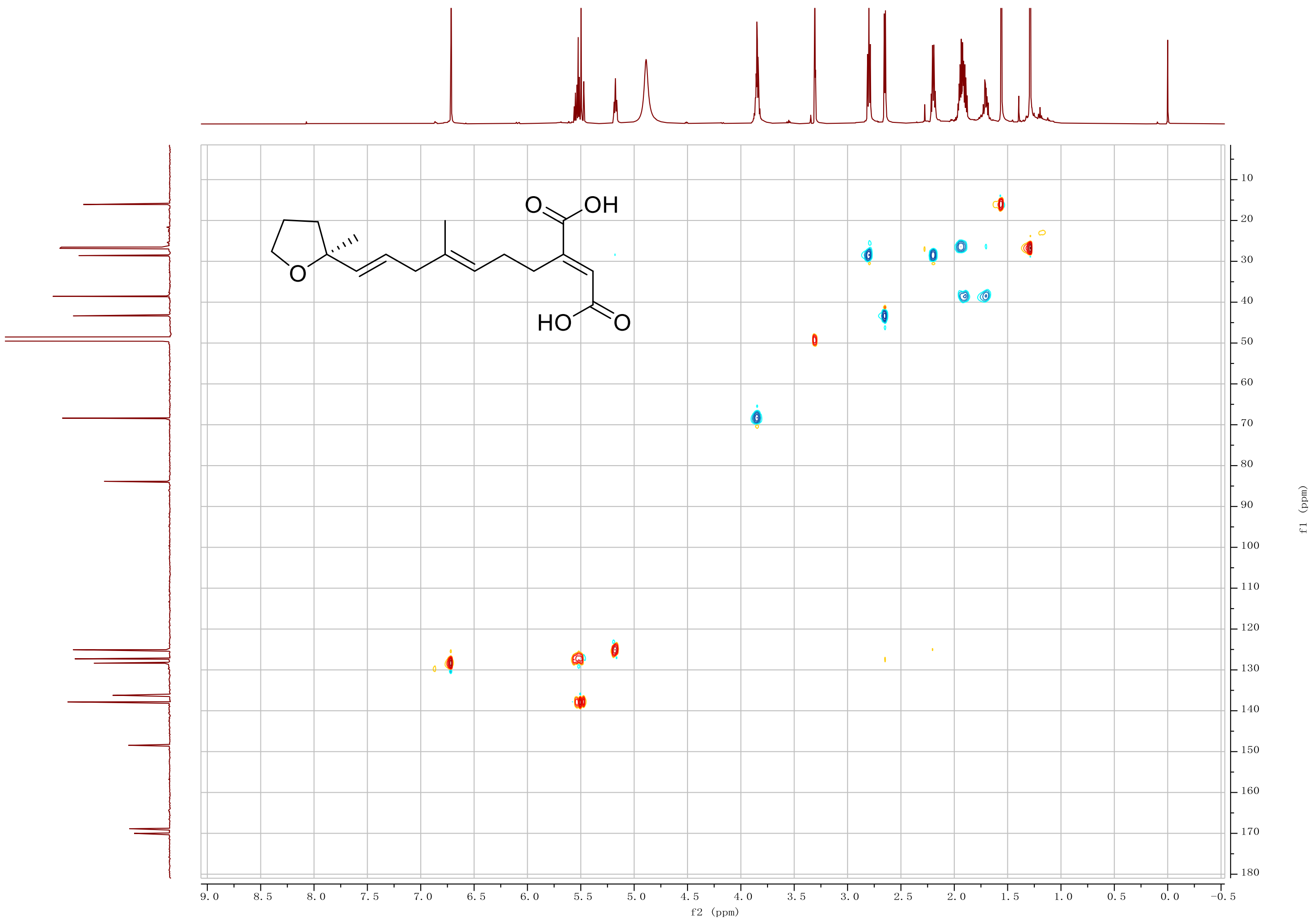

Figure S31. HSQC spectrum of compound **4** in MeOH.

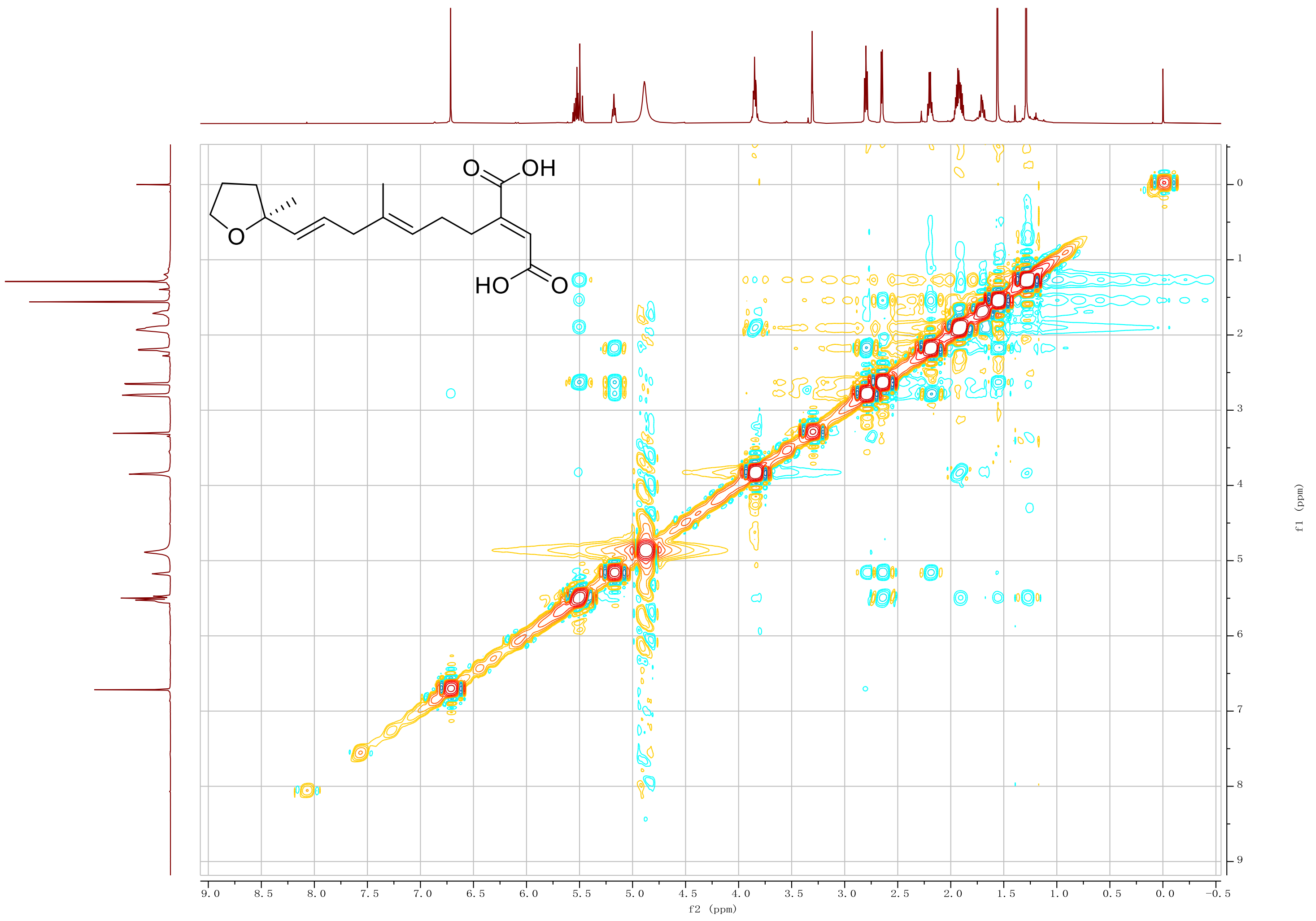

Figure S32. NOESY spectrum of compound **4** in MeOH.

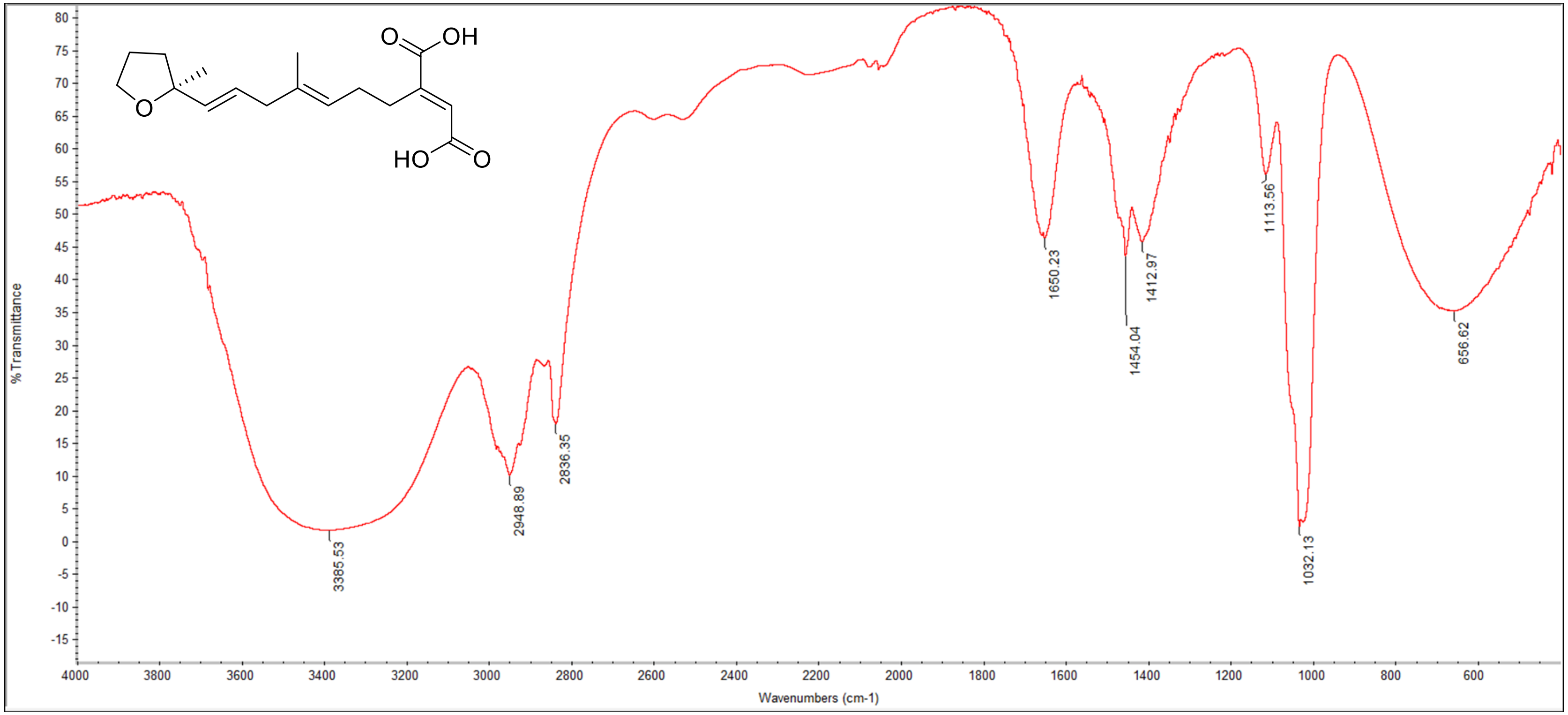

Figure S33. IR spectrum of compound **4** in MeOH.

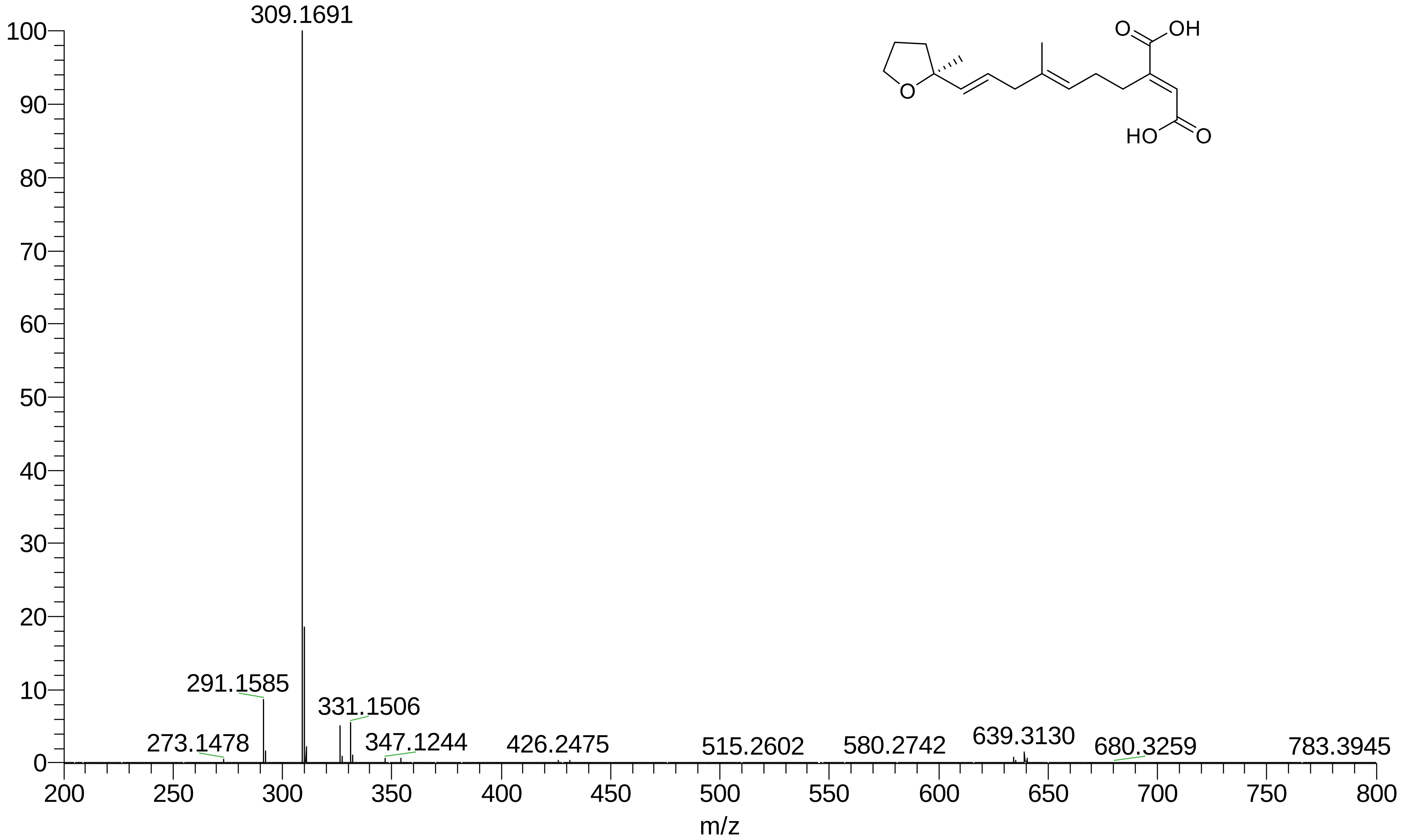

Figure S34. HR-ESI-MS spectrum of compound **4** in MeOH.

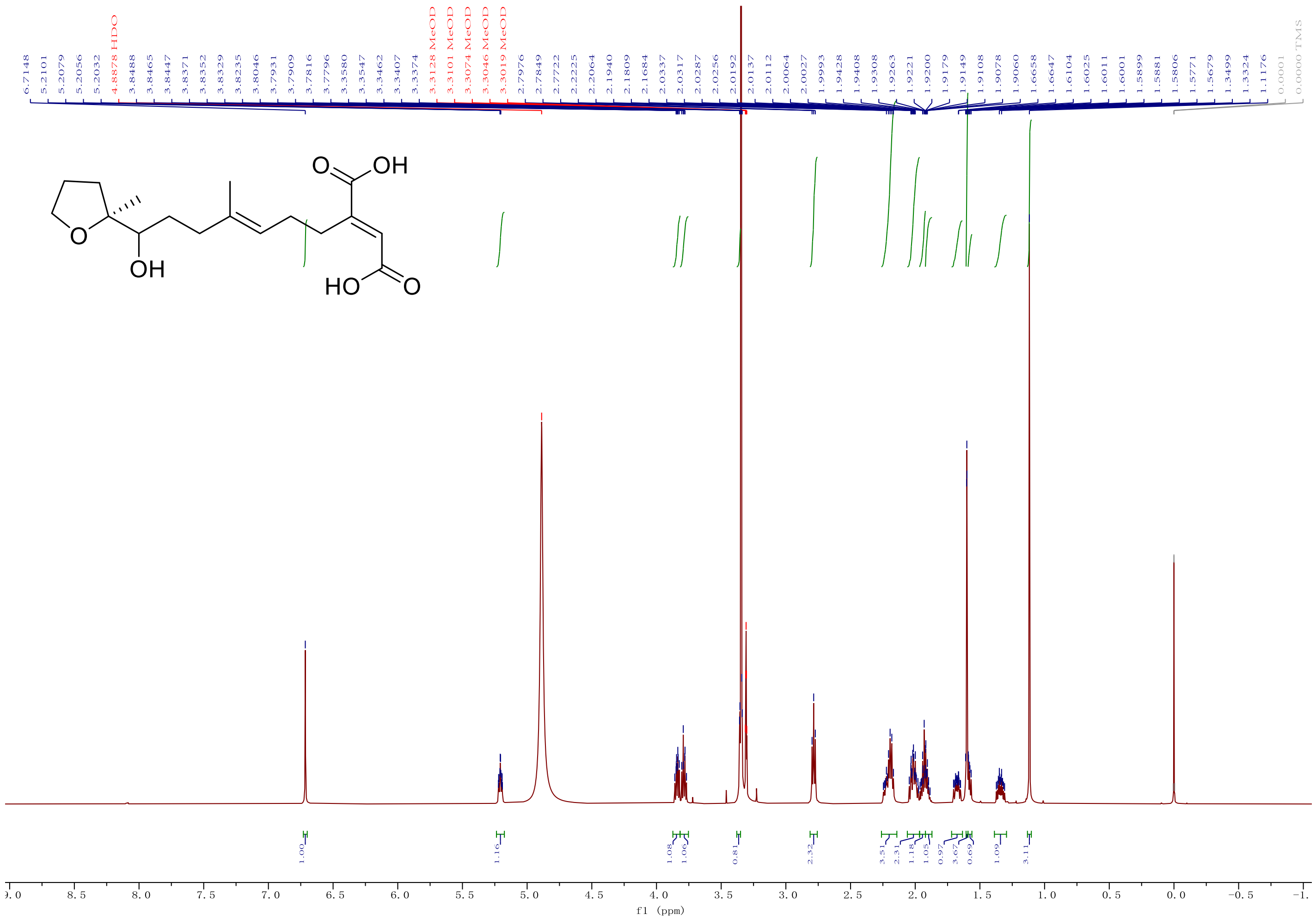

Figure S35. ^{1}H NMR spectrum of compound **5** in MeOH (600 MHz).

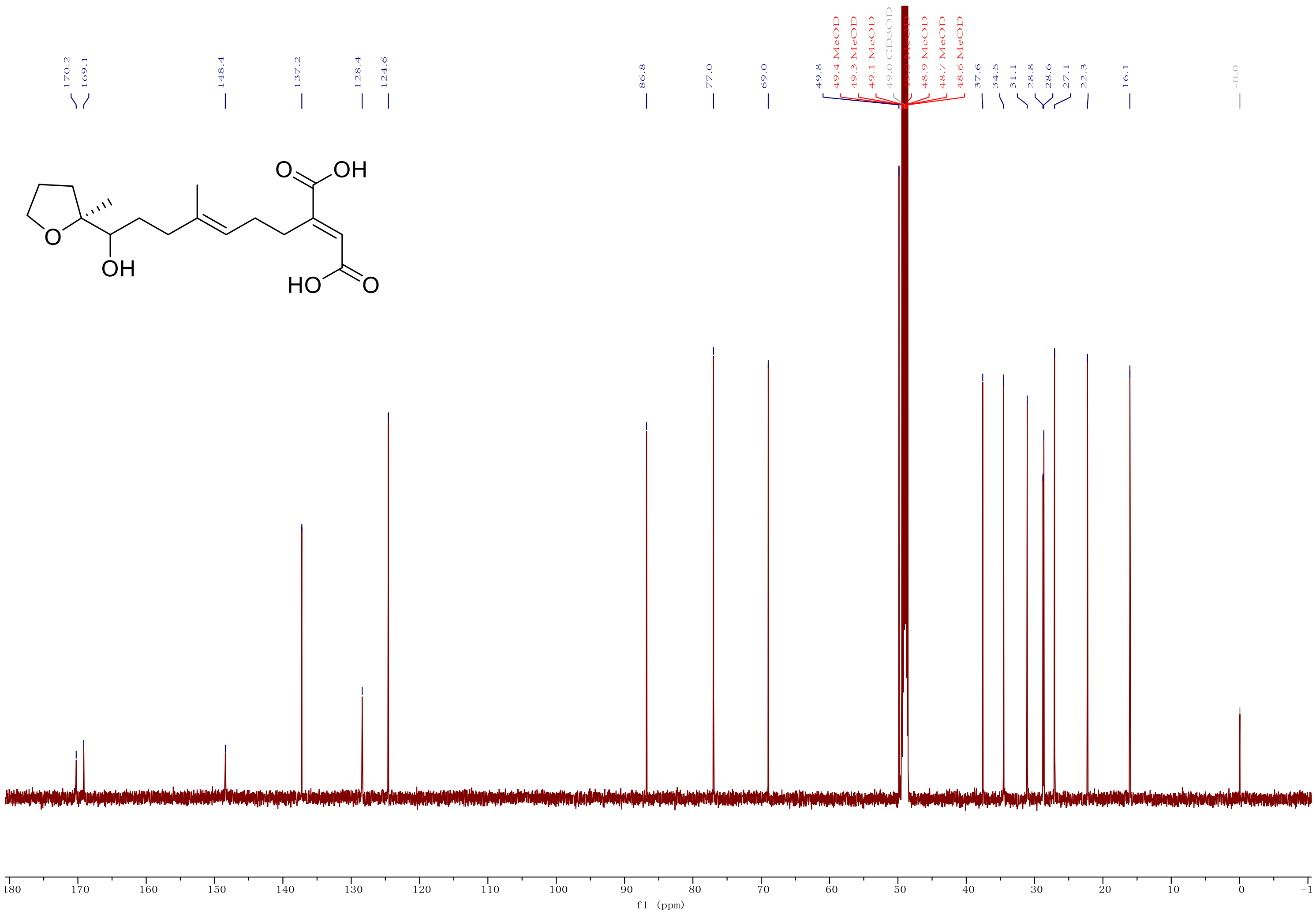

Figure S36. ^{13}C NMR spectrum of compound **5** in MeOH (150 MHz).

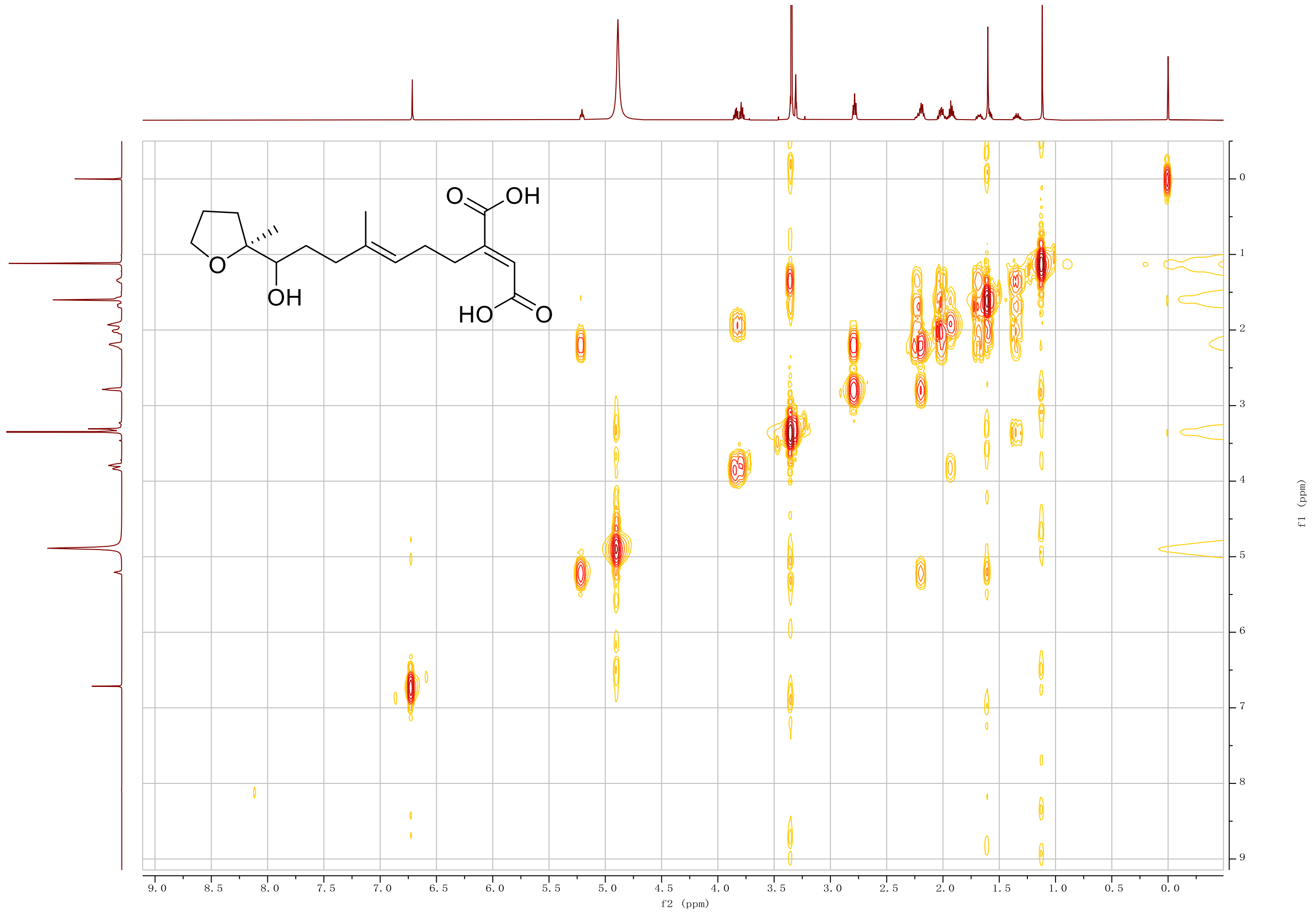

Figure S37. ^{1}H-^{1}H COSY spectrum of compound **5** in MeOH (150 MHz).

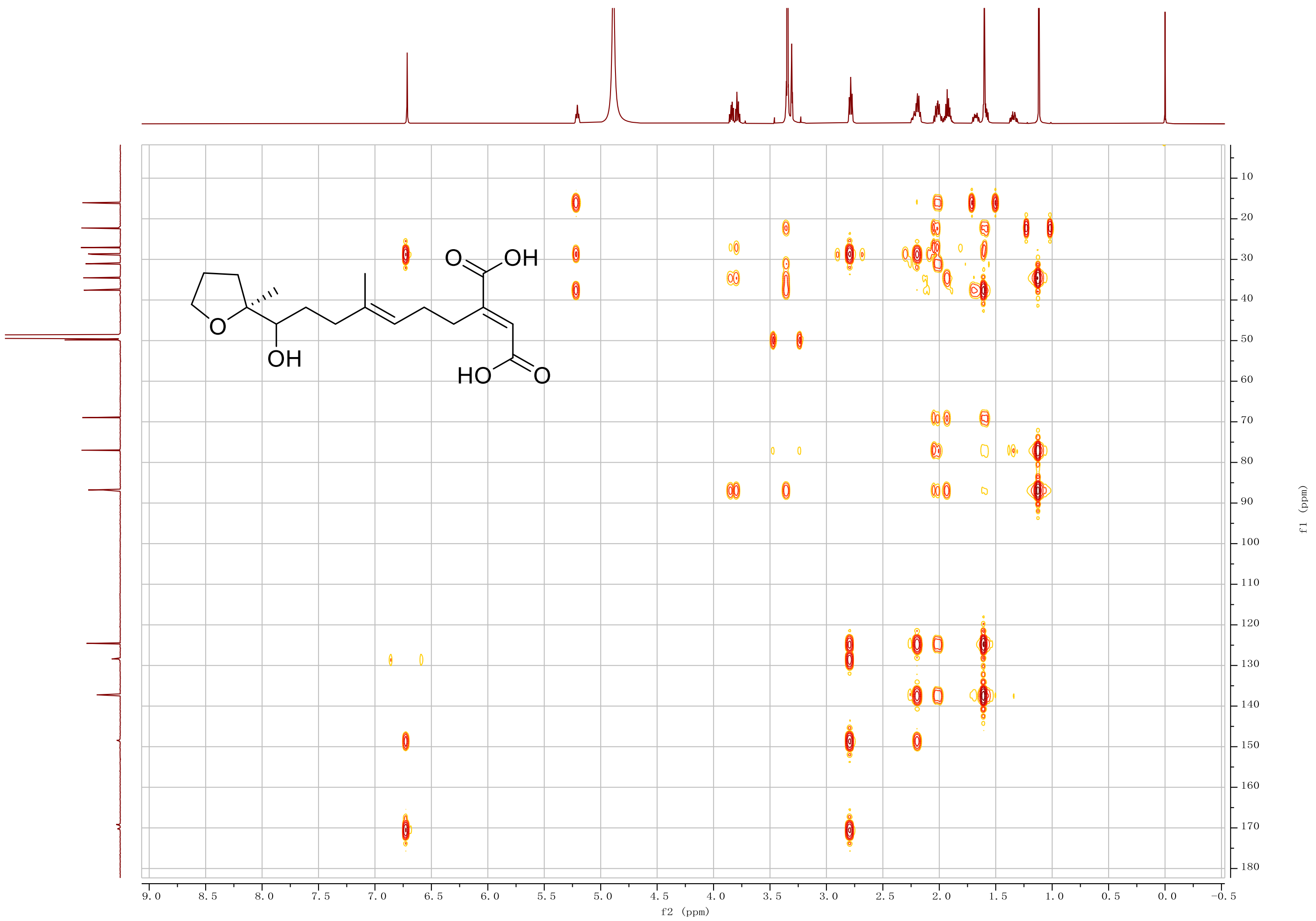

Figure S38. HMBC spectrum of compound **5** in MeOH.

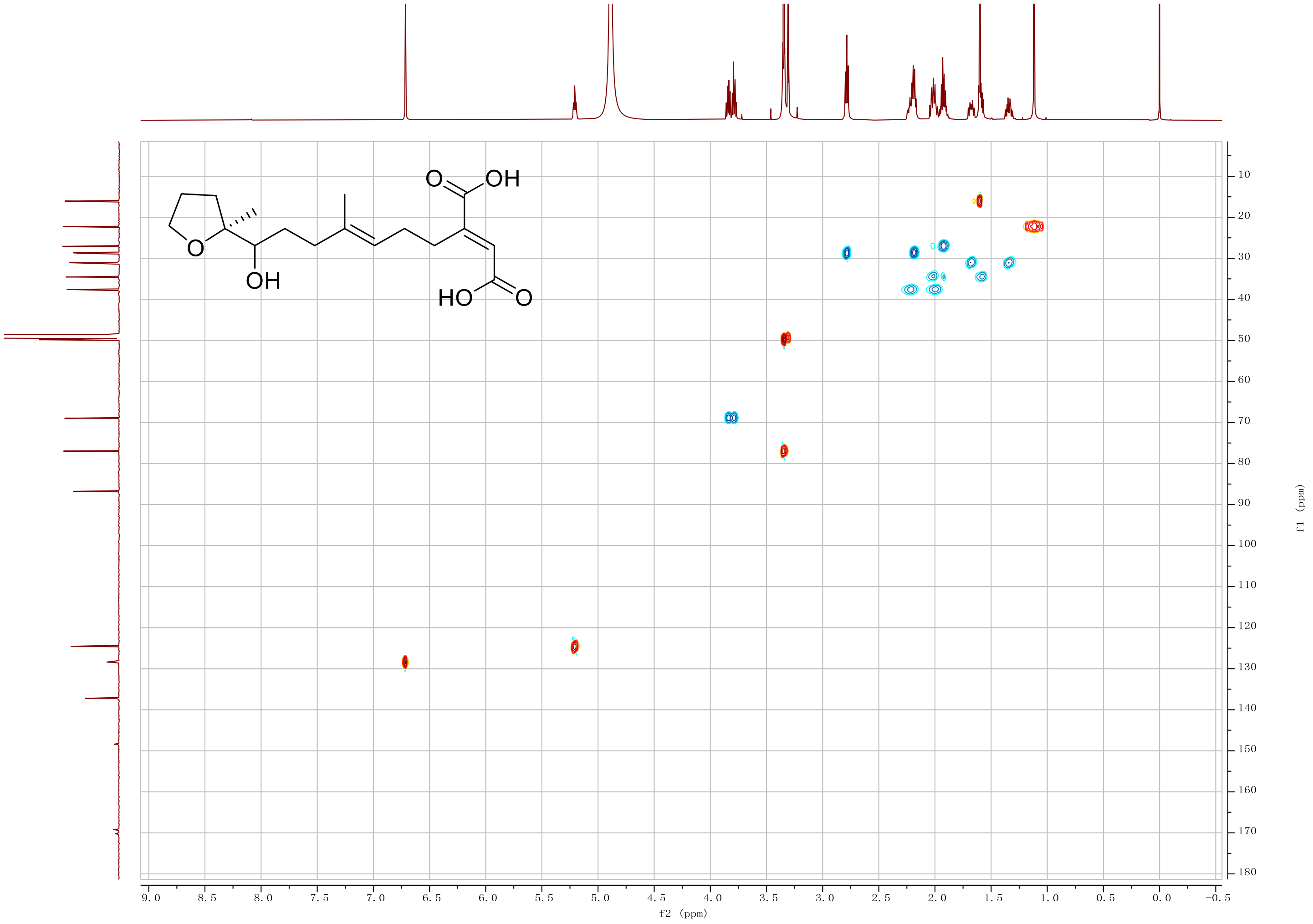

Figure S39. HSQC spectrum of compound **5** in MeOH.

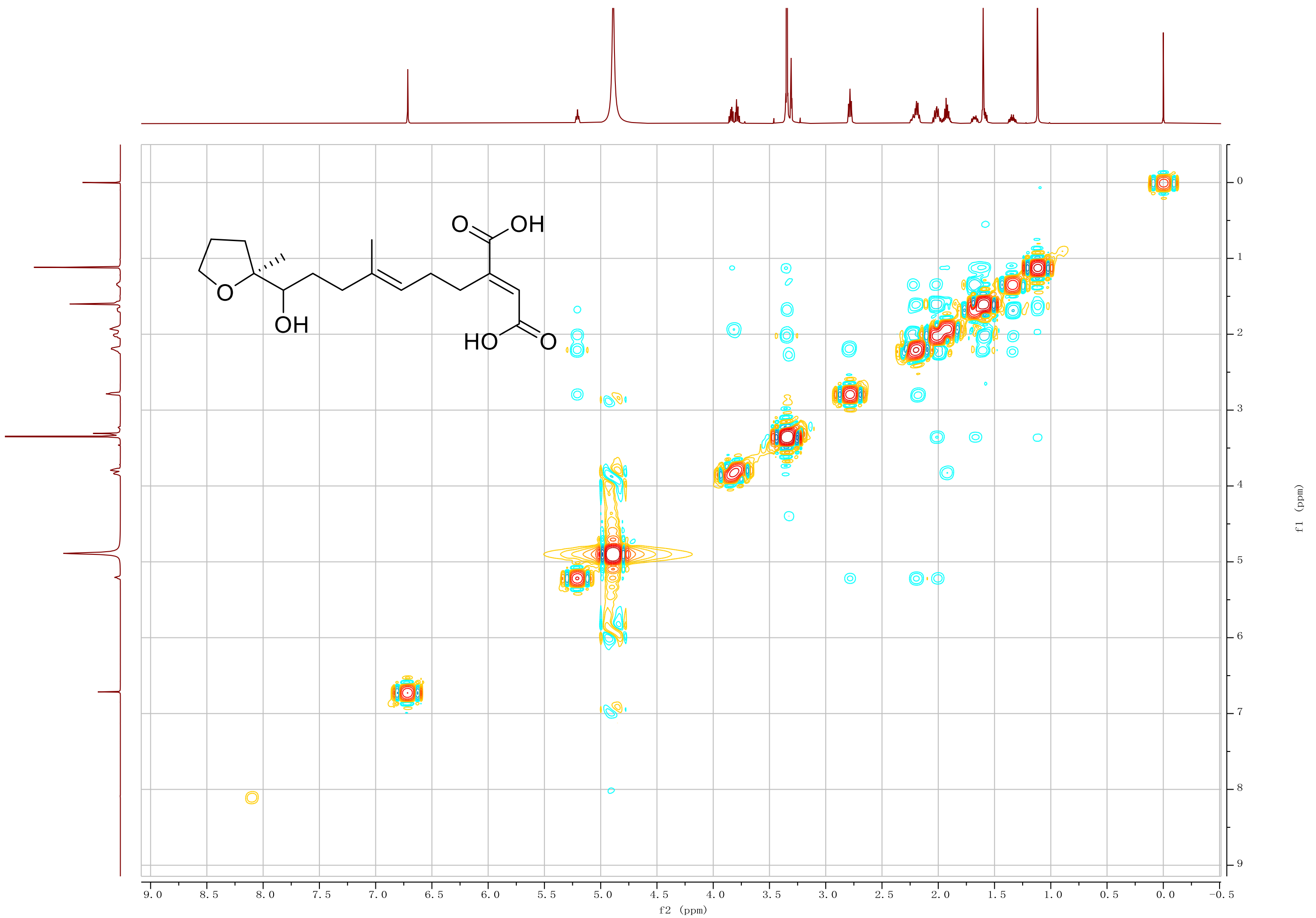

Figure S40. NOESY spectrum of compound **5** in MeOH.

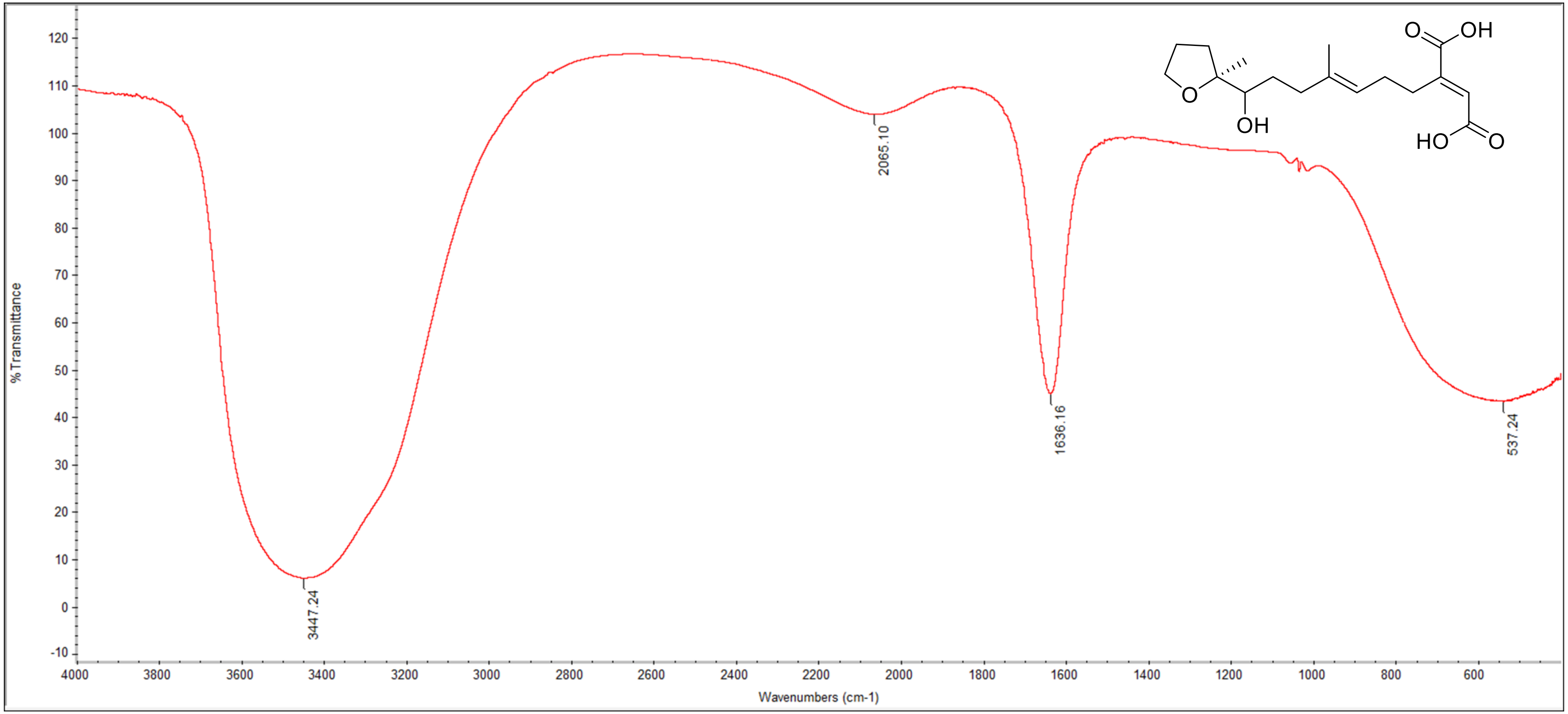

Figure S41. IR spectrum of compound **5** in MeOH.

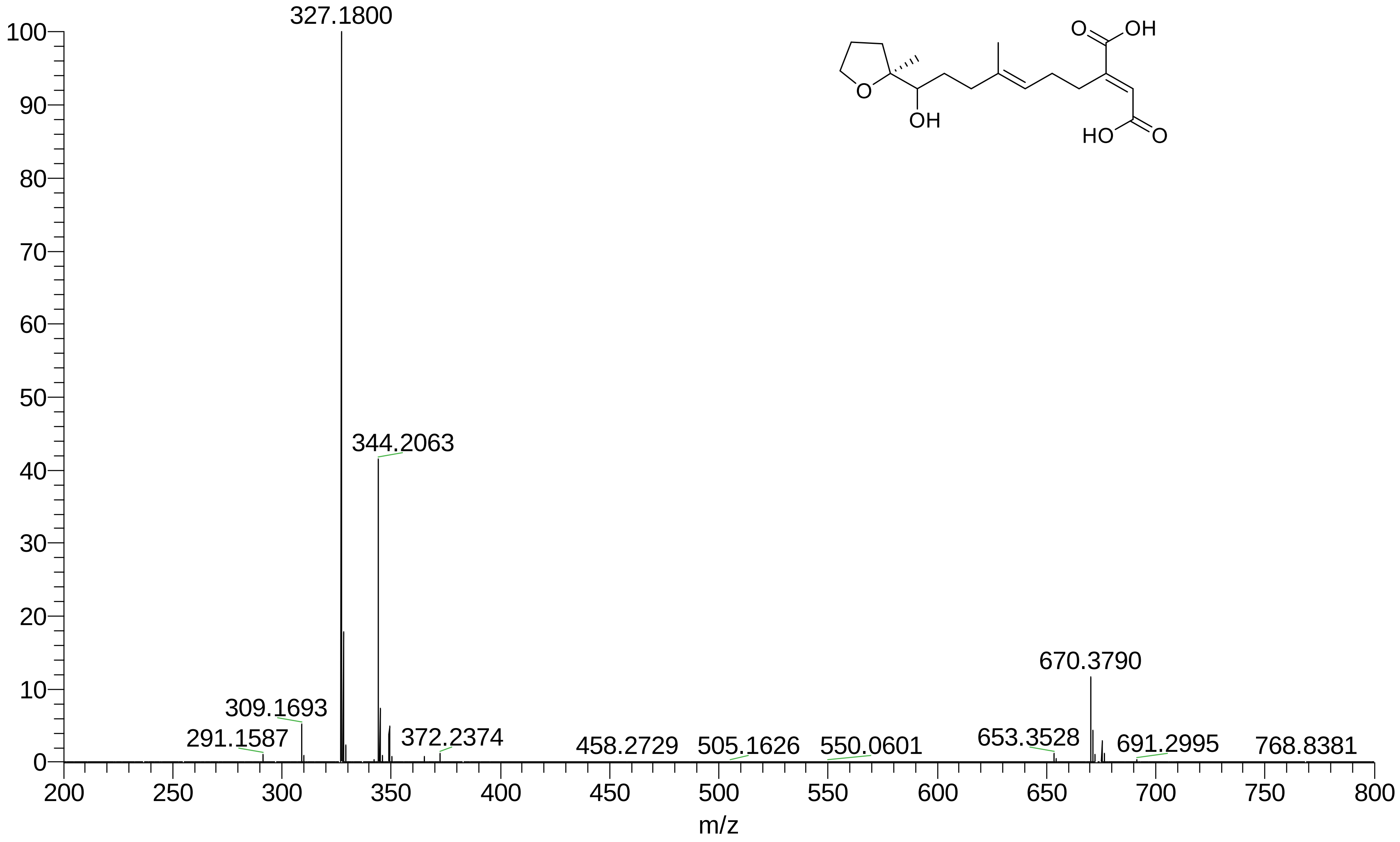

Figure S42. HR-ESI-MS spectrum of compound **5** in MeOH.

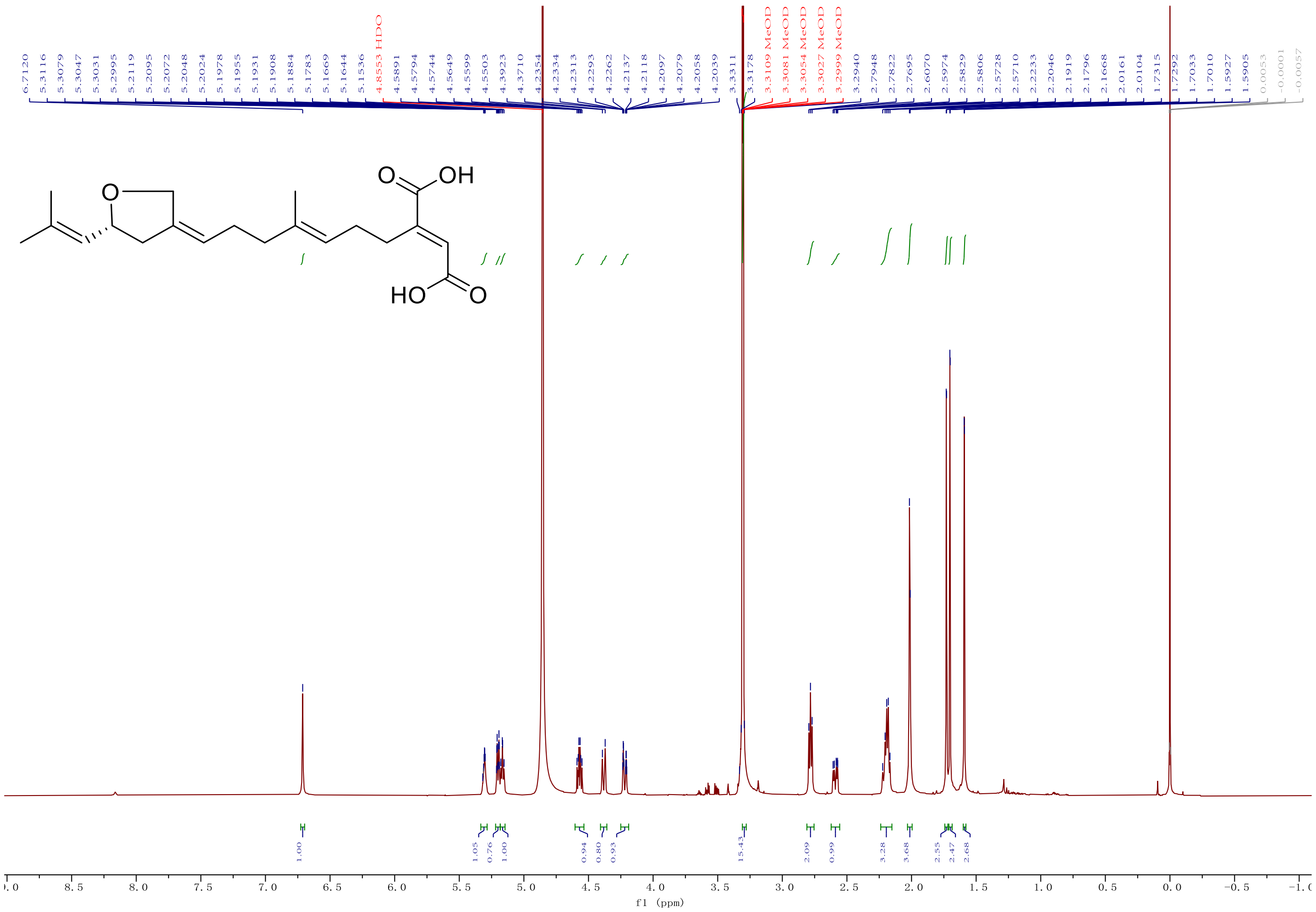

Figure S43. ^{1}H NMR spectrum of compound **6** in MeOH (600 MHz).

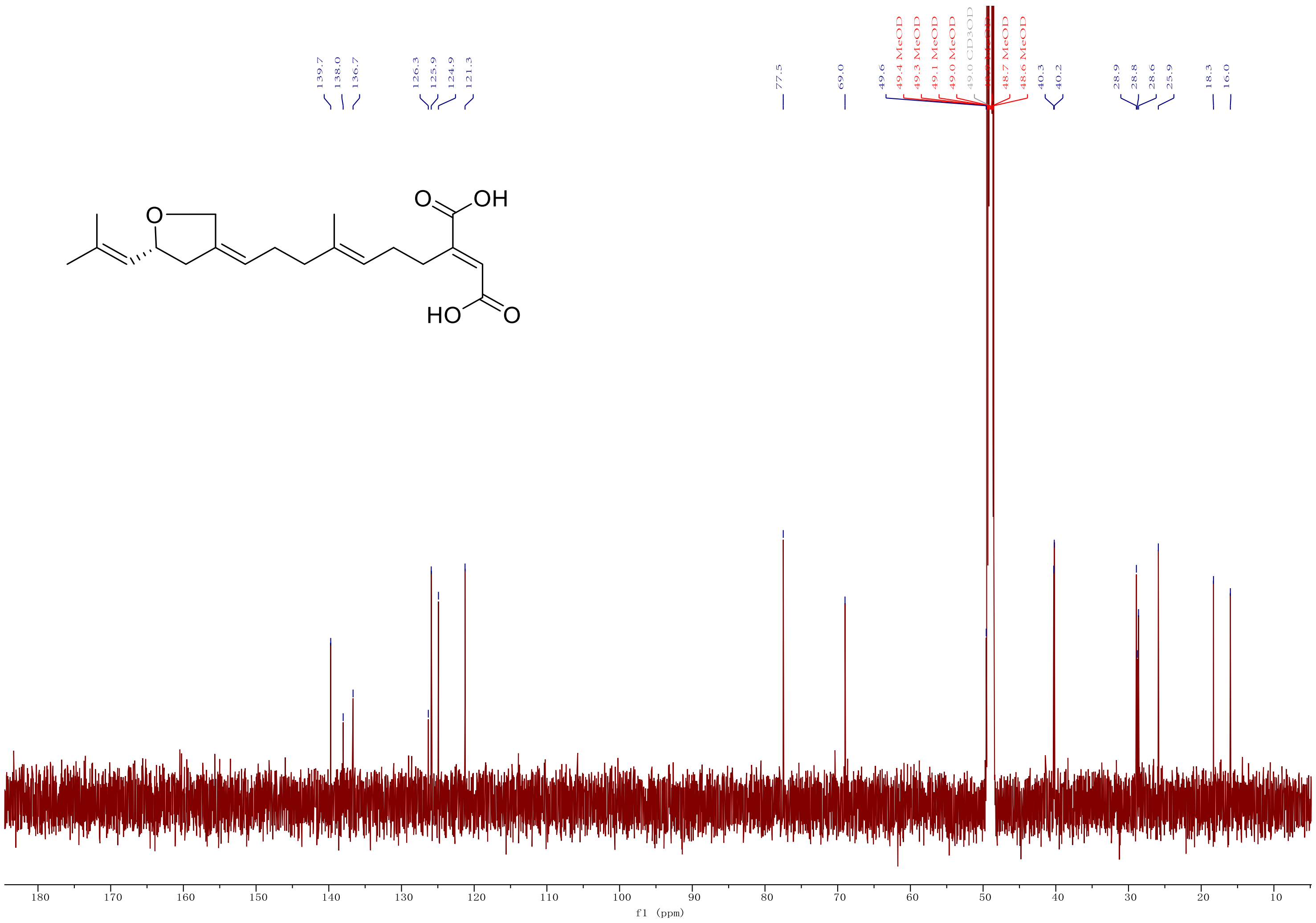

Figure S44. ^{13}C NMR spectrum of compound **6** in MeOH (150 MHz).

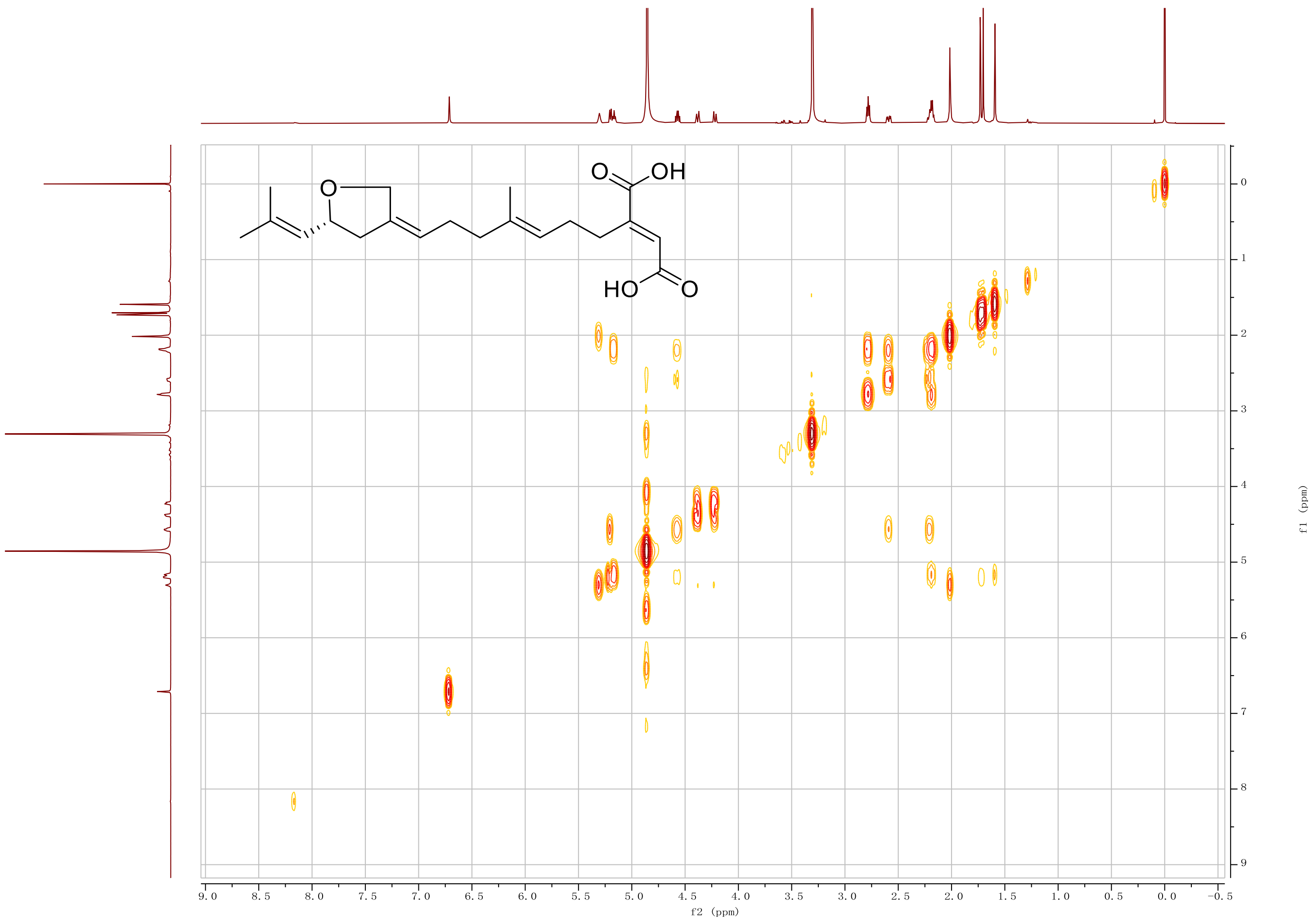

Figure S45. ^{1}H-^{1}H COSY spectrum of compound **6** in MeOH.

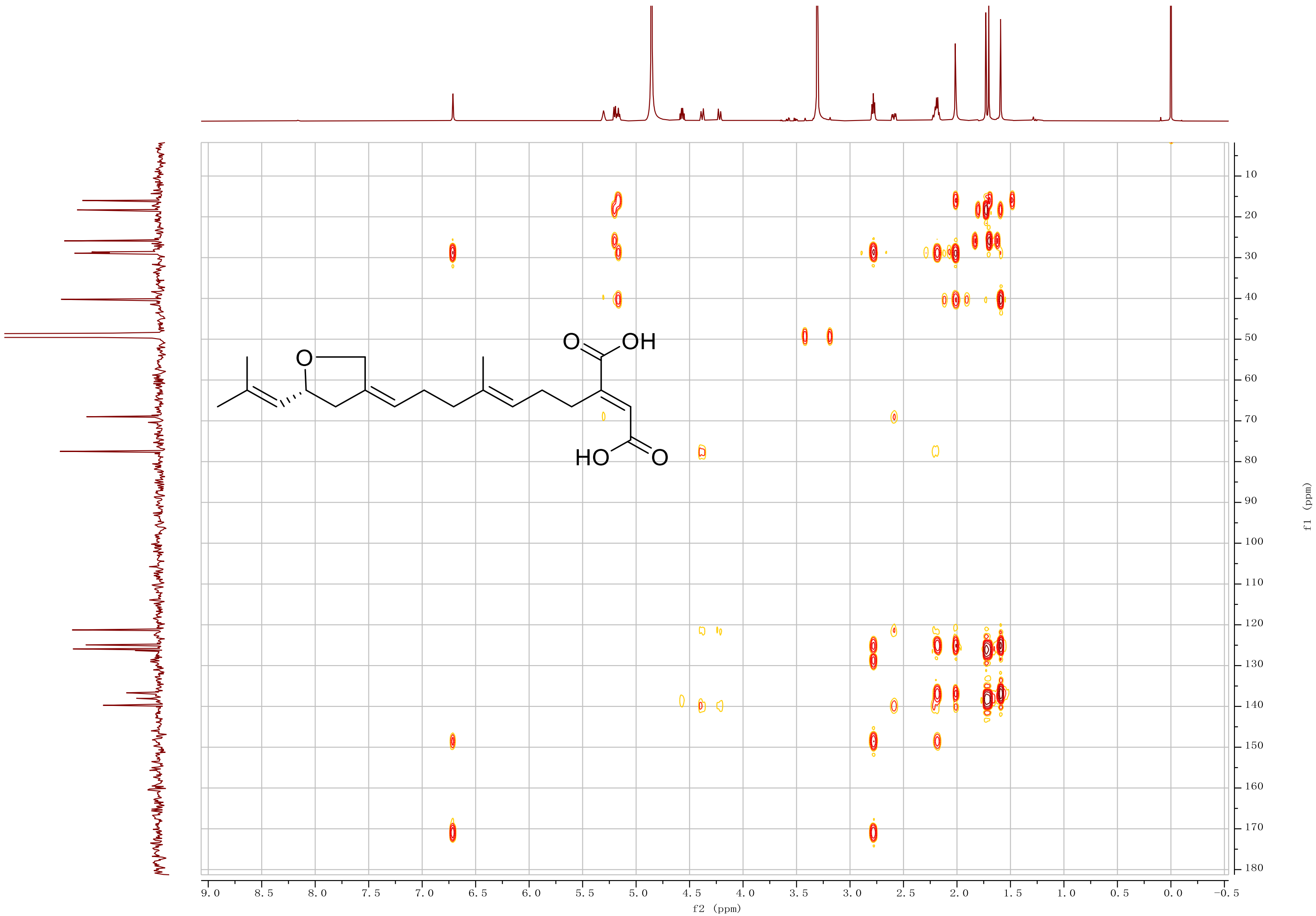

Figure S46. HMBC spectrum of compound **6** in MeOH.

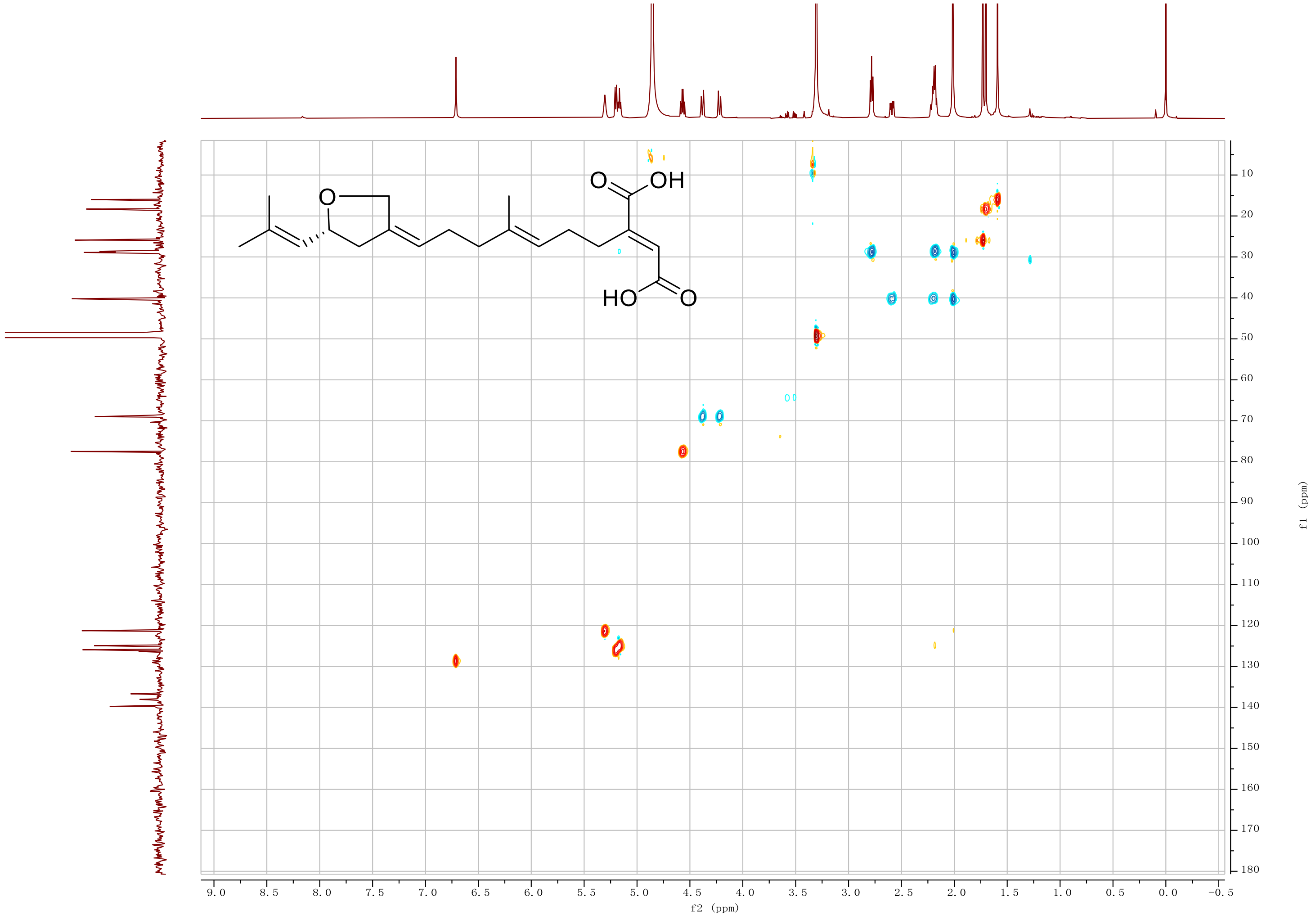

Figure S47. HSQC spectrum of compound **6** in MeOH.

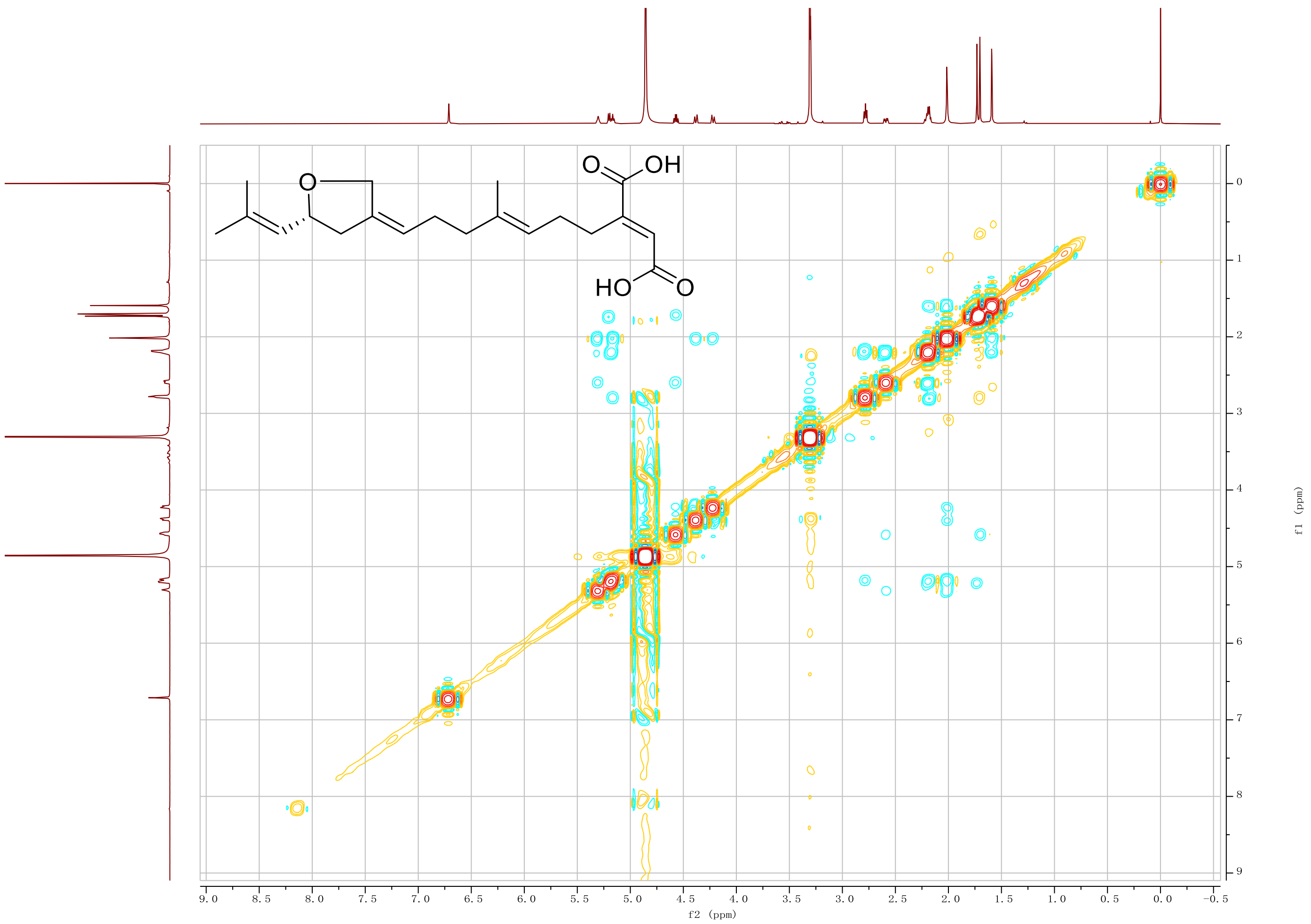

Figure S48. NOESY spectrum of compound **6** in MeOH.

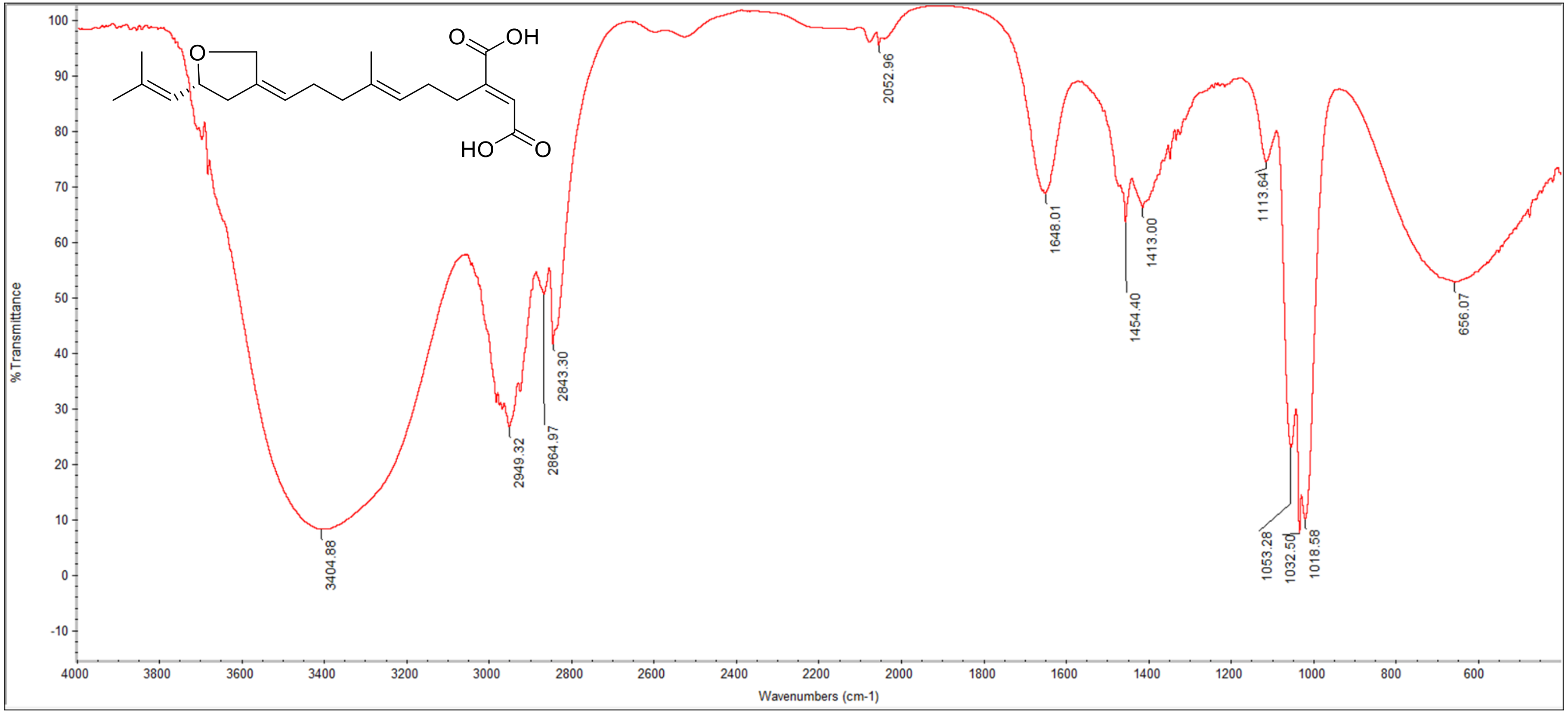

Figure S49. IR spectrum of compound **6** in MeOH.

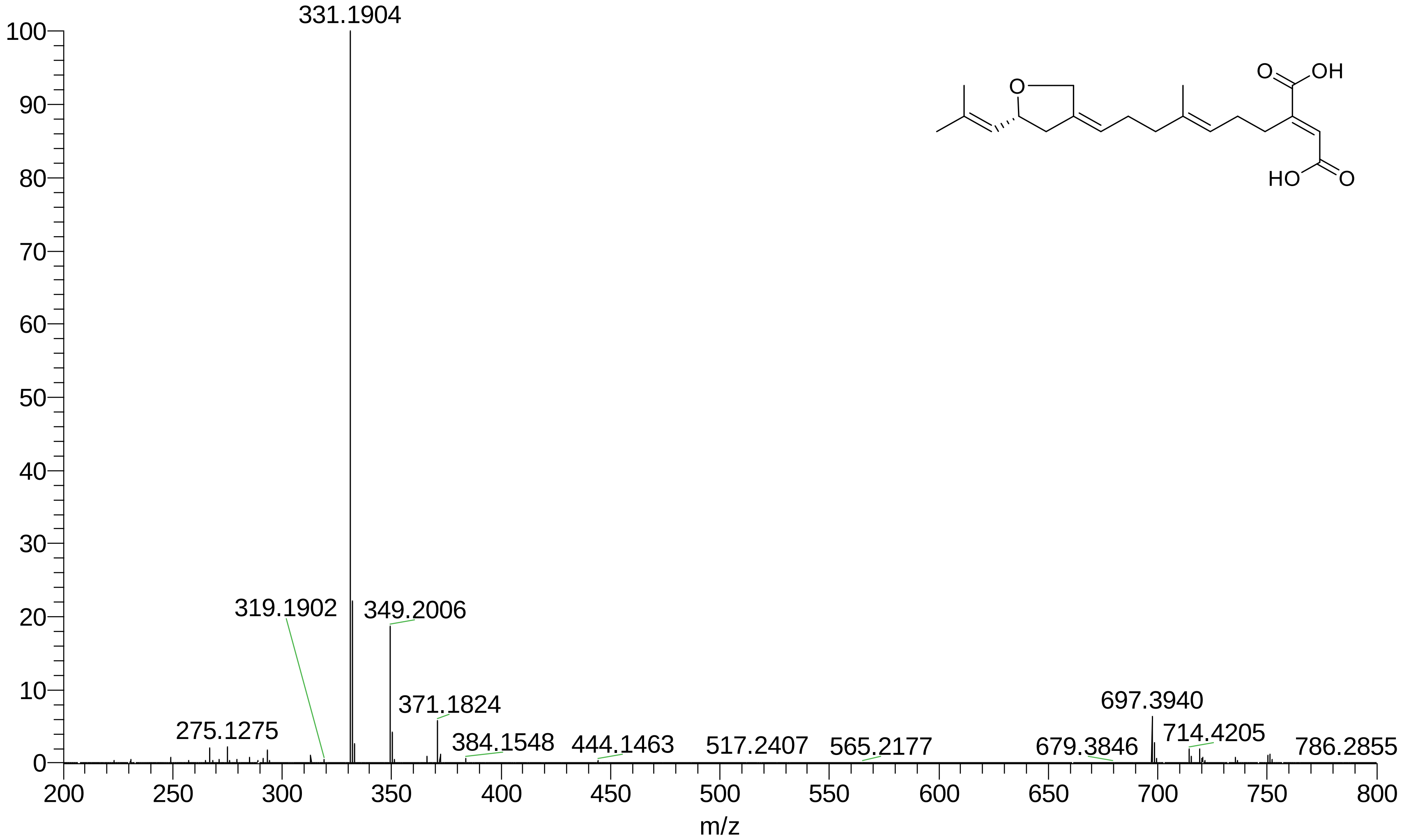

Figure S50. HR-ESI-MS spectrum of compound **6** in MeOH.

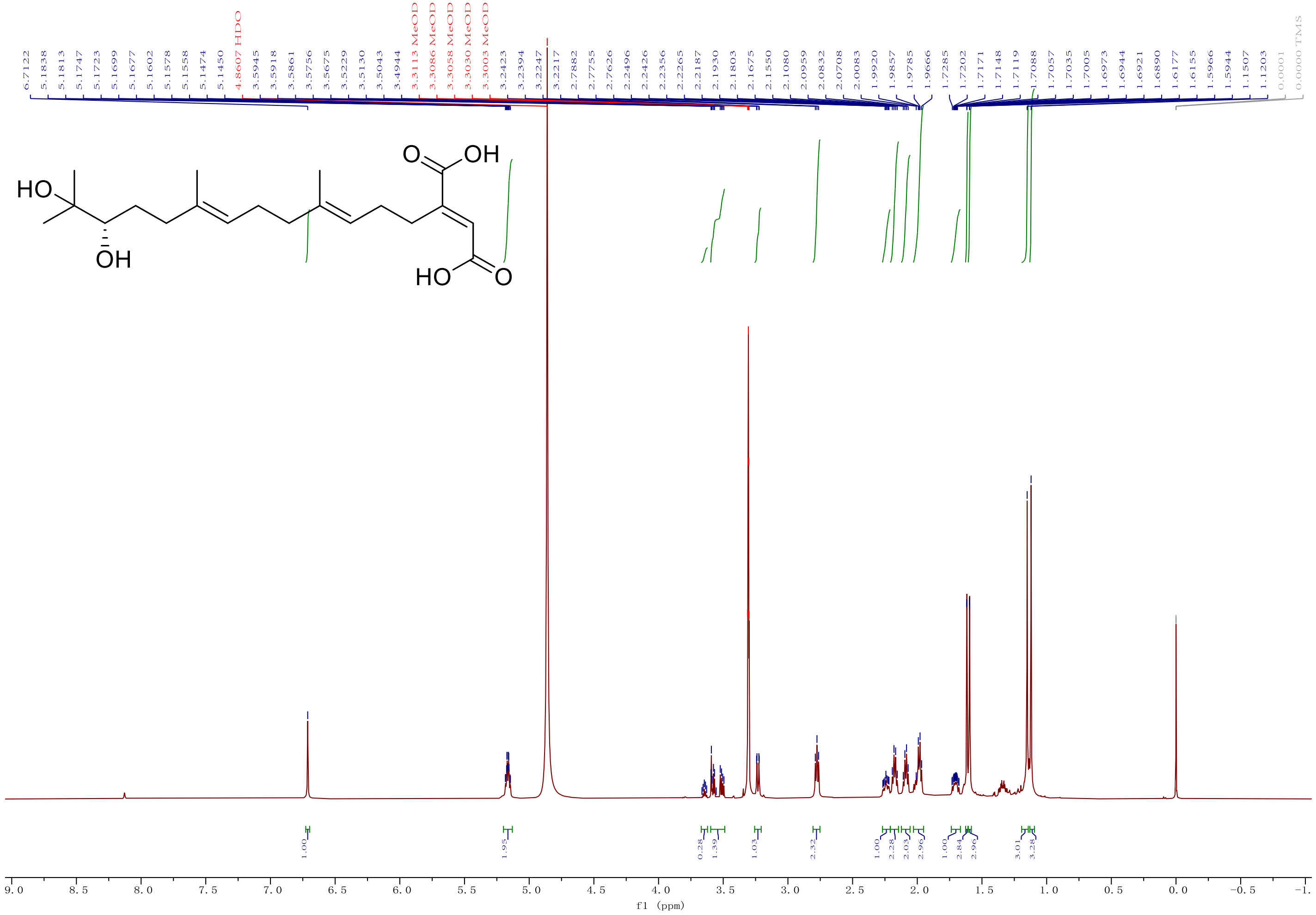

Figure S51. ^{1}H NMR spectrum of compound **7** in MeOH (600 MHz).

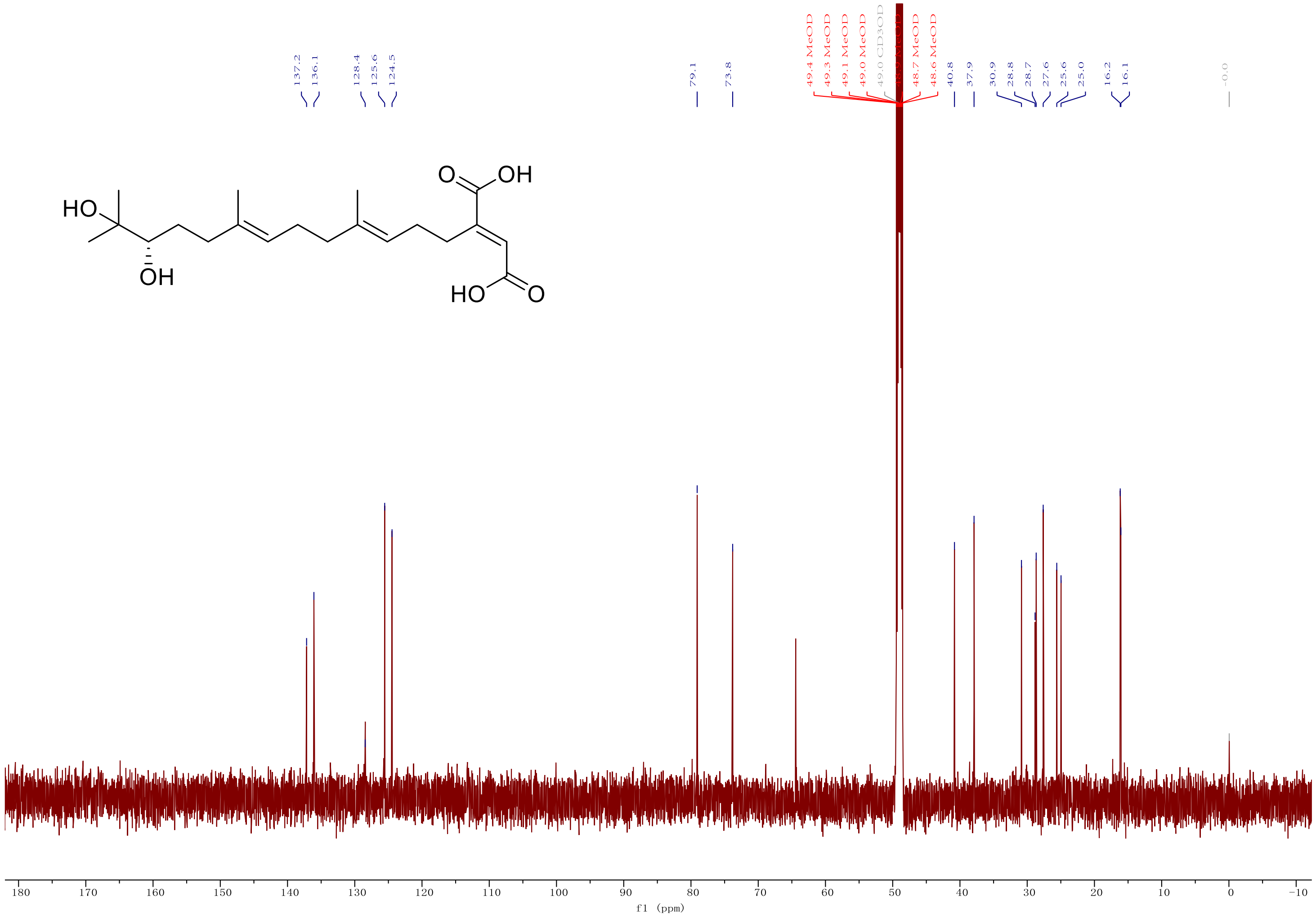

Figure S52. ^{13}C NMR spectrum of compound **7** in MeOH (150 MHz).

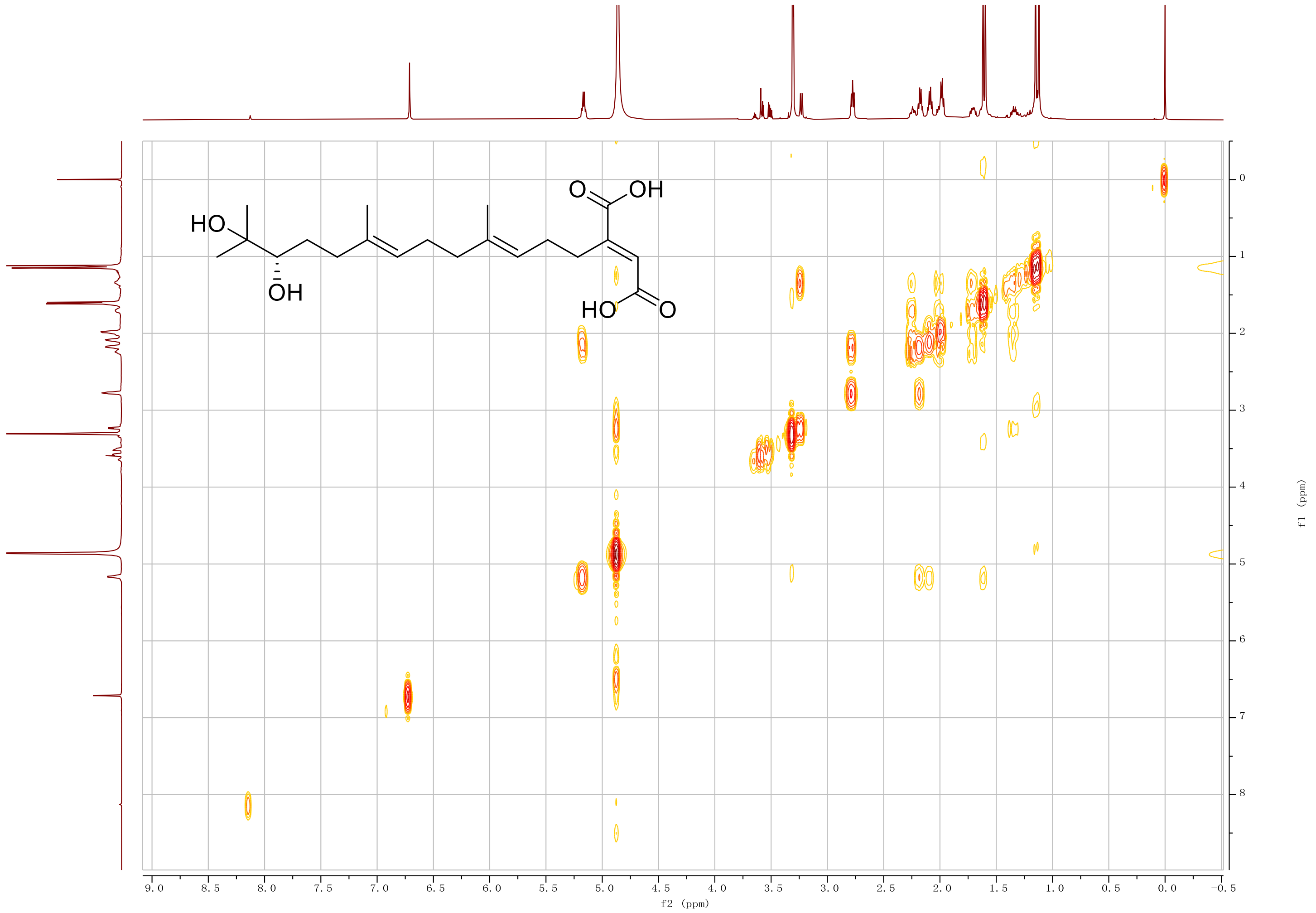

Figure S53. ^{1}H-^{1}H COSY spectrum of compound **7** in MeOH.

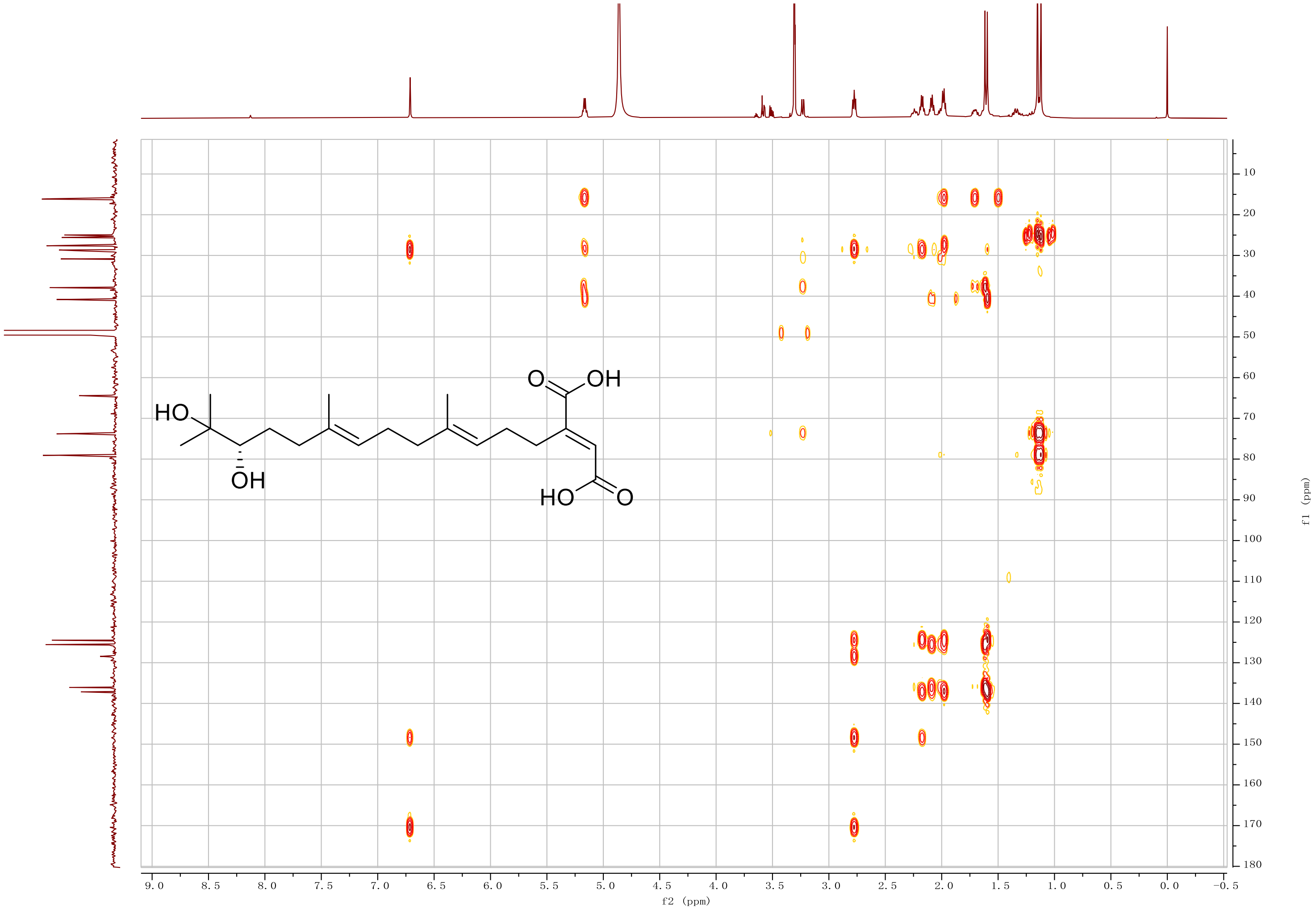

Figure S54. HMBC spectrum of compound **7** in MeOH.

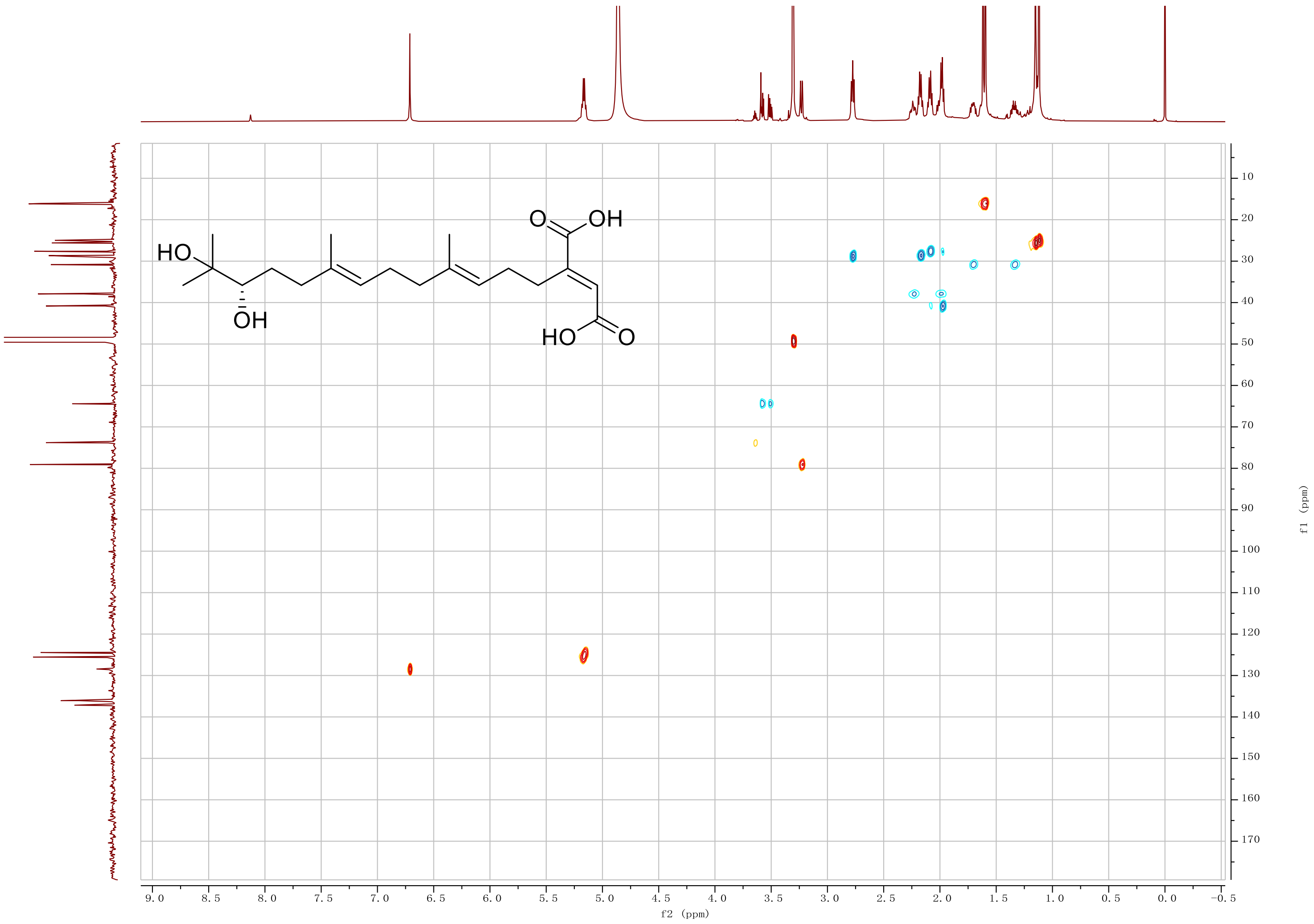

Figure S55. HSQC spectrum of compound **7** in MeOH.

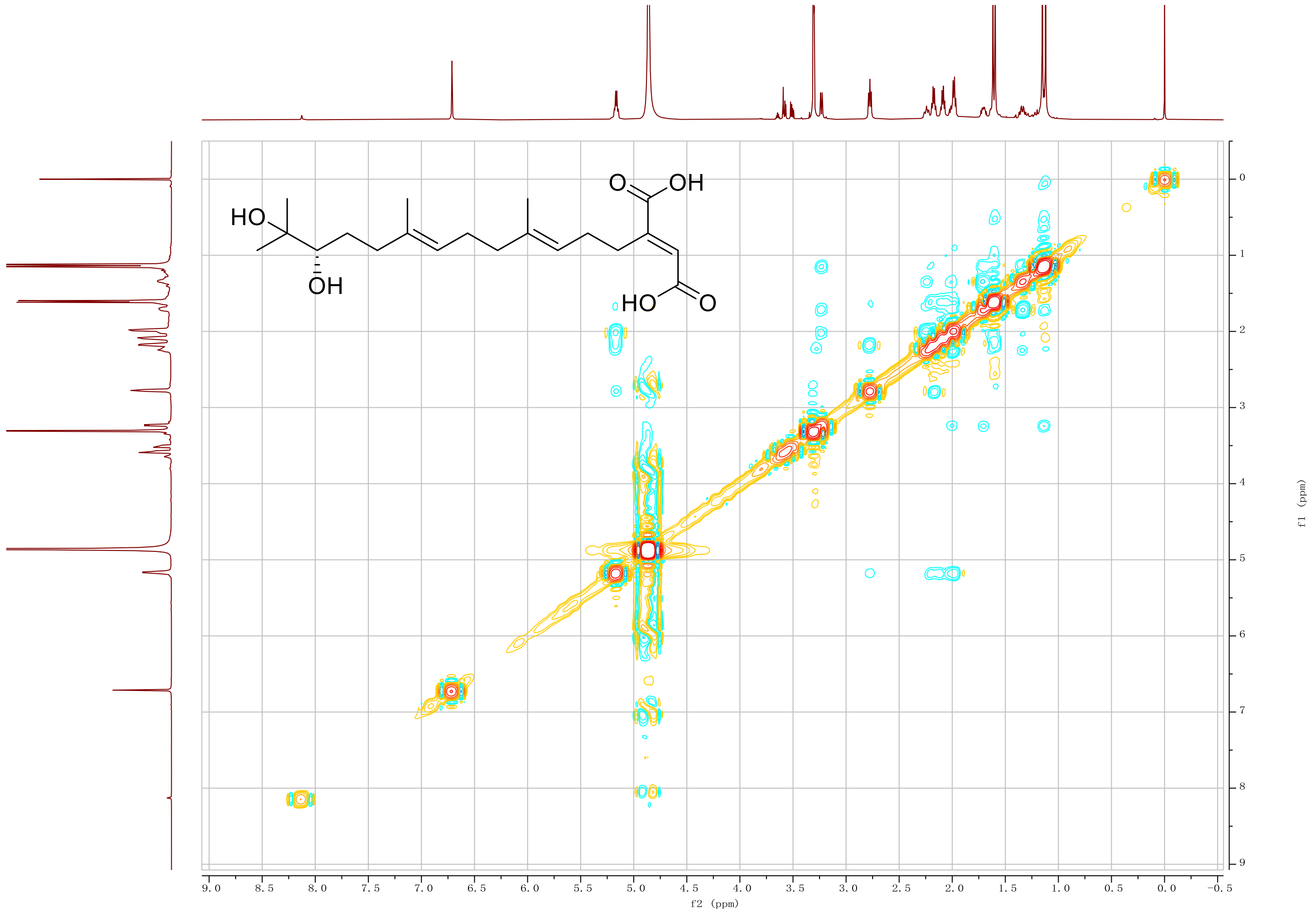

Figure S56. NOESY spectrum of compound **7** in MeOH.

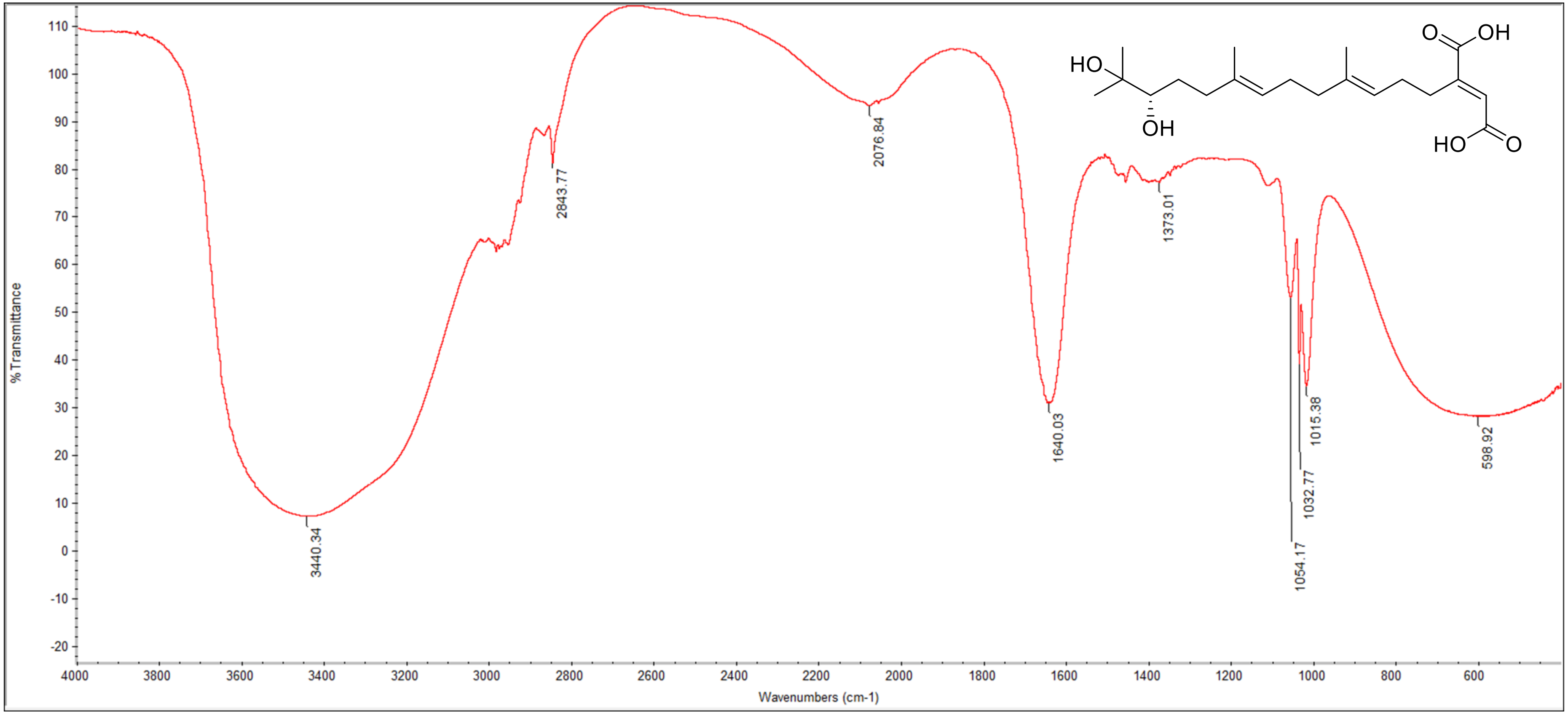

Figure S57. IR spectrum of compound **7** in MeOH.

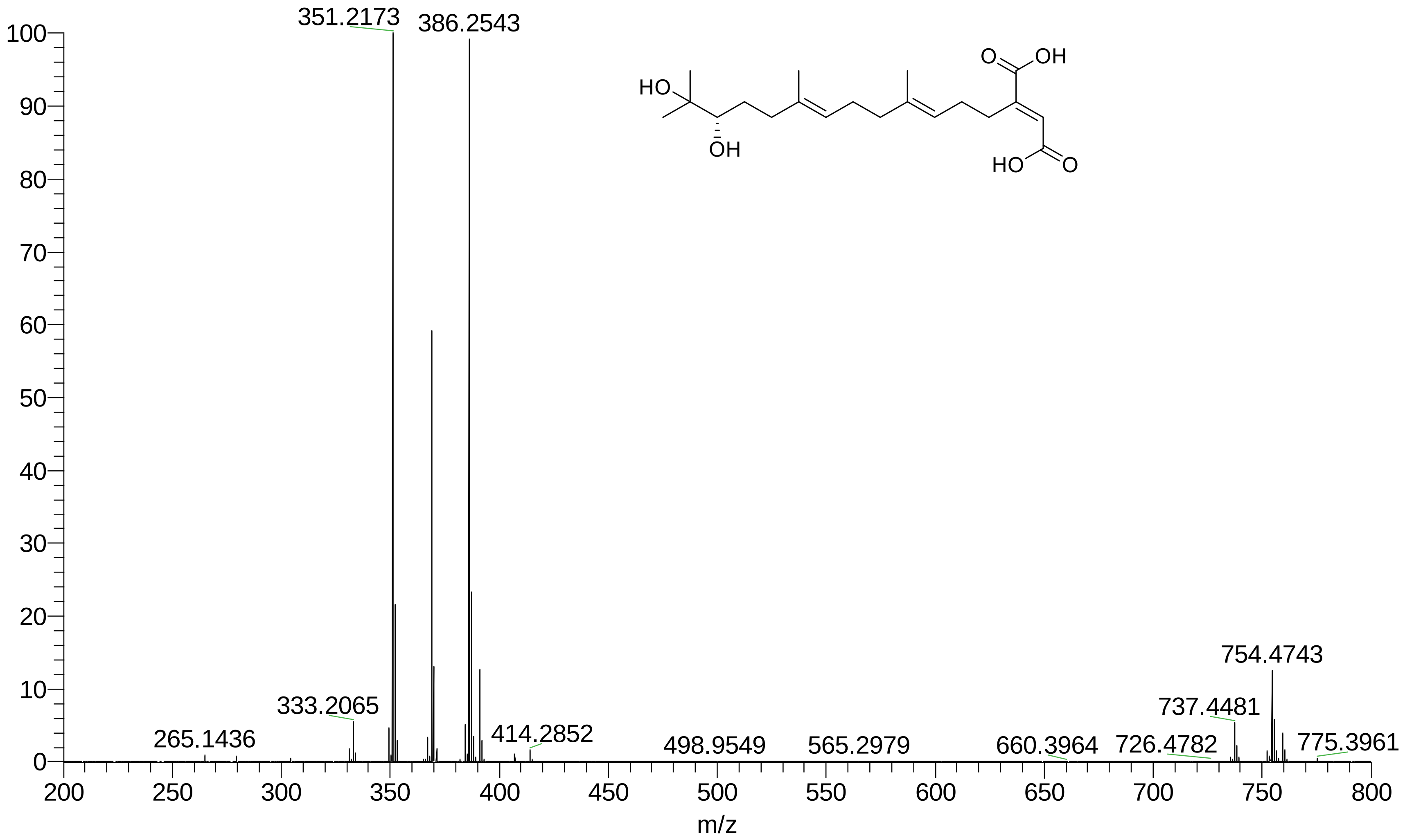

Figure S58. HR-ESI-MS spectrum of compound **7** in MeOH.

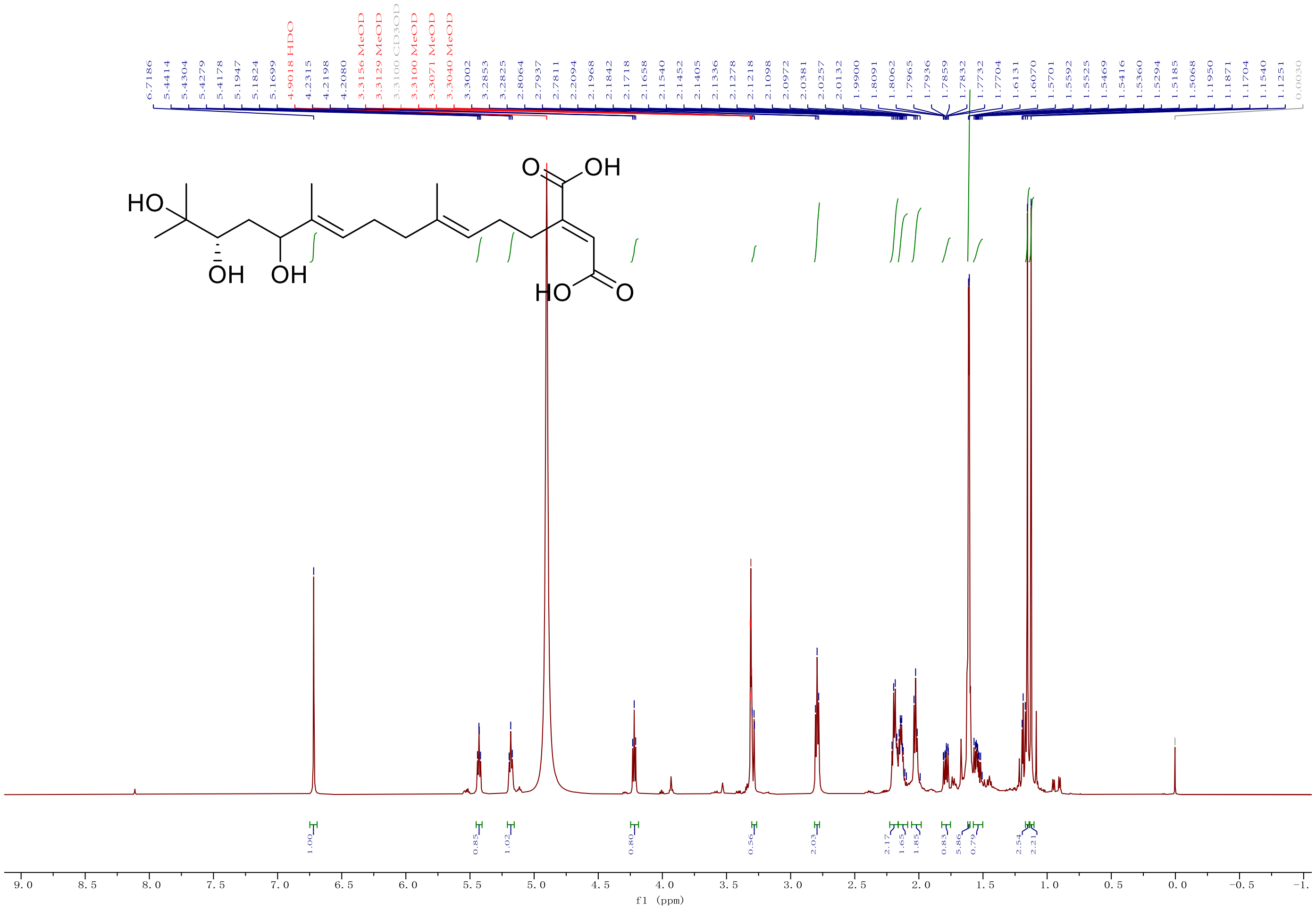

Figure S59. ^{1}H NMR spectrum of compound **8** in MeOH (600 MHz).

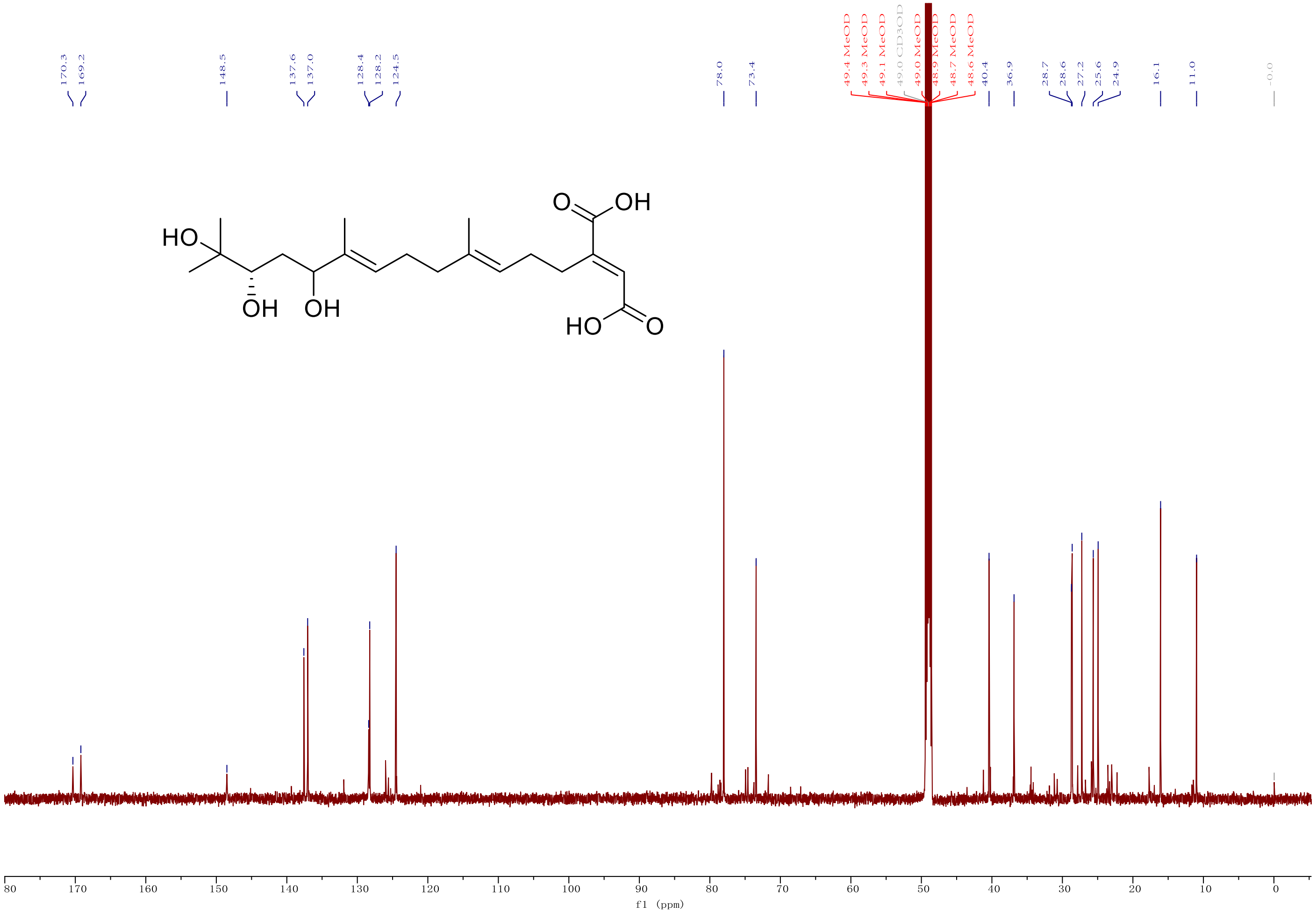

Figure S60. ^{13}C NMR spectrum of compound **8** in MeOH (150 MHz).

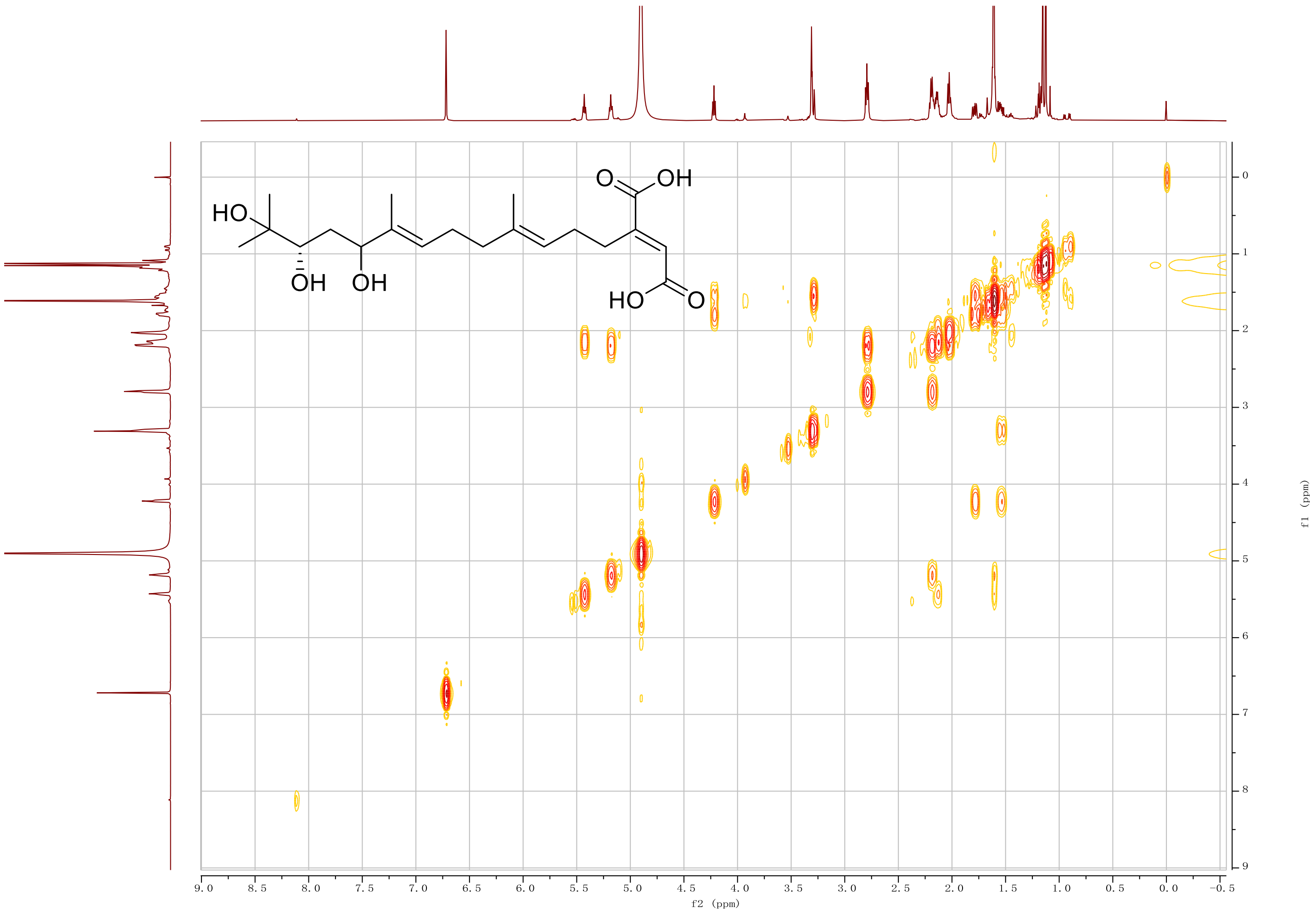

Figure S61. ^{1}H-^{1}H COSY spectrum of compound **8** in MeOH.

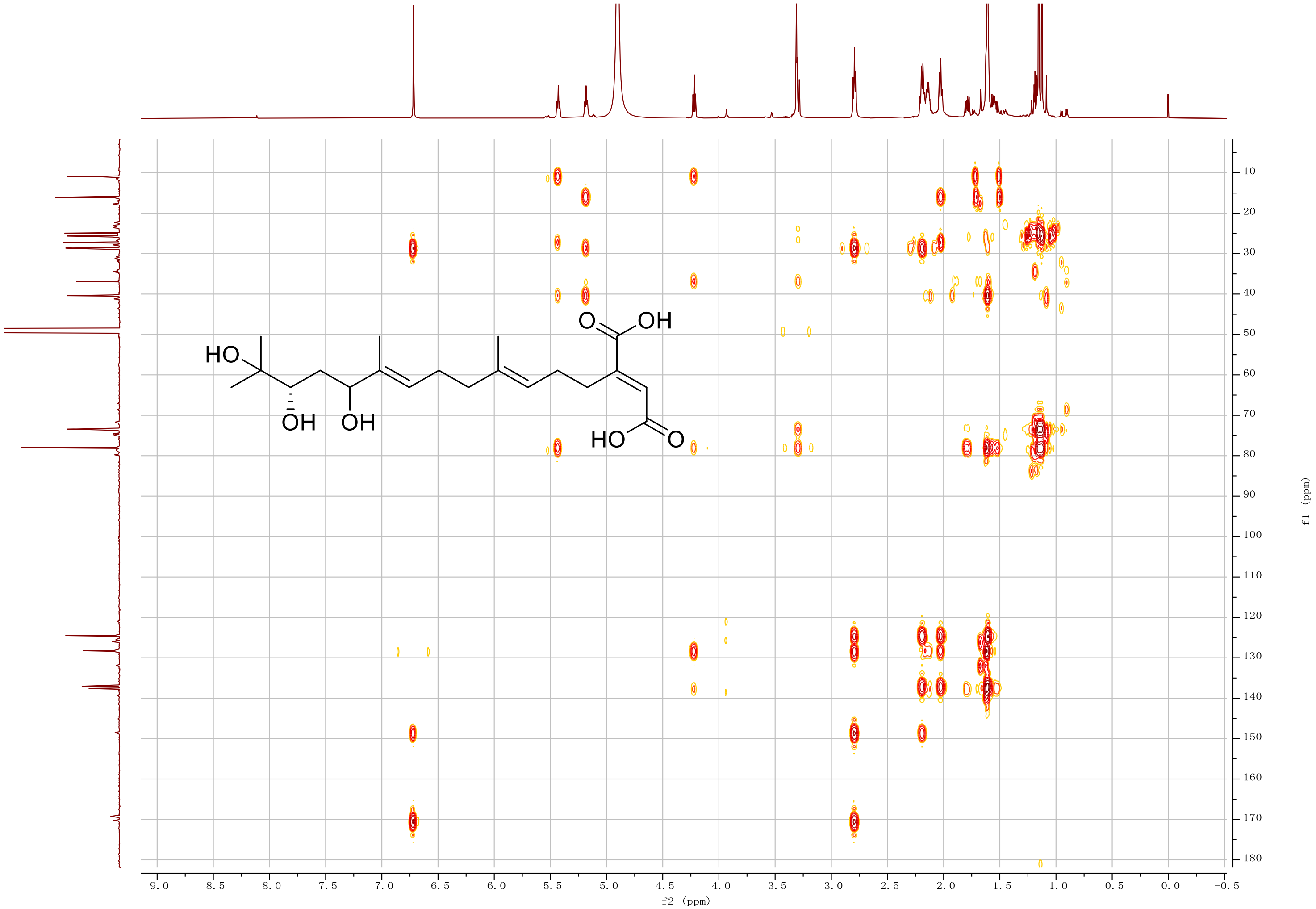

Figure S62. HMBC spectrum of compound **8** in MeOH.

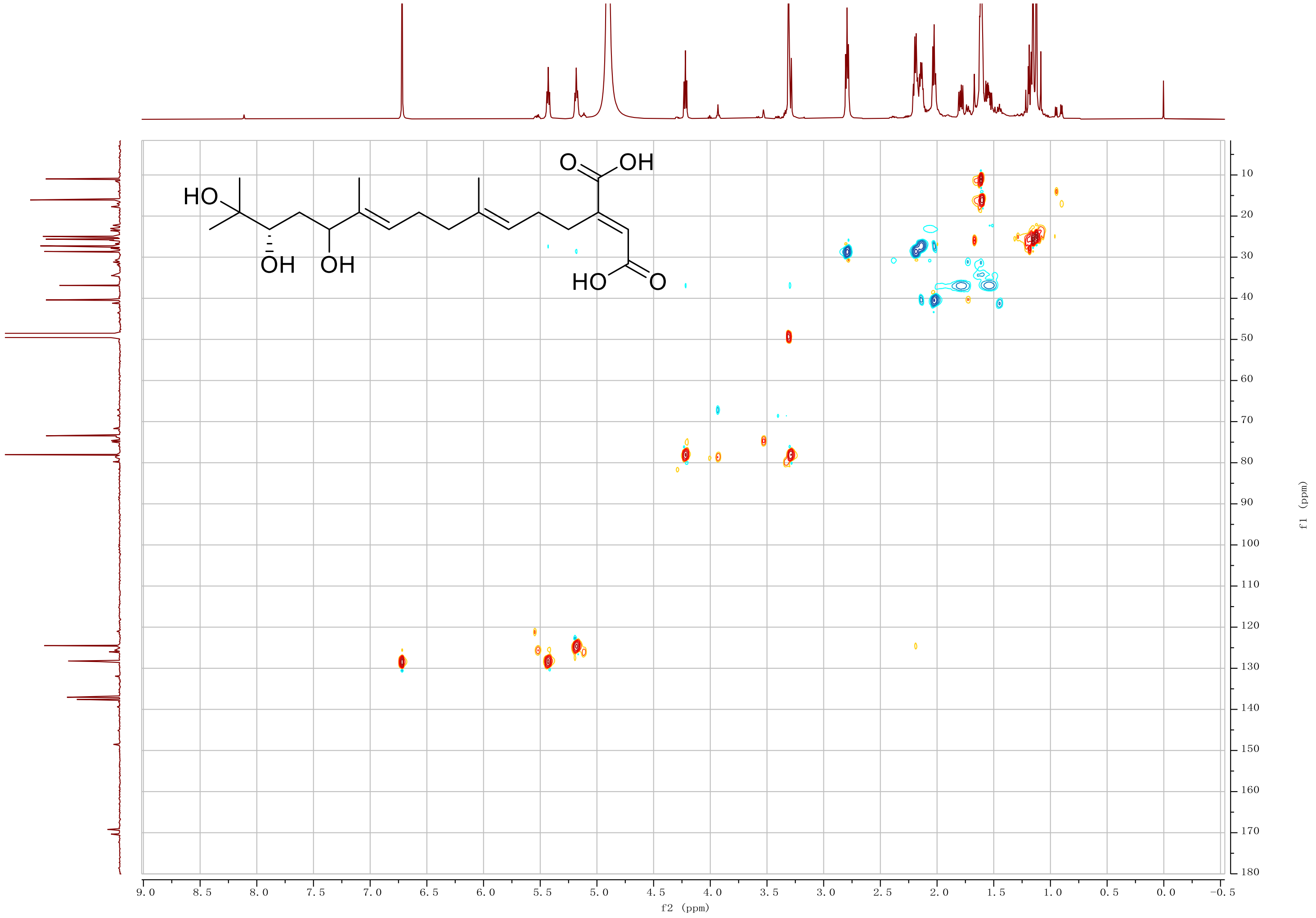

Figure S63. HSQC spectrum of compound **8** in MeOH.

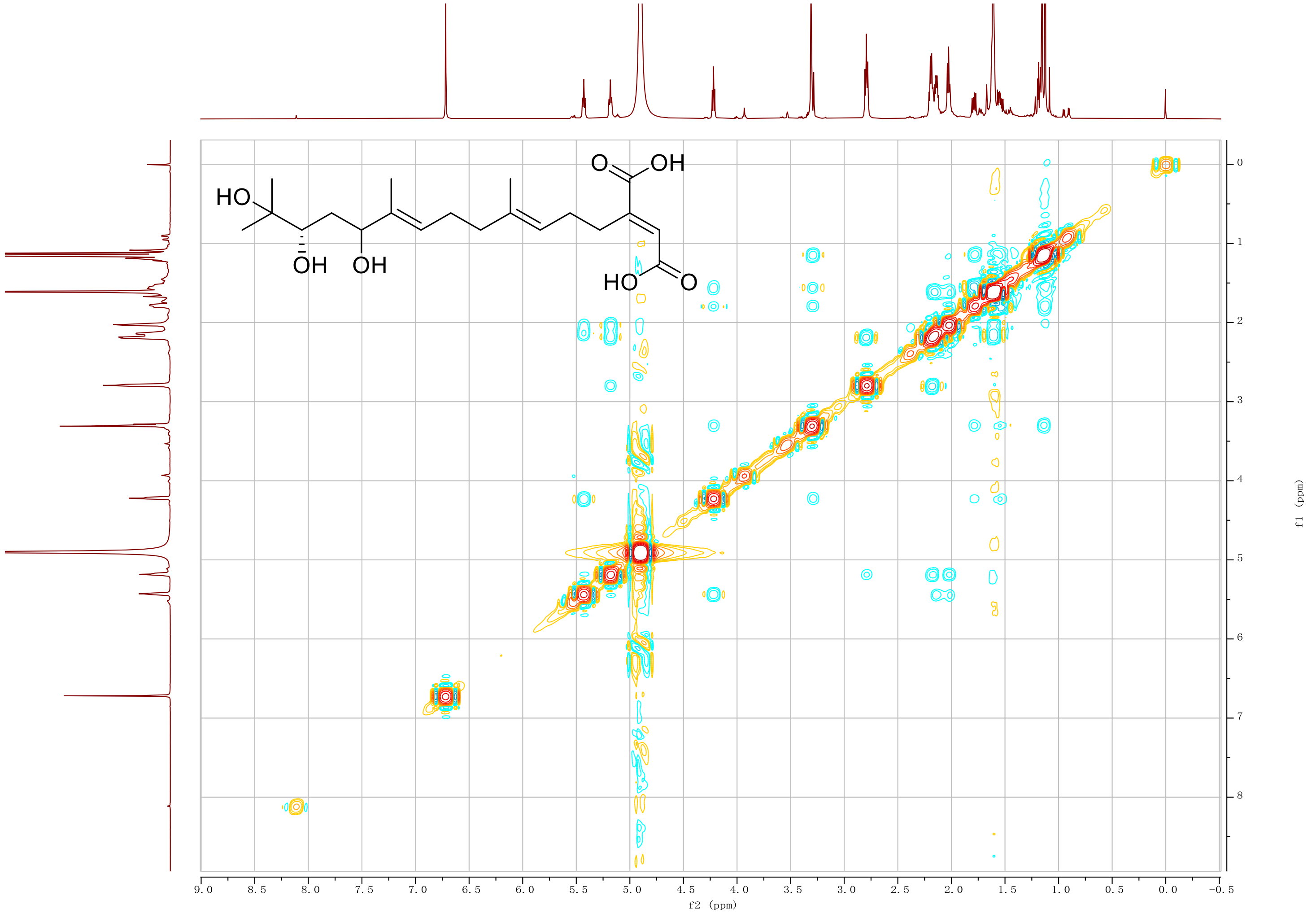

Figure S64. NOESY spectrum of compound **8** in MeOH.

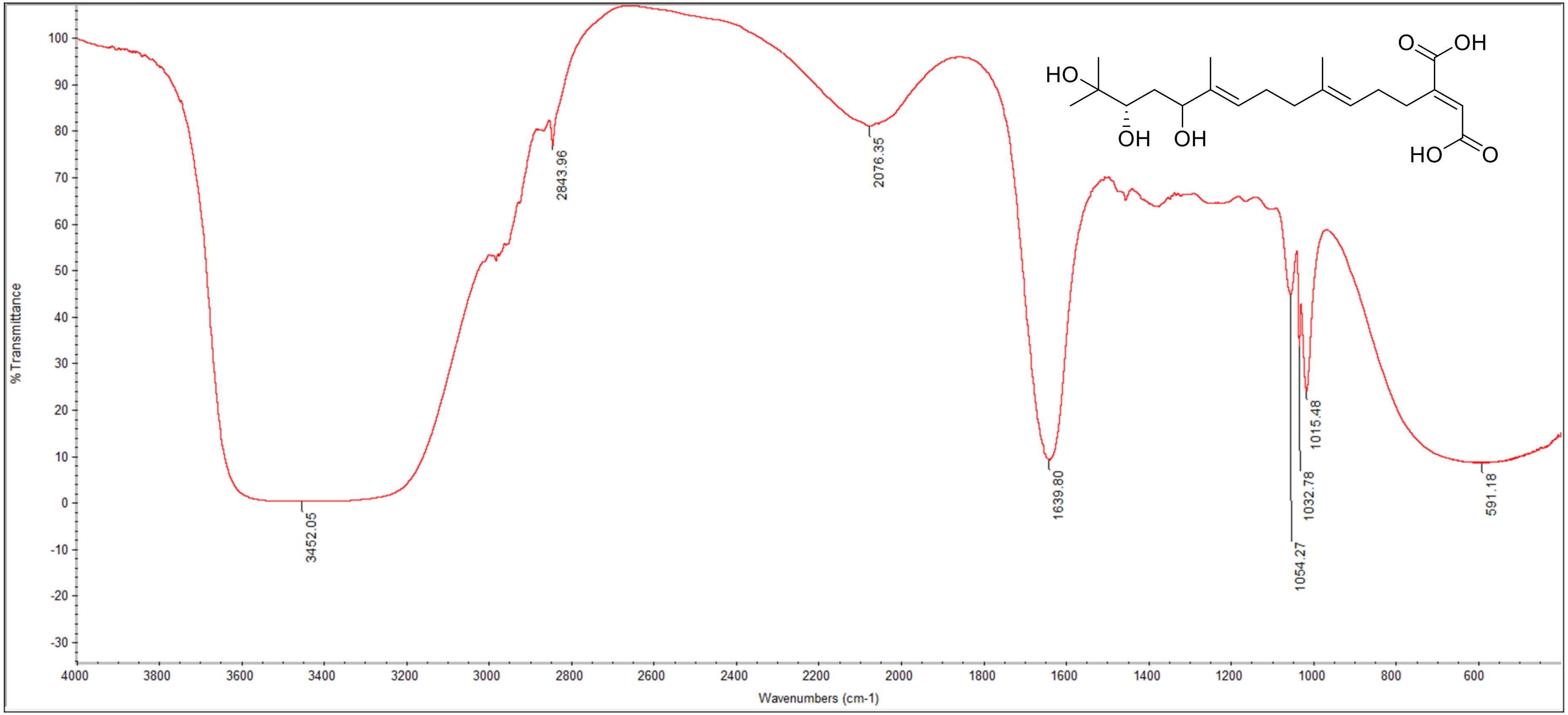

Figure S65. IR spectrum of compound **8** in MeOH.

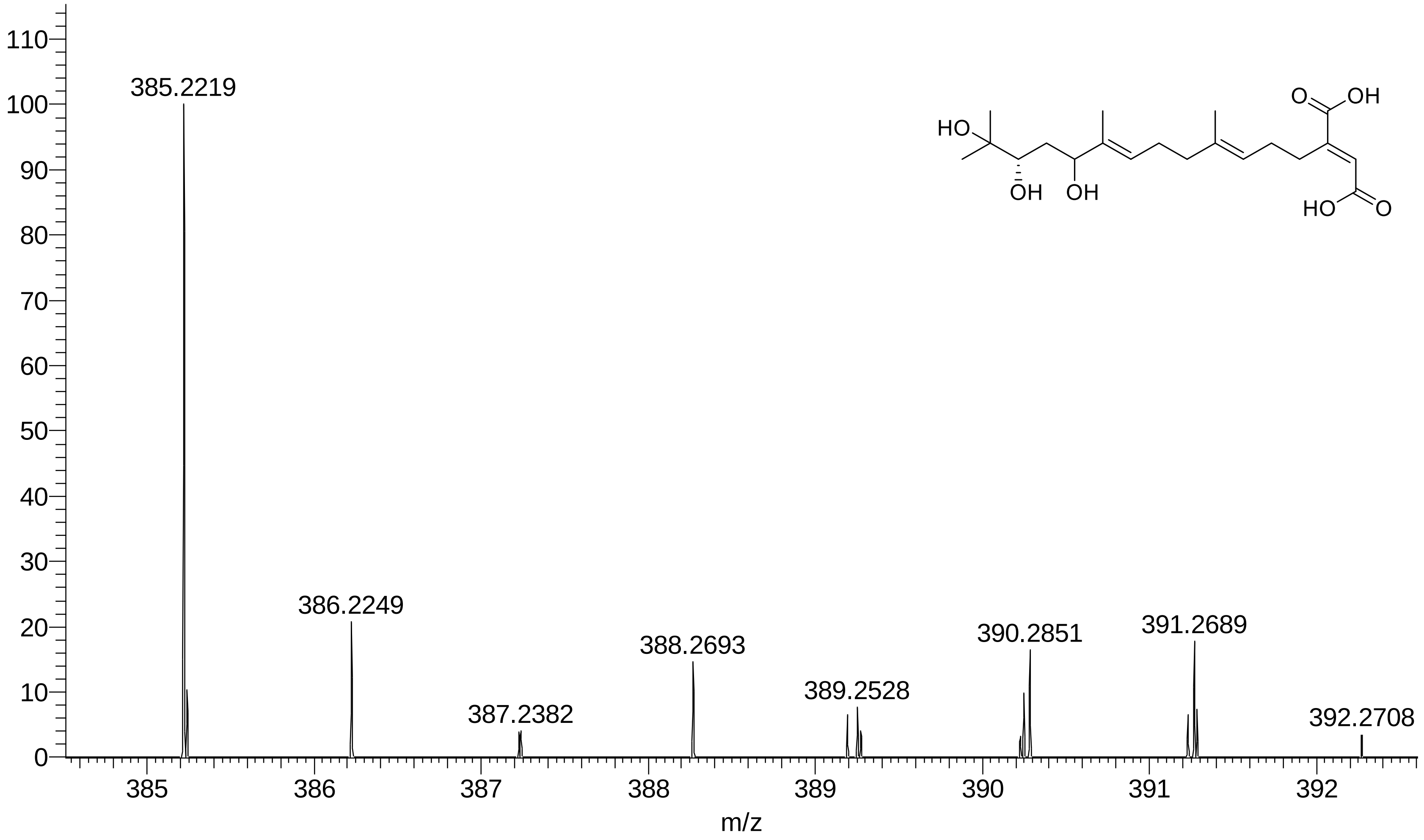

Figure S66. HR-ESI-MS spectrum of compound **8** in MeOH.

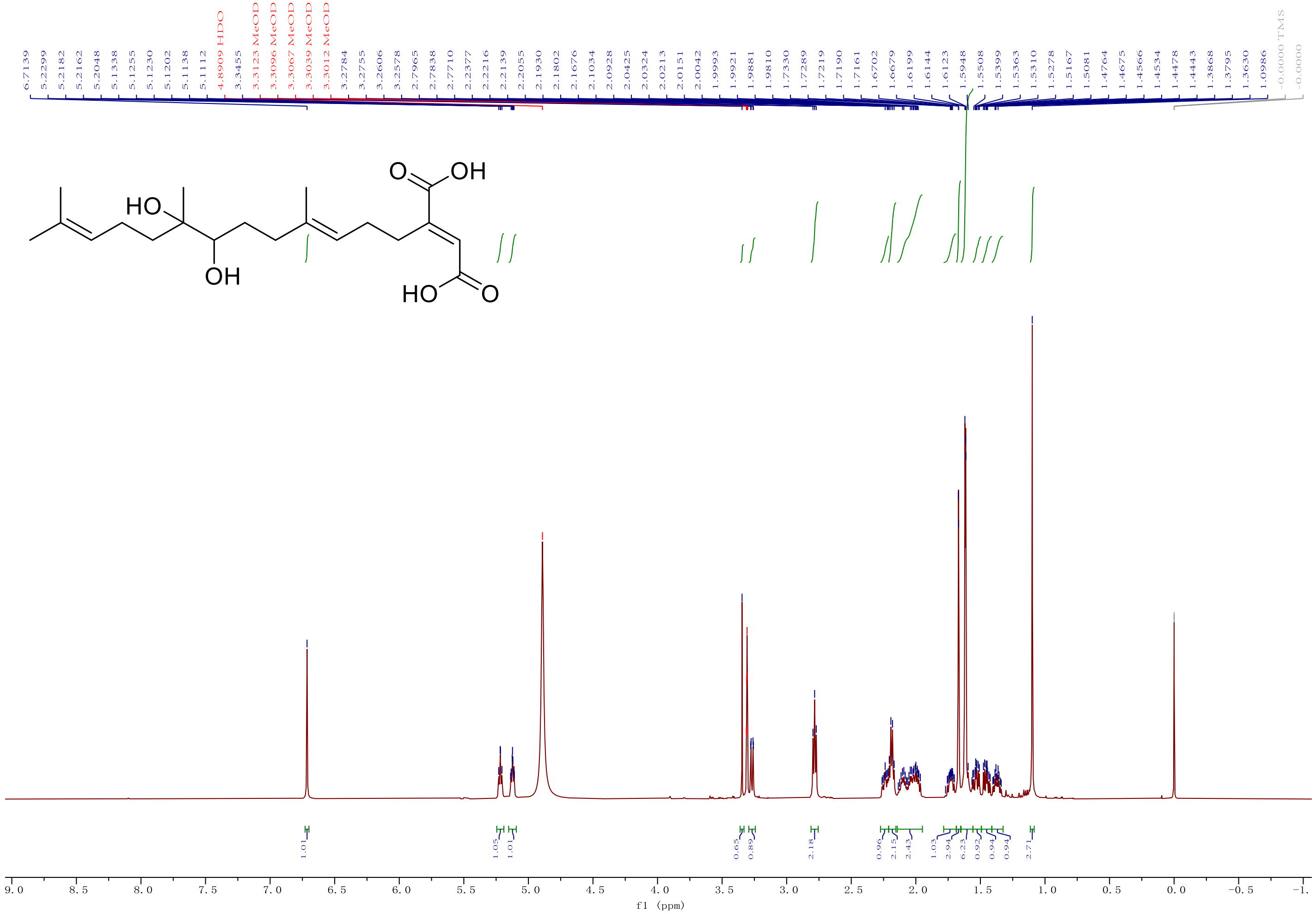

Figure S67. ^{1}H NMR spectrum of compound **9** in MeOH (600 MHz).

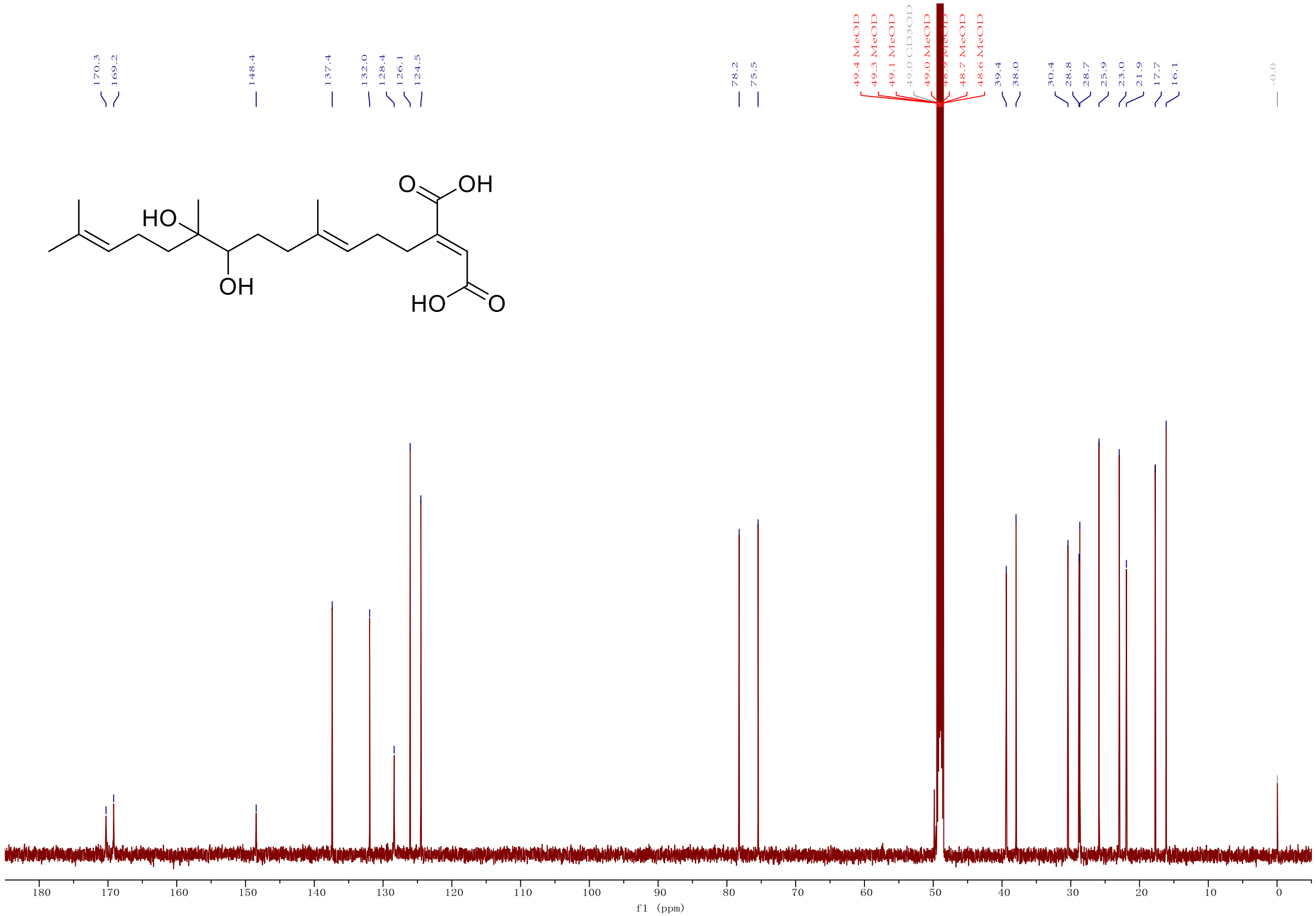

Figure S68. ^{13}C NMR spectrum of compound **9** in MeOH (150 MHz).

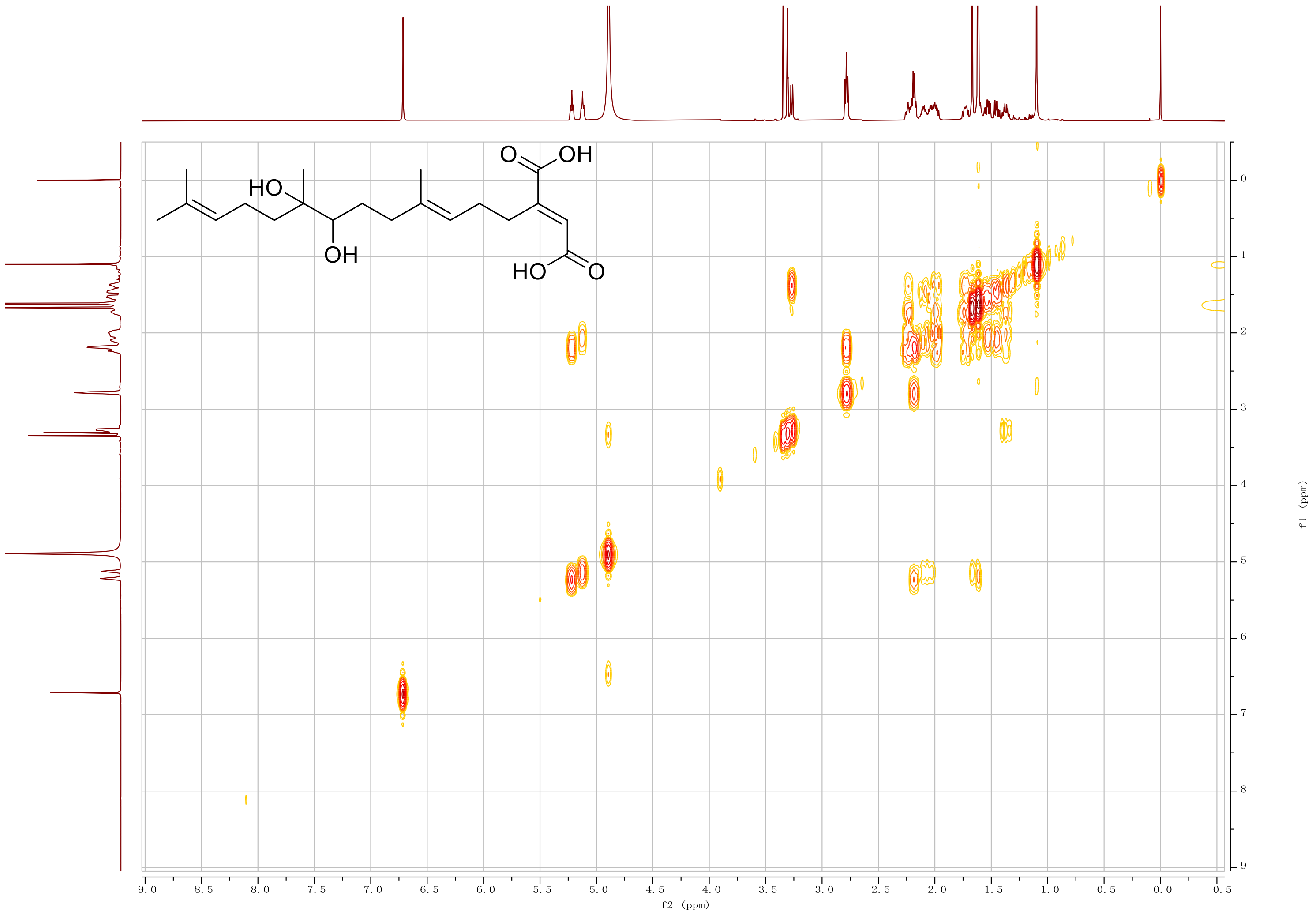

Figure S69. ^1H-^1H COSY spectrum of compound **9** in MeOH.

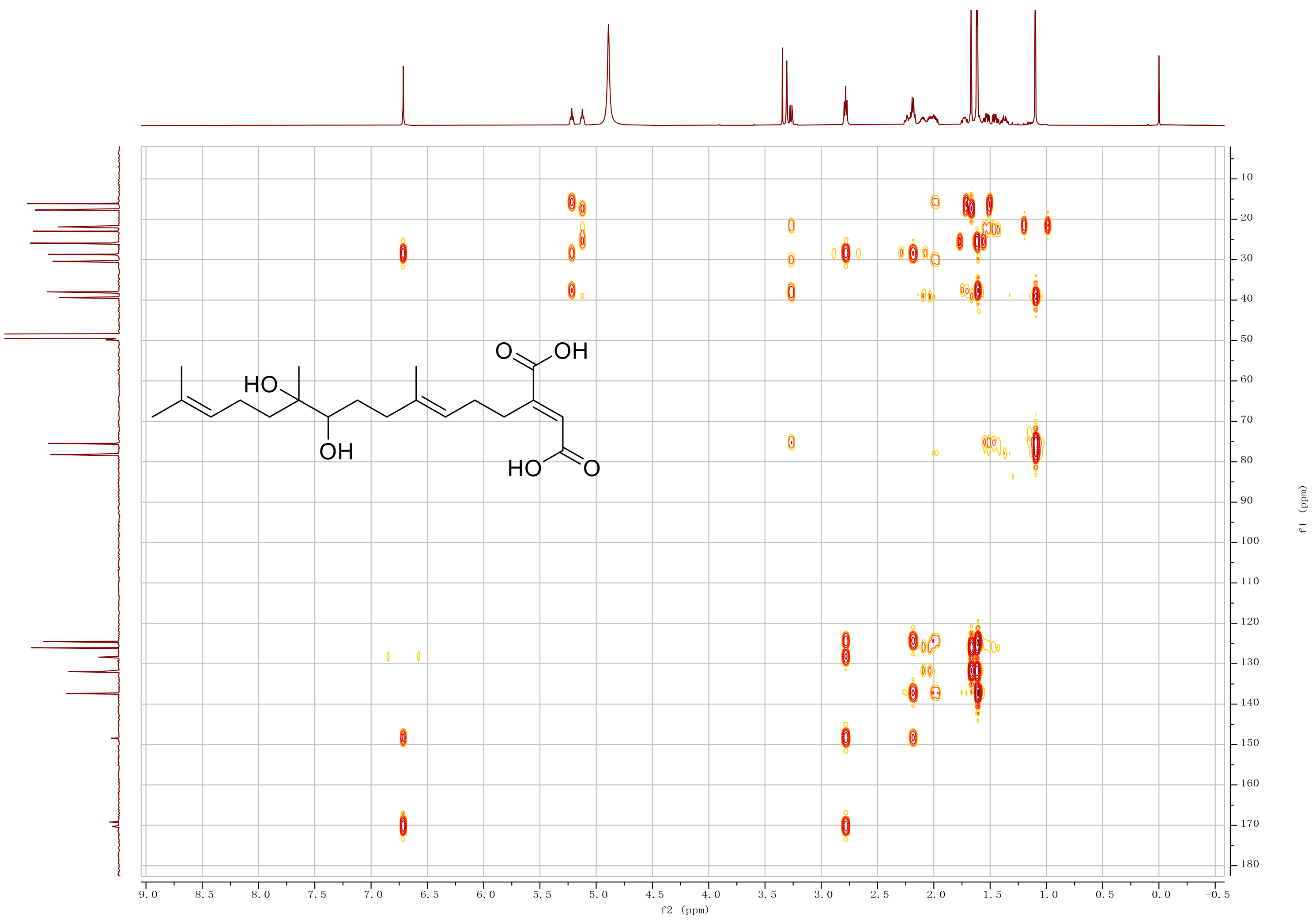

Figure S70. HMBC spectrum of compound **9** in MeOH.

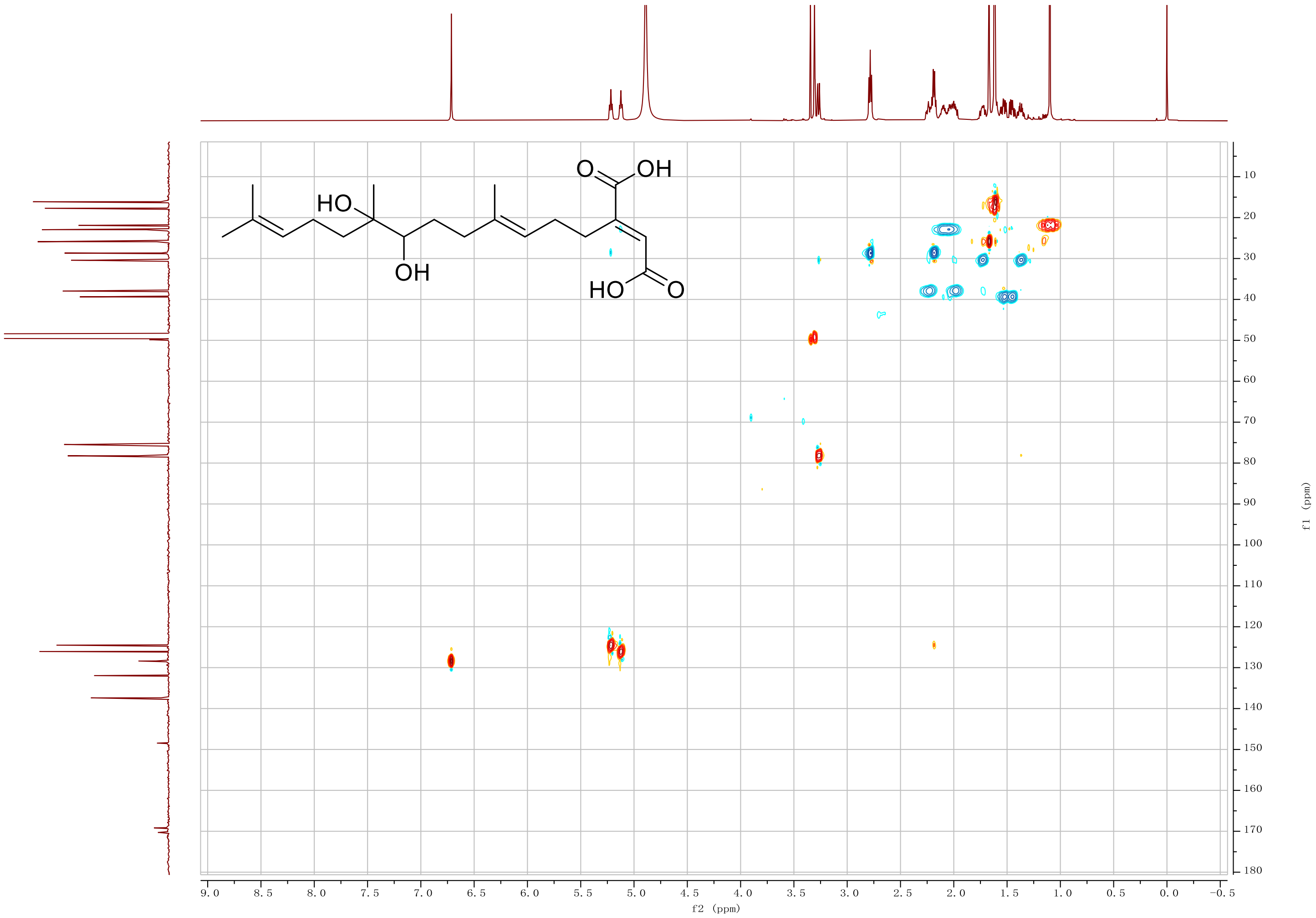

Figure S71. HSQC spectrum of compound **9** in MeOH.

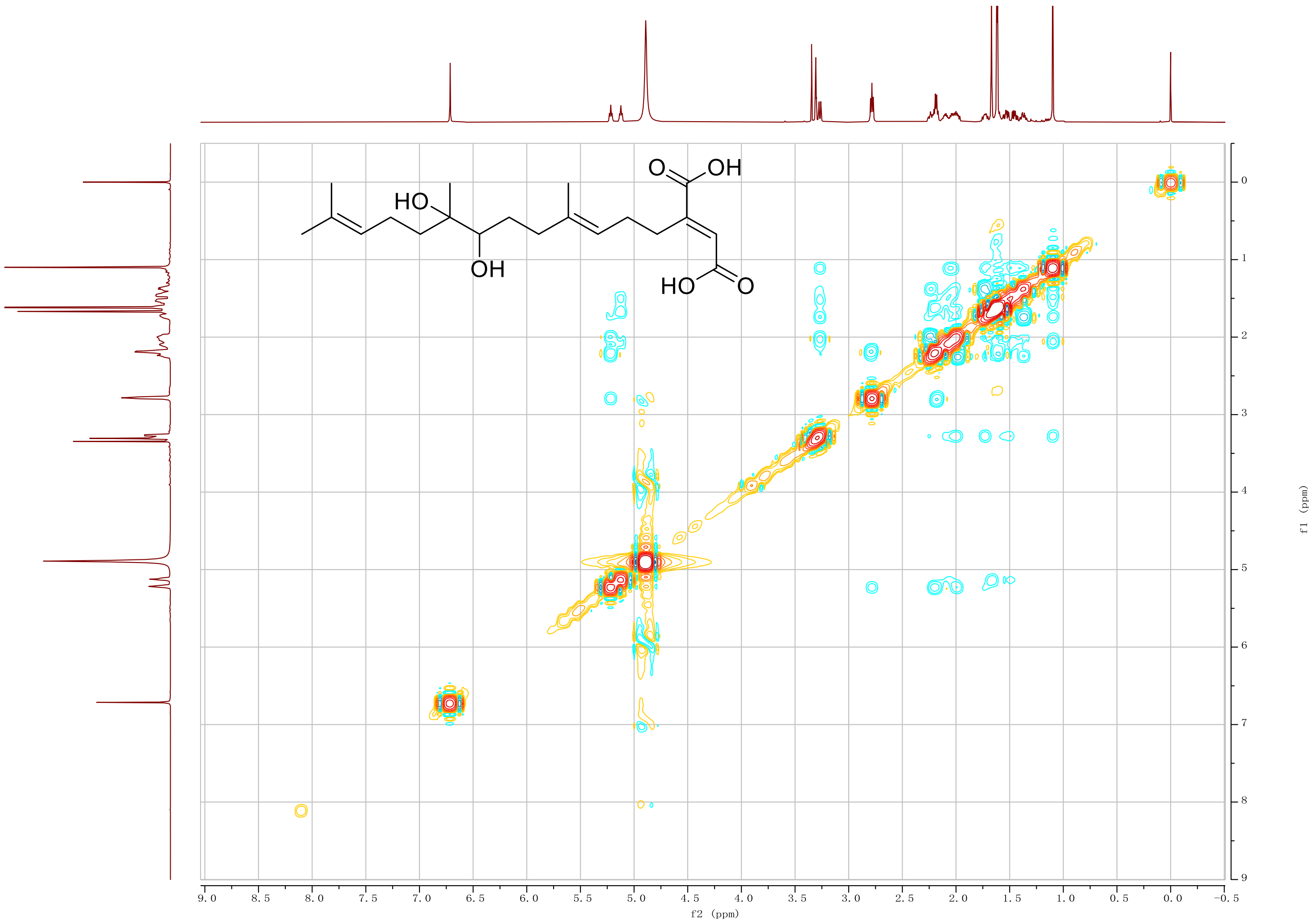

Figure S72. NOESY spectrum of compound **9** in MeOH.

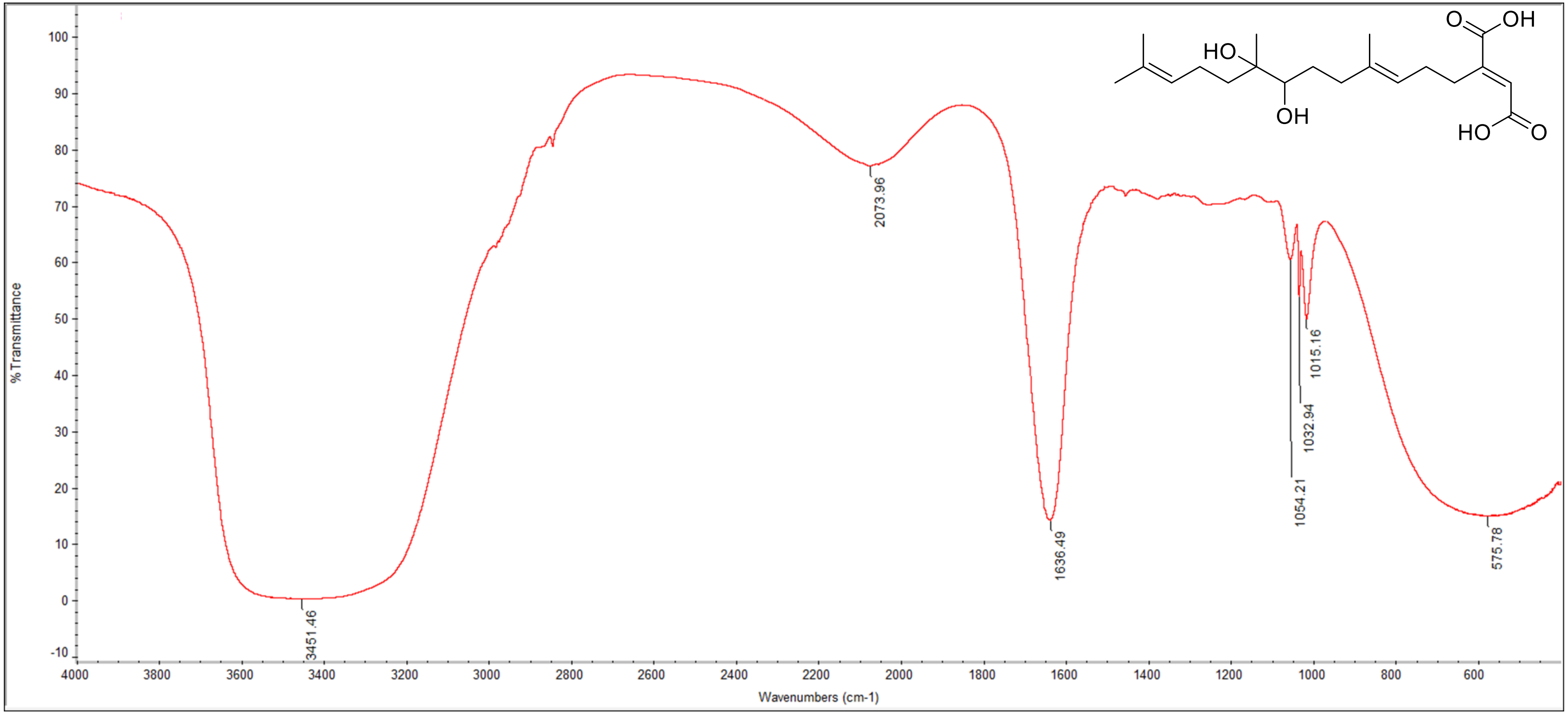

Figure S73. IR spectrum of compound **9** in MeOH.

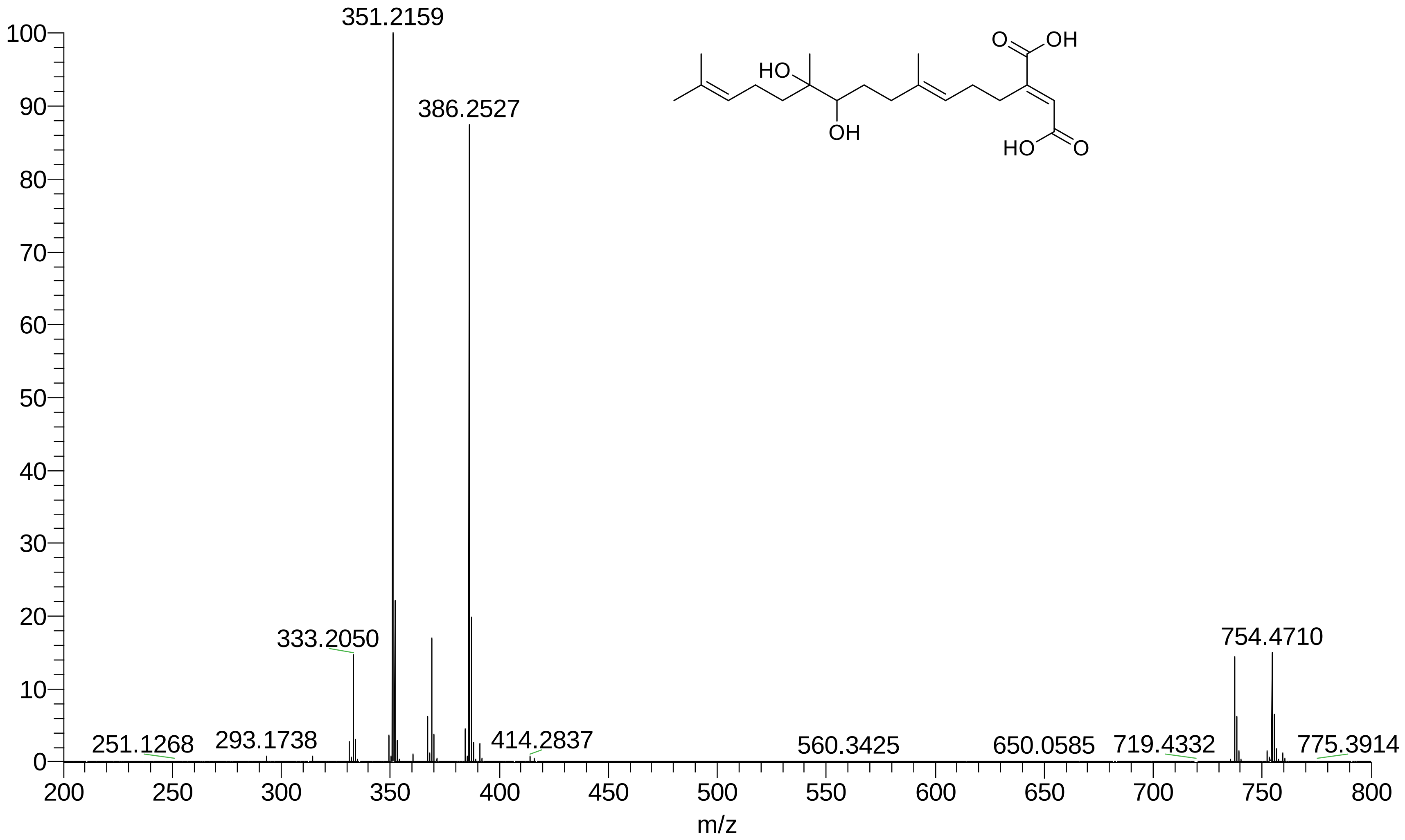

Figure S74. HR-ESI-MS spectrum of compound **9** in MeOH.

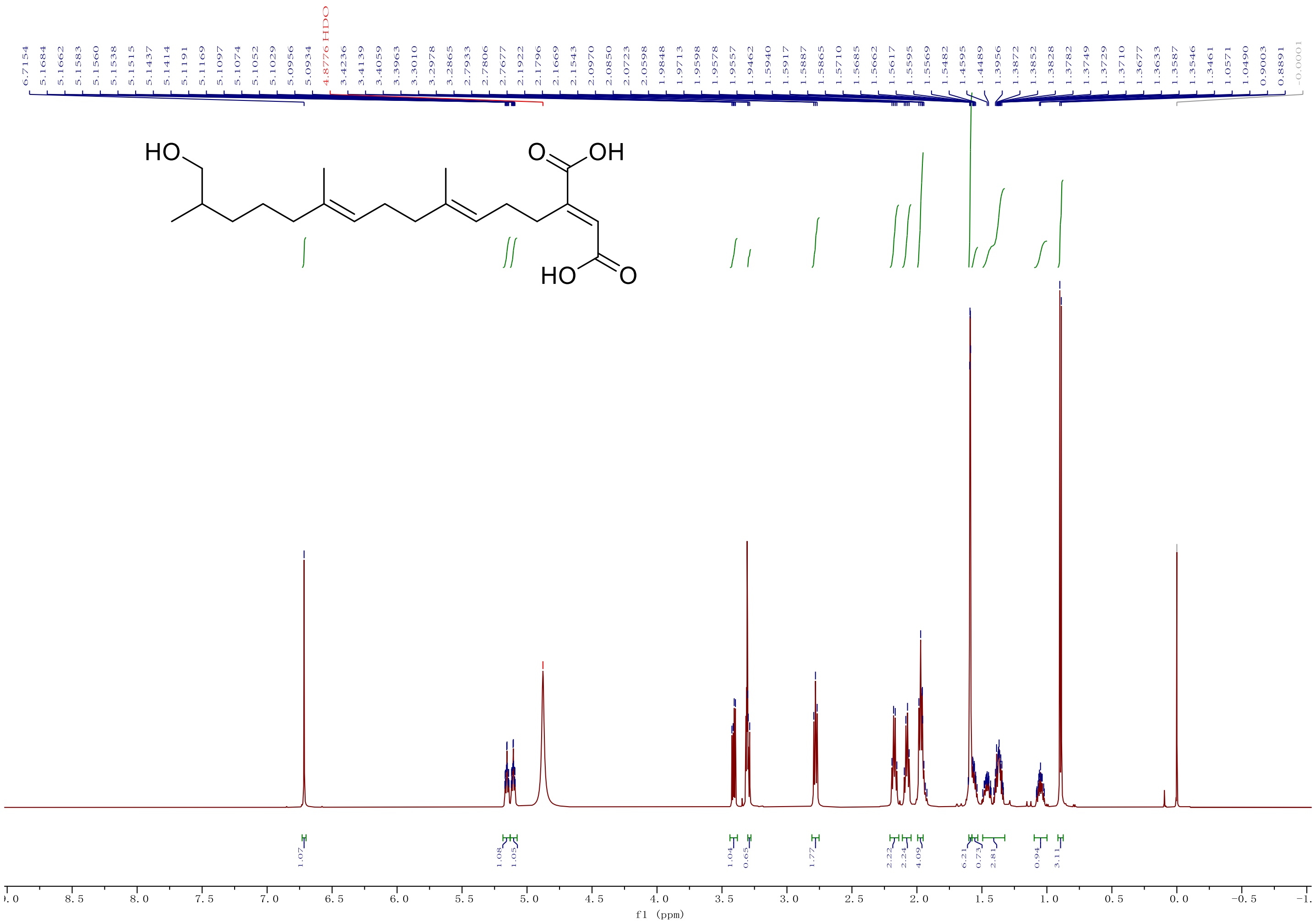

Figure S75. ^{1}H NMR spectrum of compound **10** in MeOH (600 MHz).

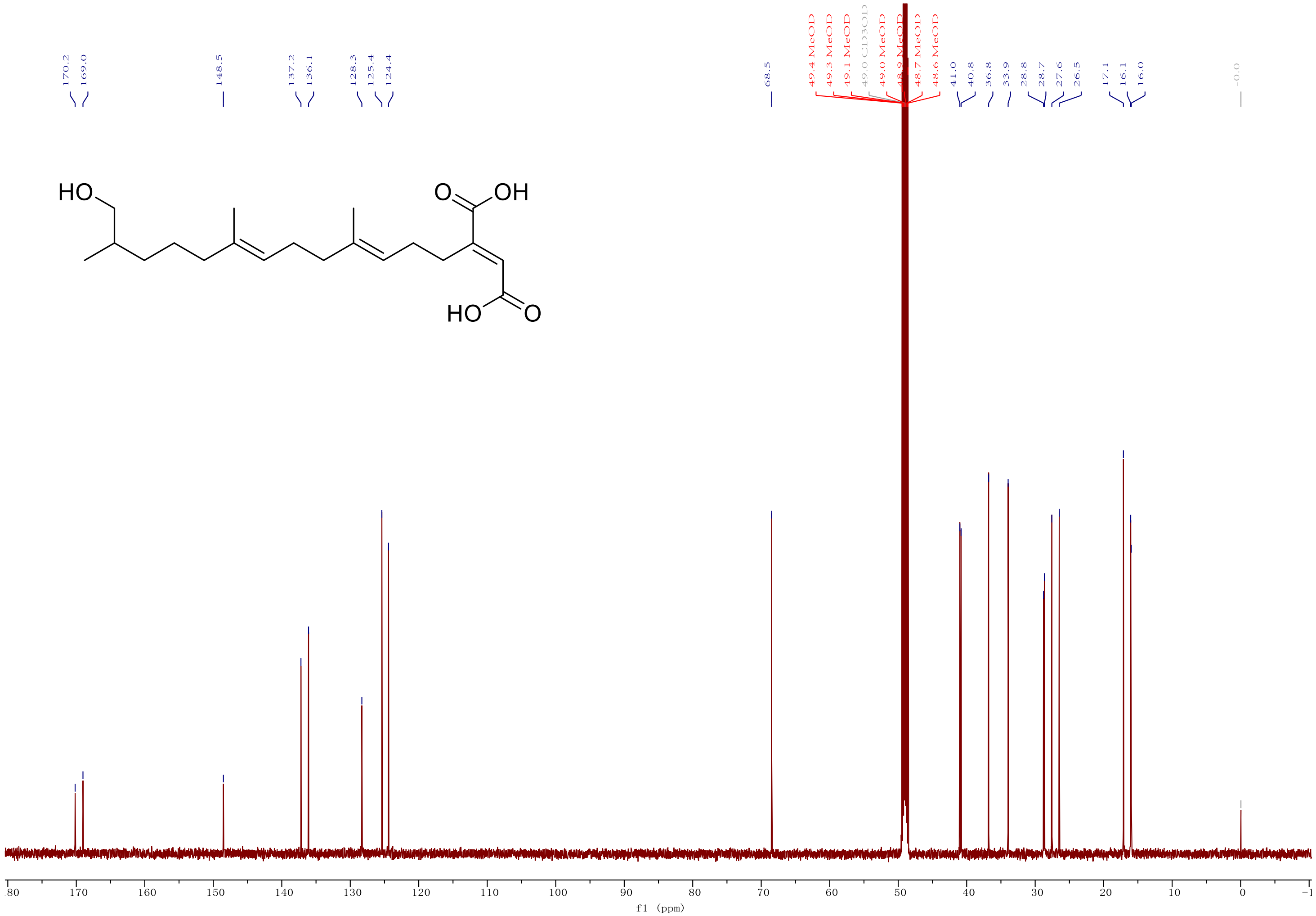

Figure S76. ^{13}C NMR spectrum of compound **10** in MeOH (150 MHz).

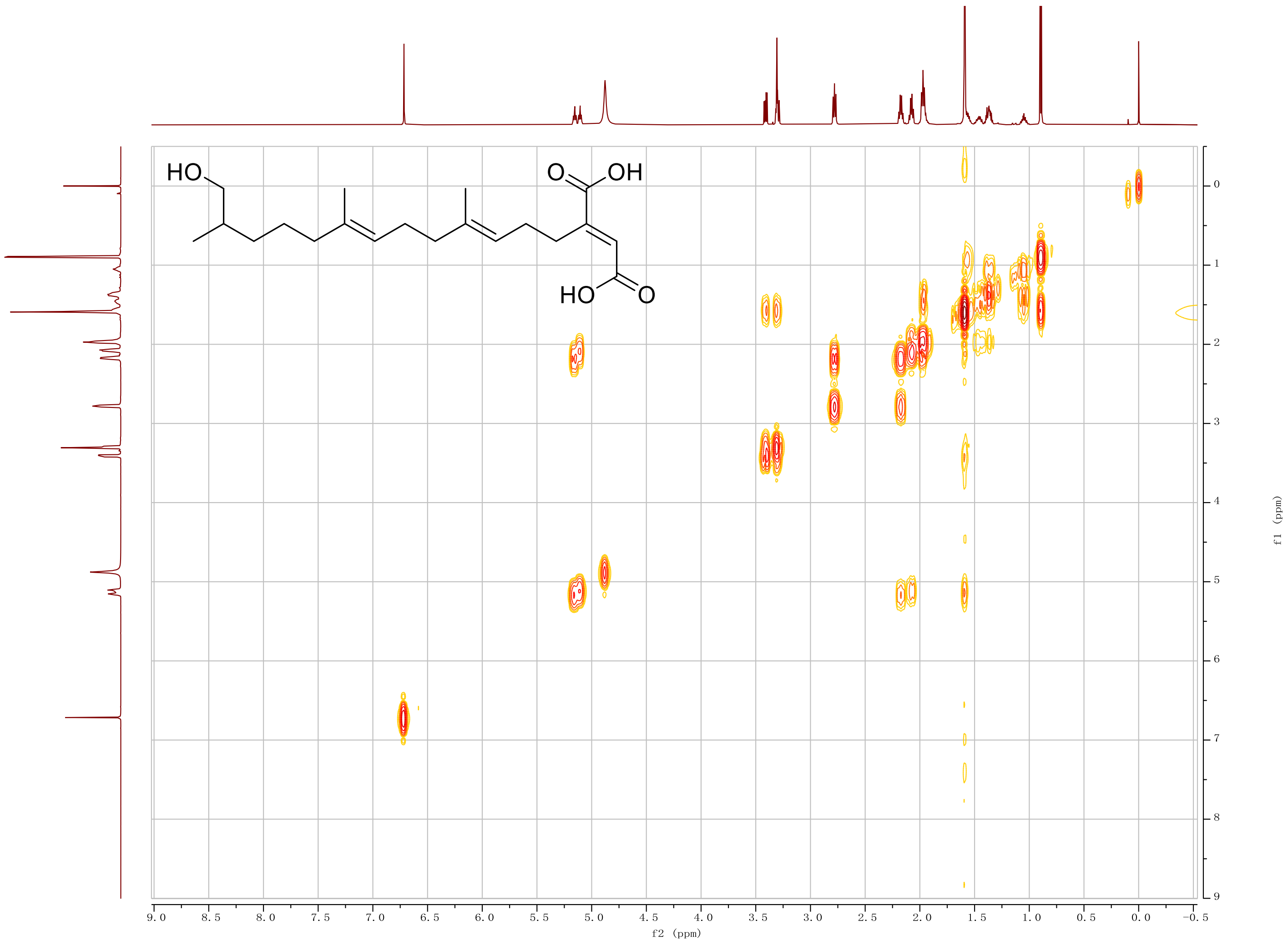

Figure S77. ^{1}H-^{1}H COSY spectrum of compound **10** in MeOH.

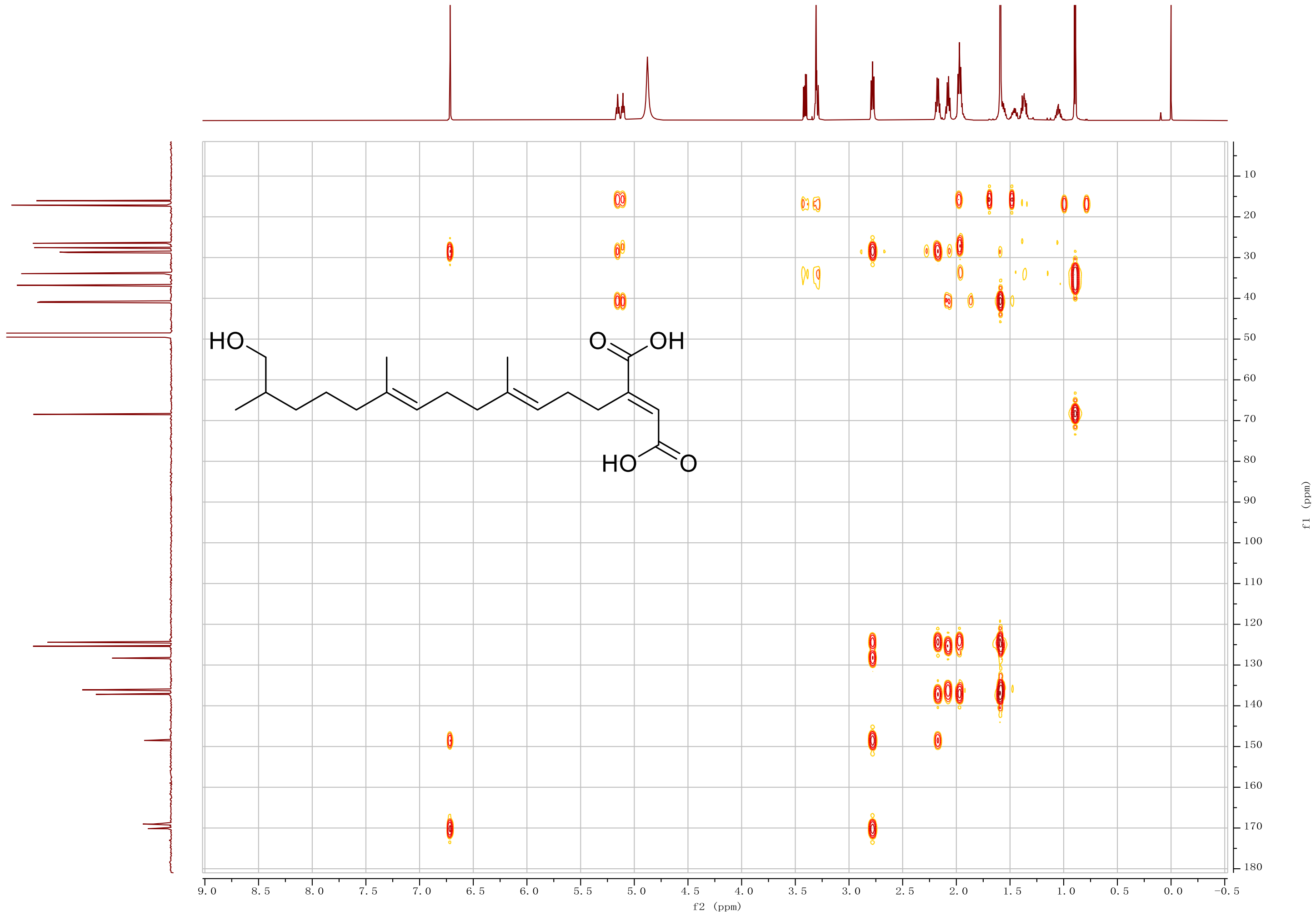

Figure S78. HMBC spectrum of compound **10** in MeOH.

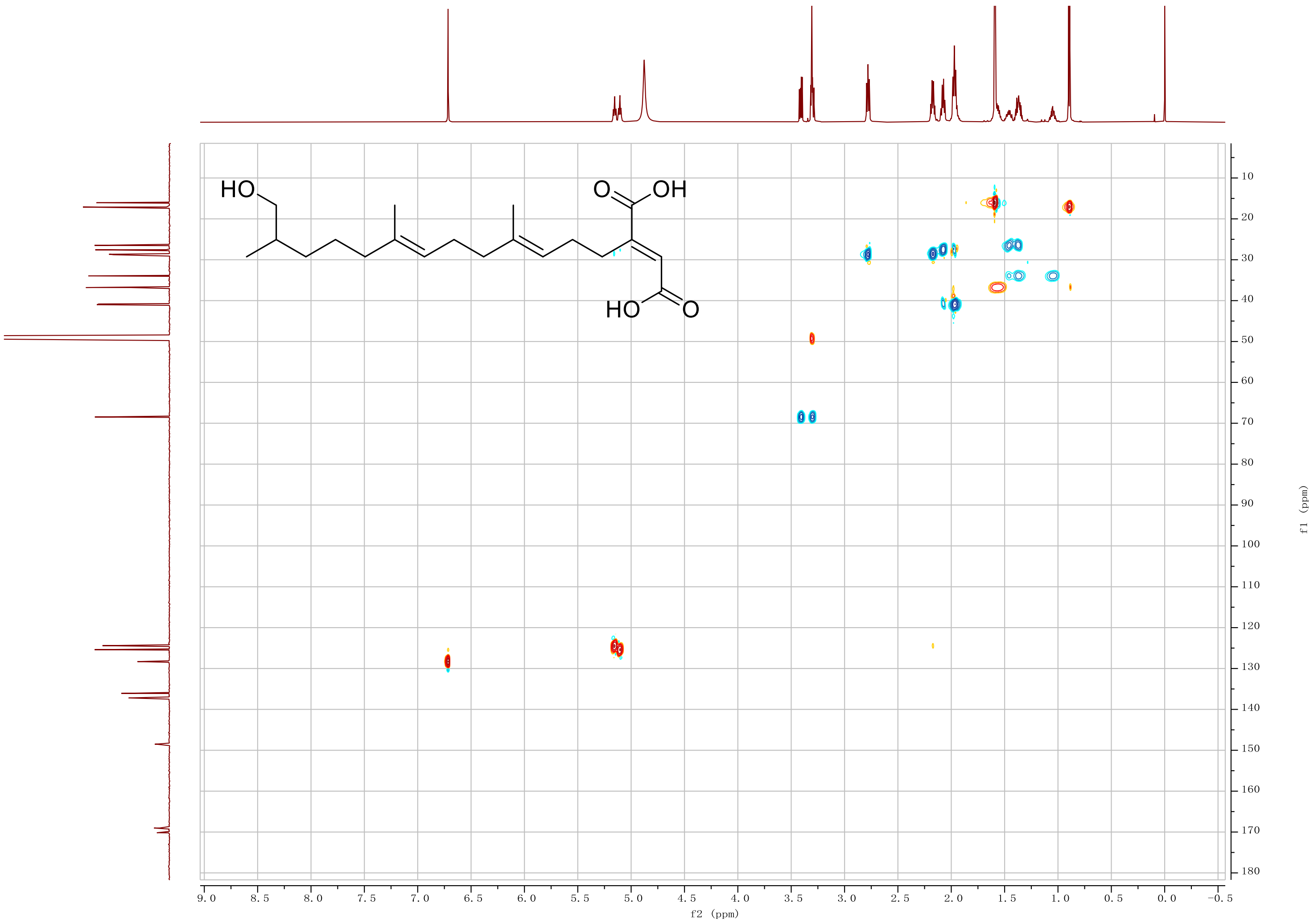

Figure S79. HSQC spectrum of compound **10** in MeOH.

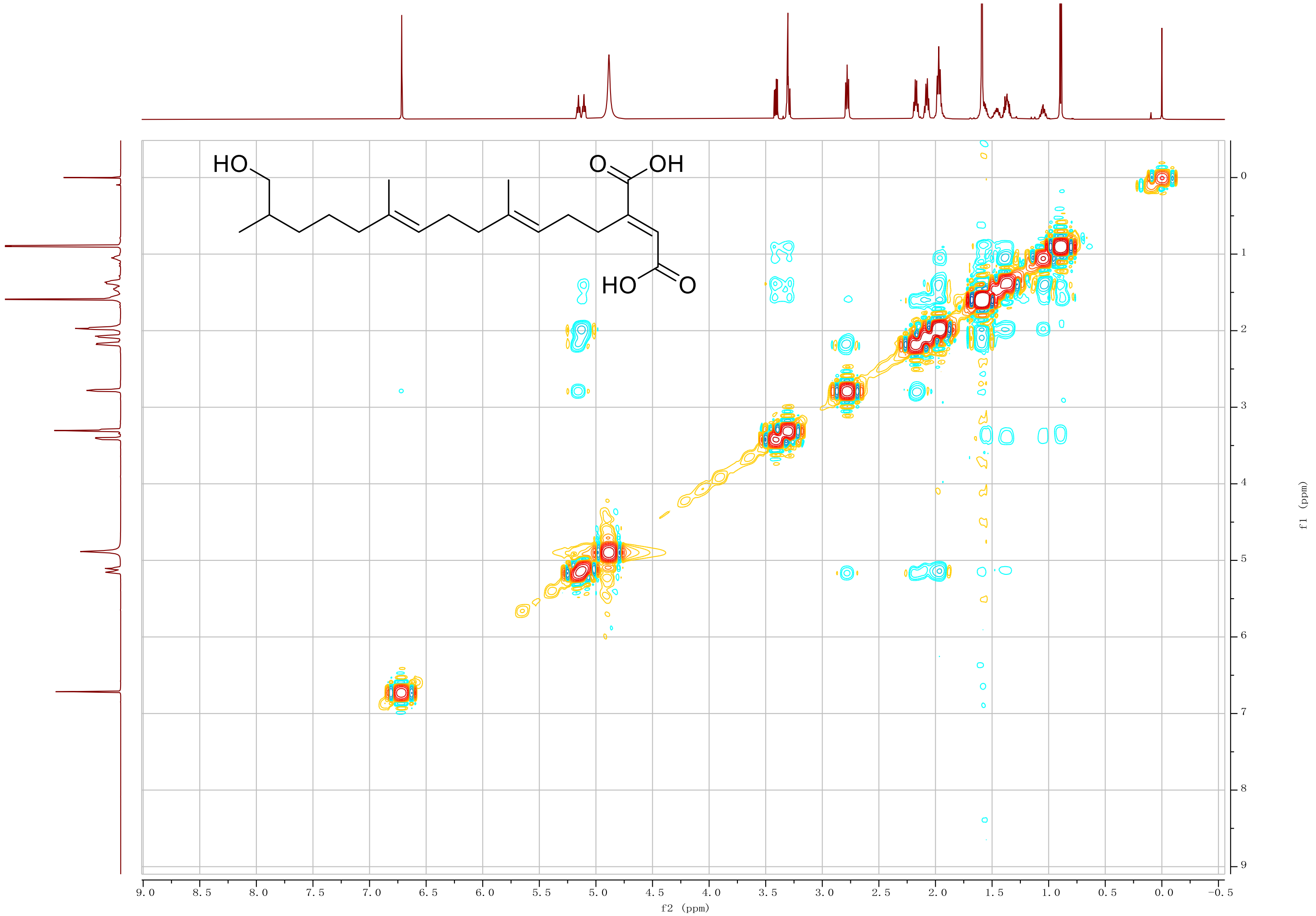

Figure S80. NOESY spectrum of compound **10** in MeOH.

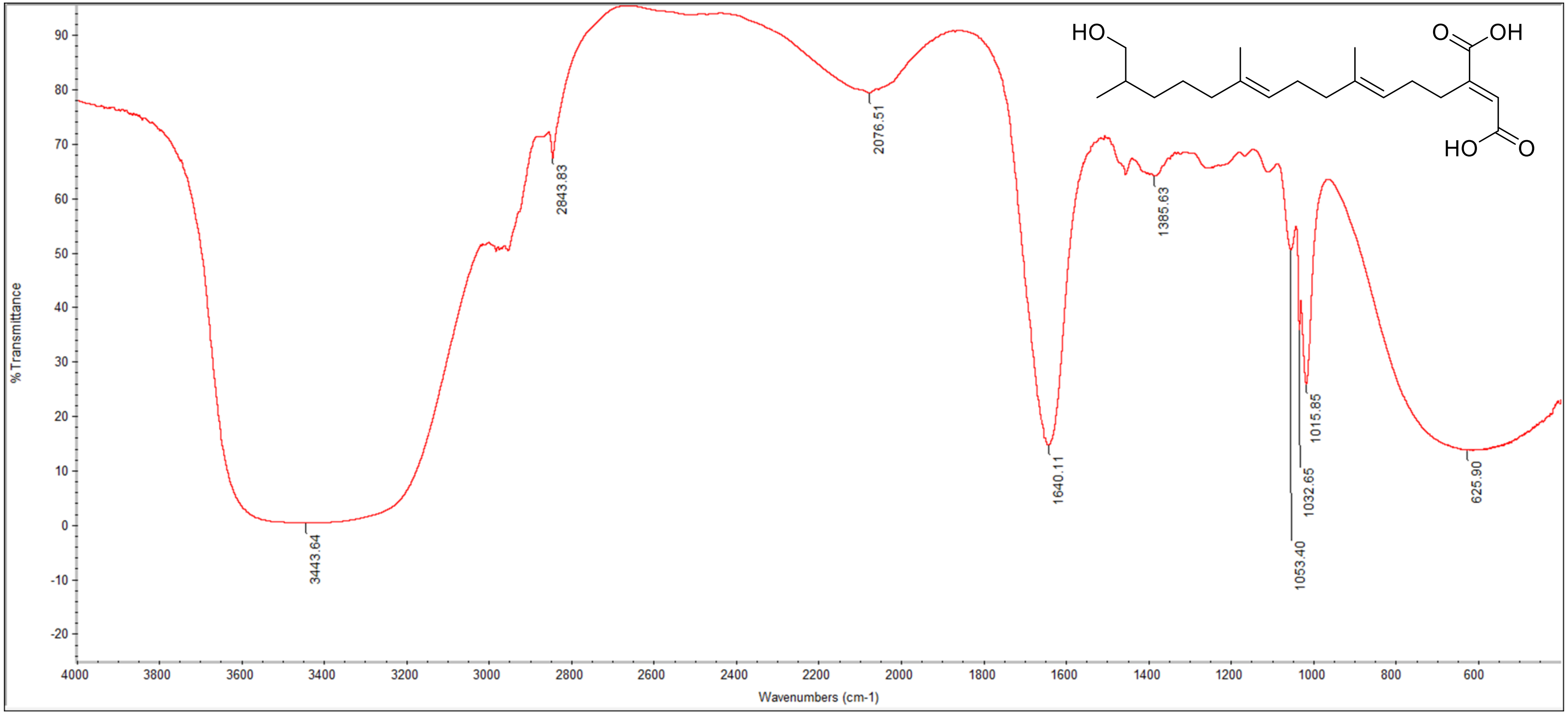

Figure S81. IR spectrum of compound **10** in MeOH.

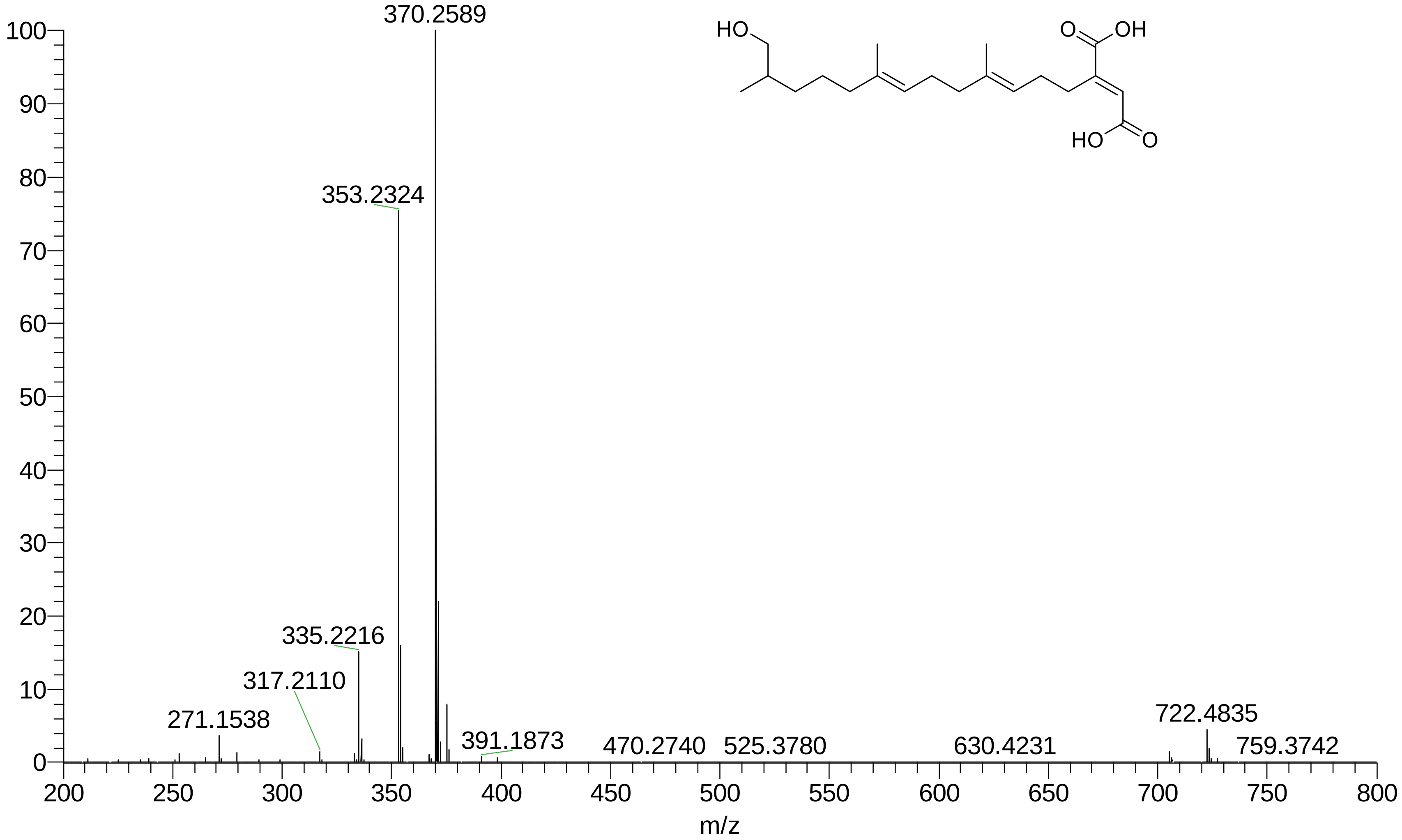

Figure S82. HR-ESI-MS spectrum of compound **10** in MeOH.

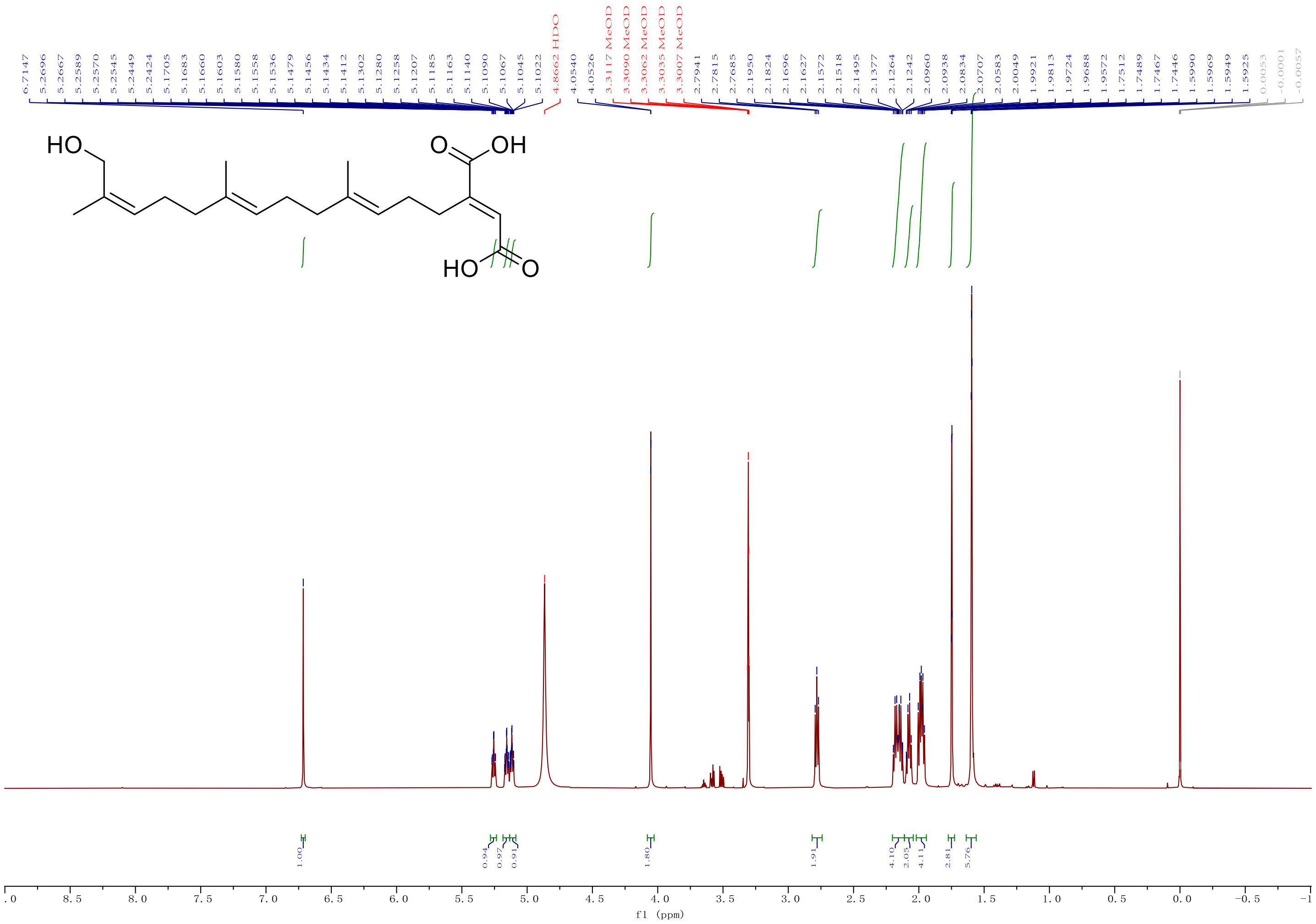

Figure S83. ^{1}H NMR spectrum of compound **11** in MeOH (600 MHz).

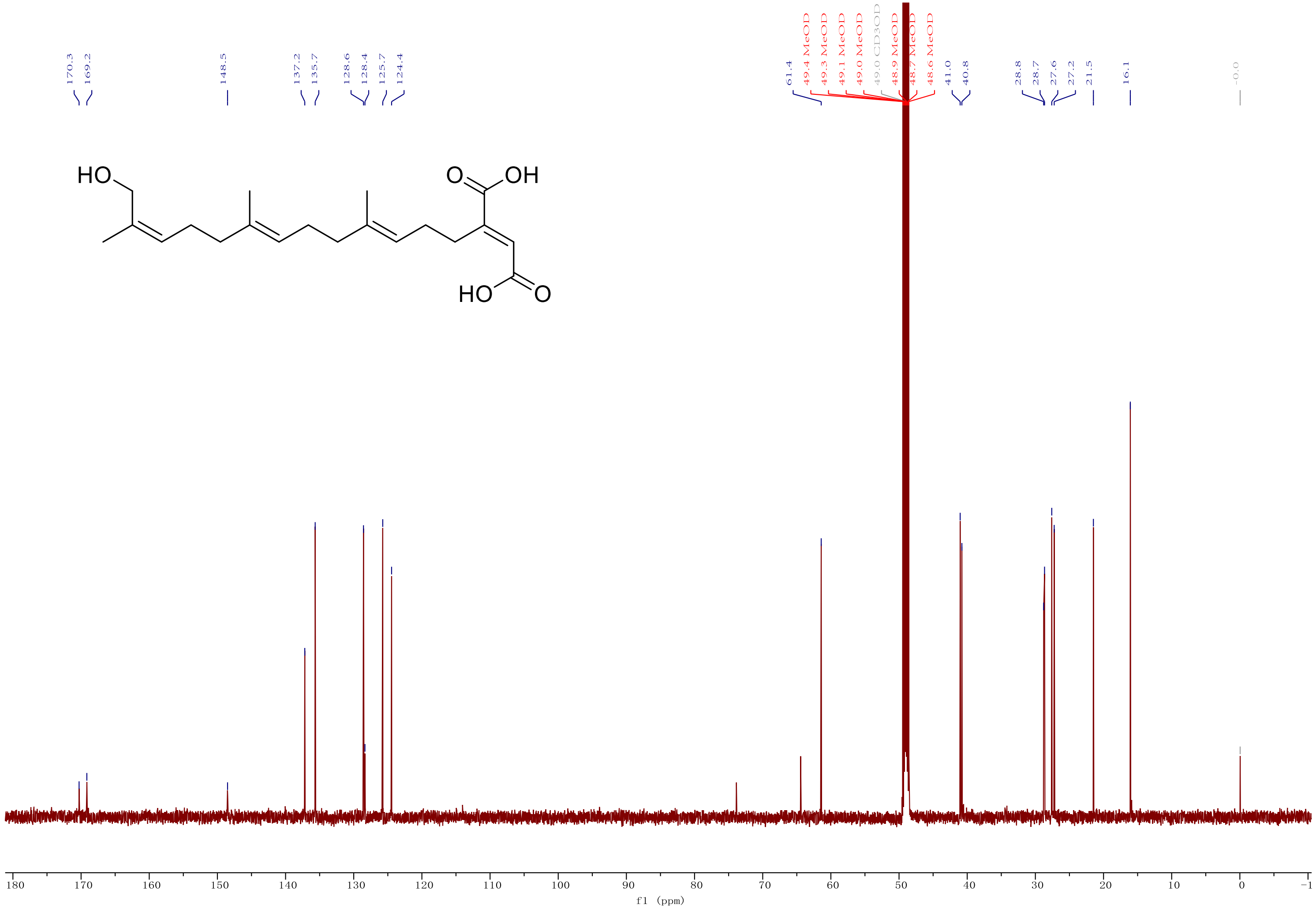

Figure S84. ^{13}C NMR spectrum of compound **11** in MeOH (150 MHz).

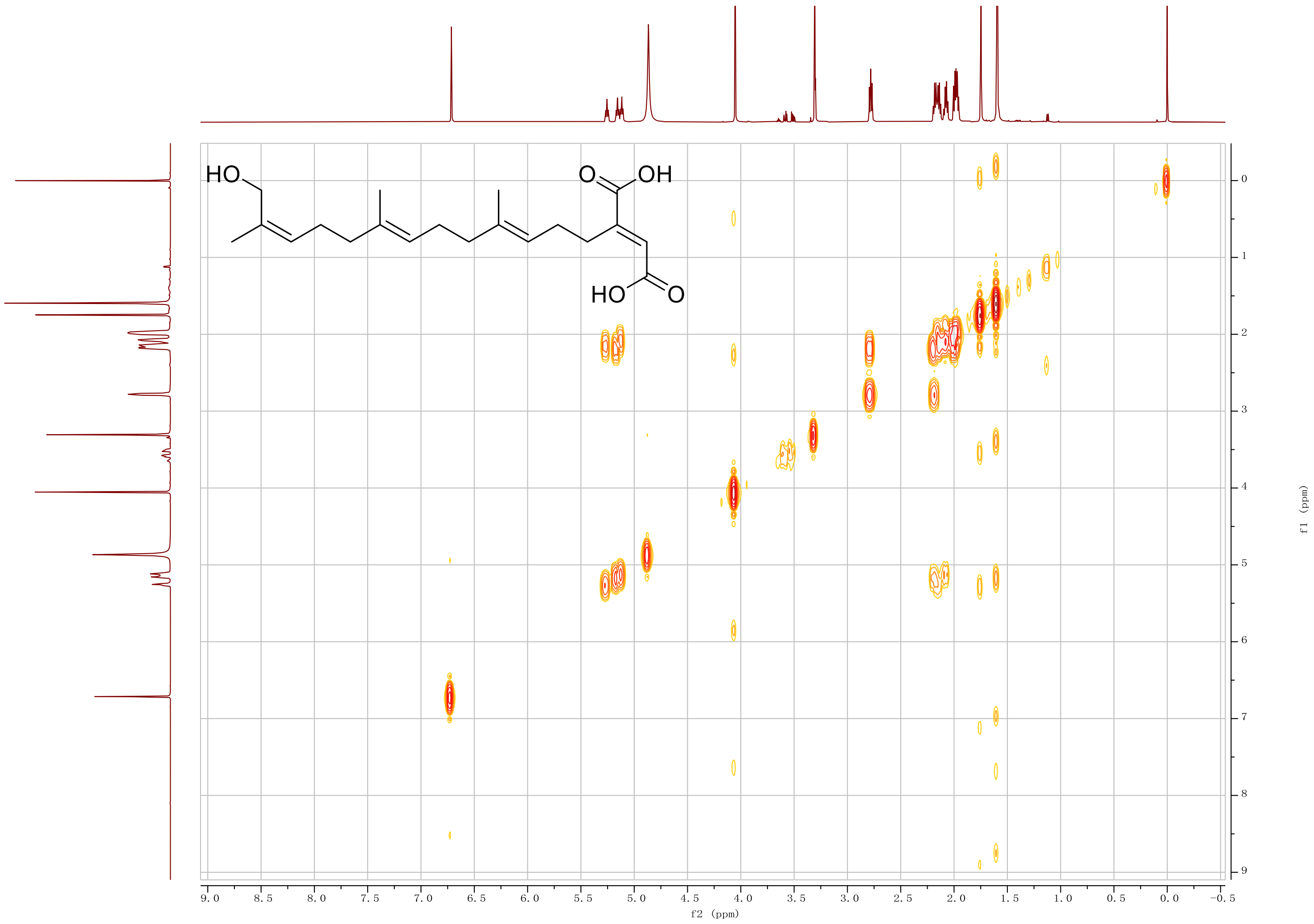

Figure S85. ^{1}H-^{1}H COSY spectrum of compound **11** in MeOH.

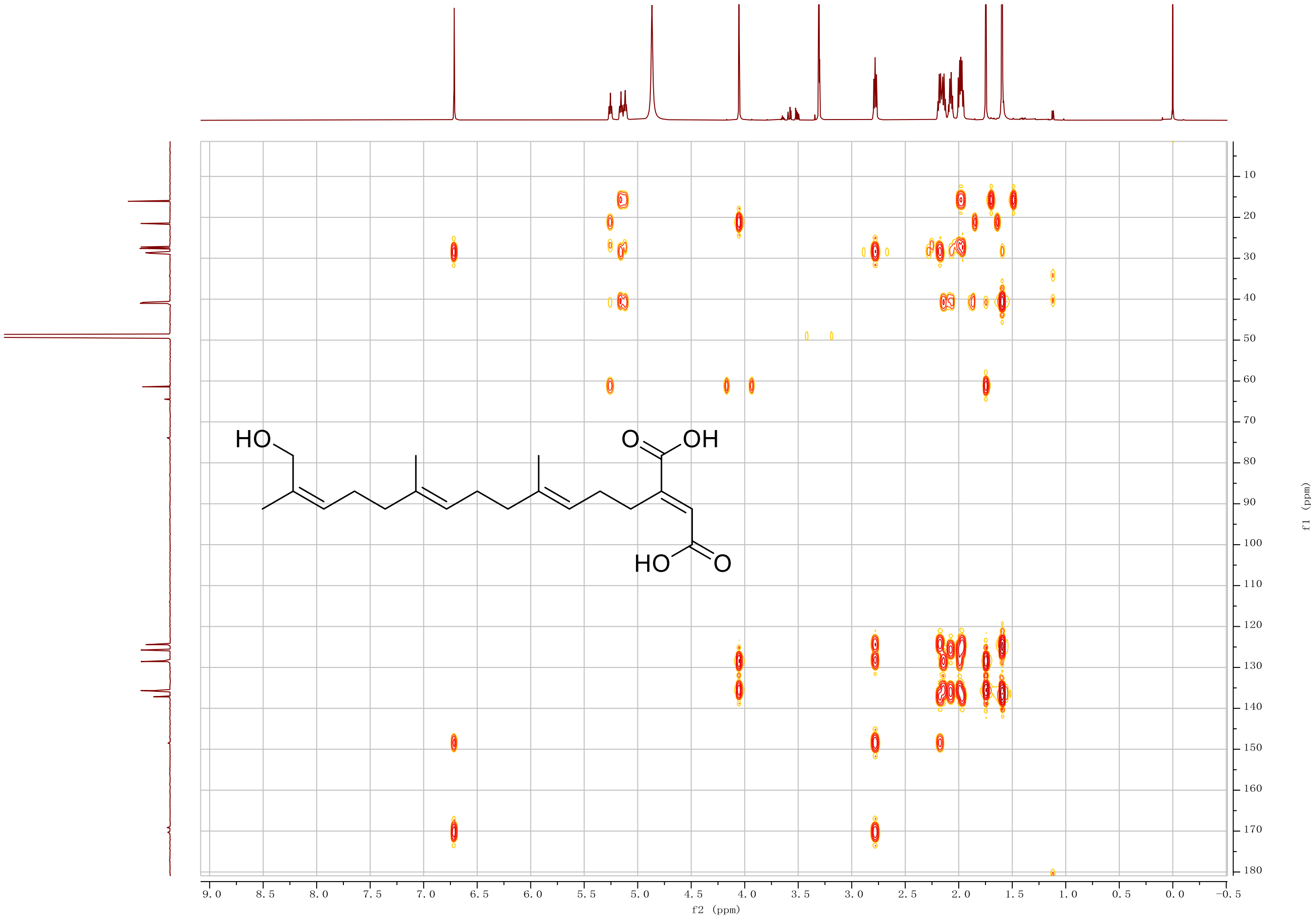

Figure S86. HMBC spectrum of compound **11** in MeOH.

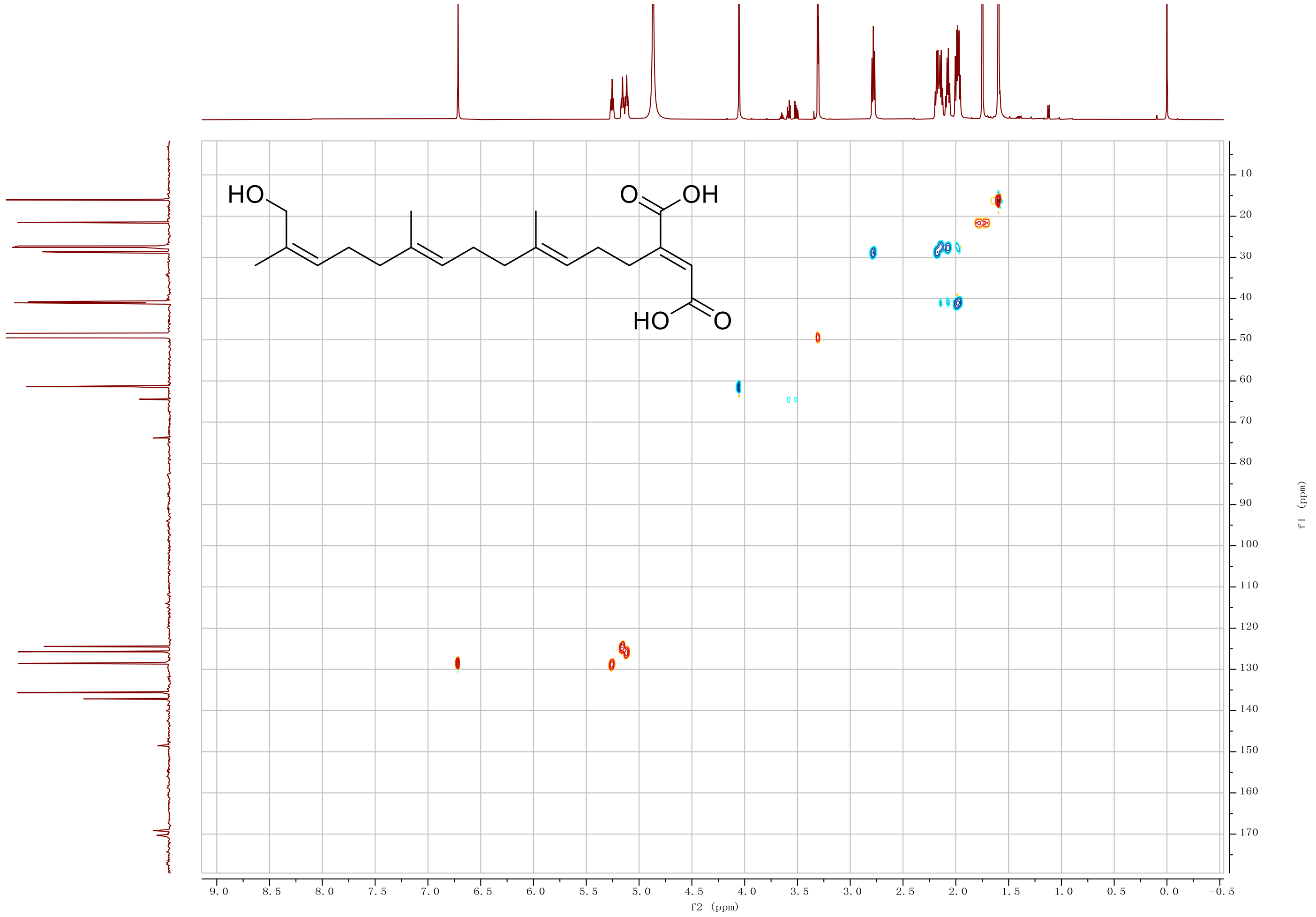

Figure S87. HSQC spectrum of compound **11** in MeOH.

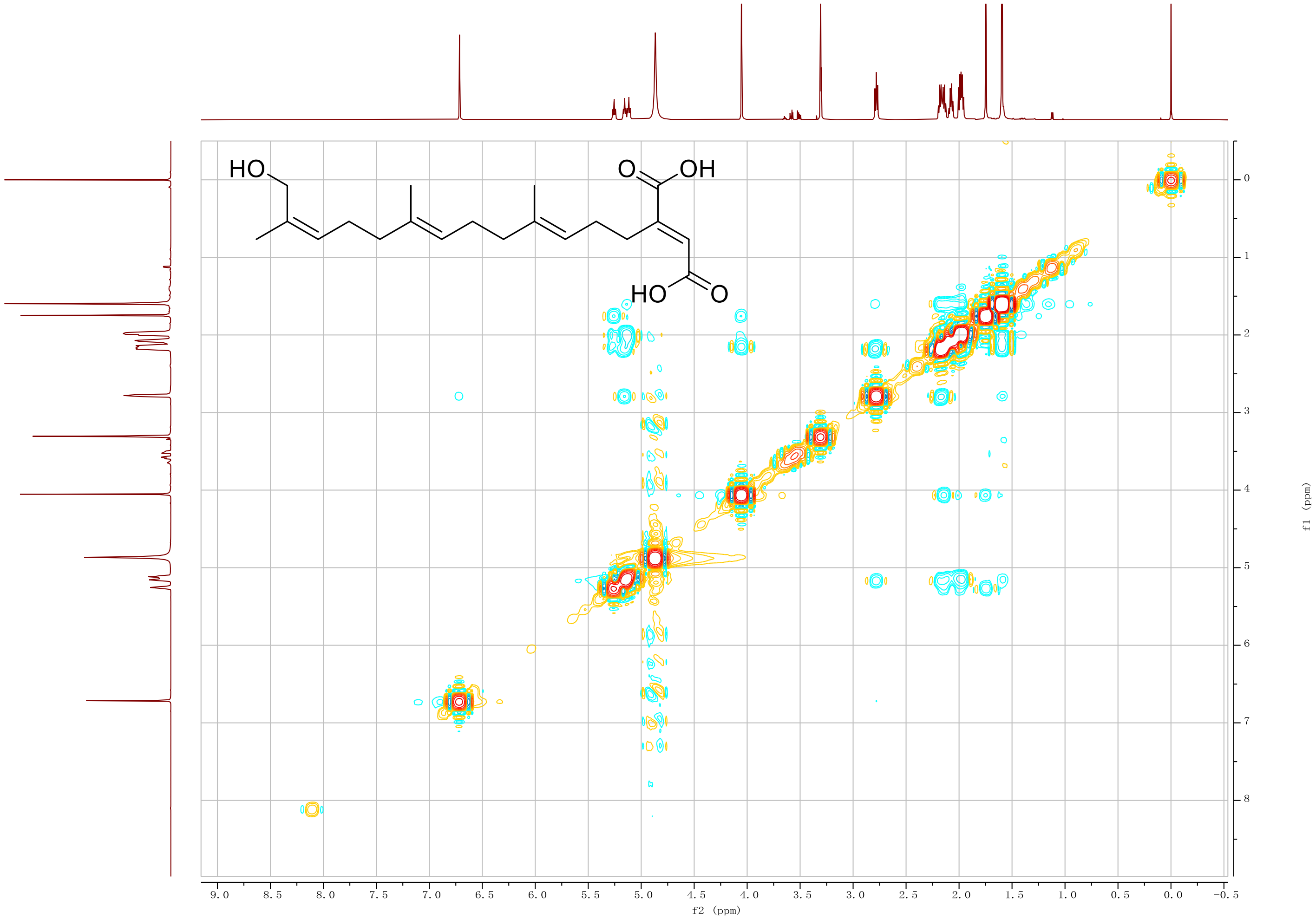

Figure S88. NOESY spectrum of compound **11** in MeOH.

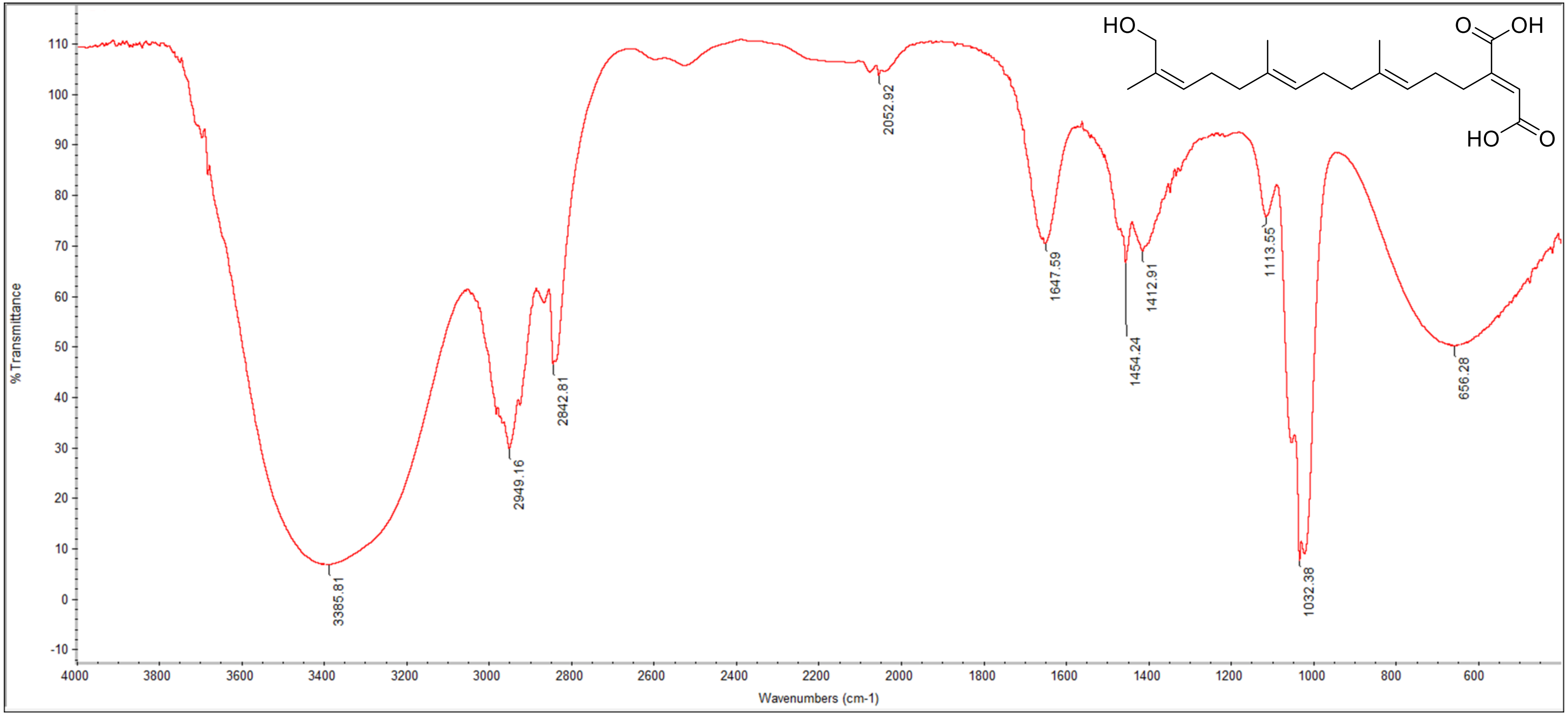

Figure S89. IR spectrum of compound **11** in MeOH.

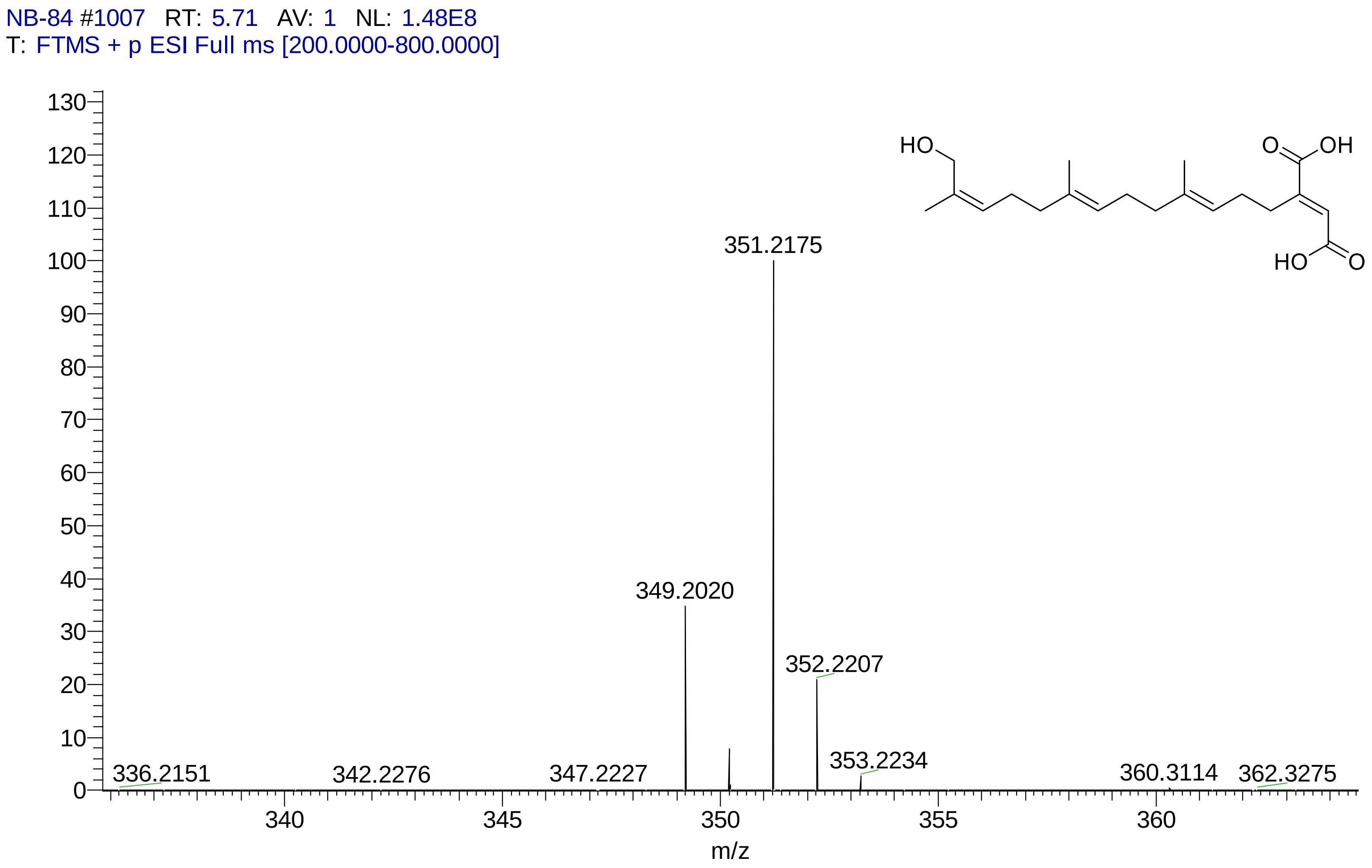

Figure S90. HR-ESI-MS spectrum of compound **11** in MeOH.

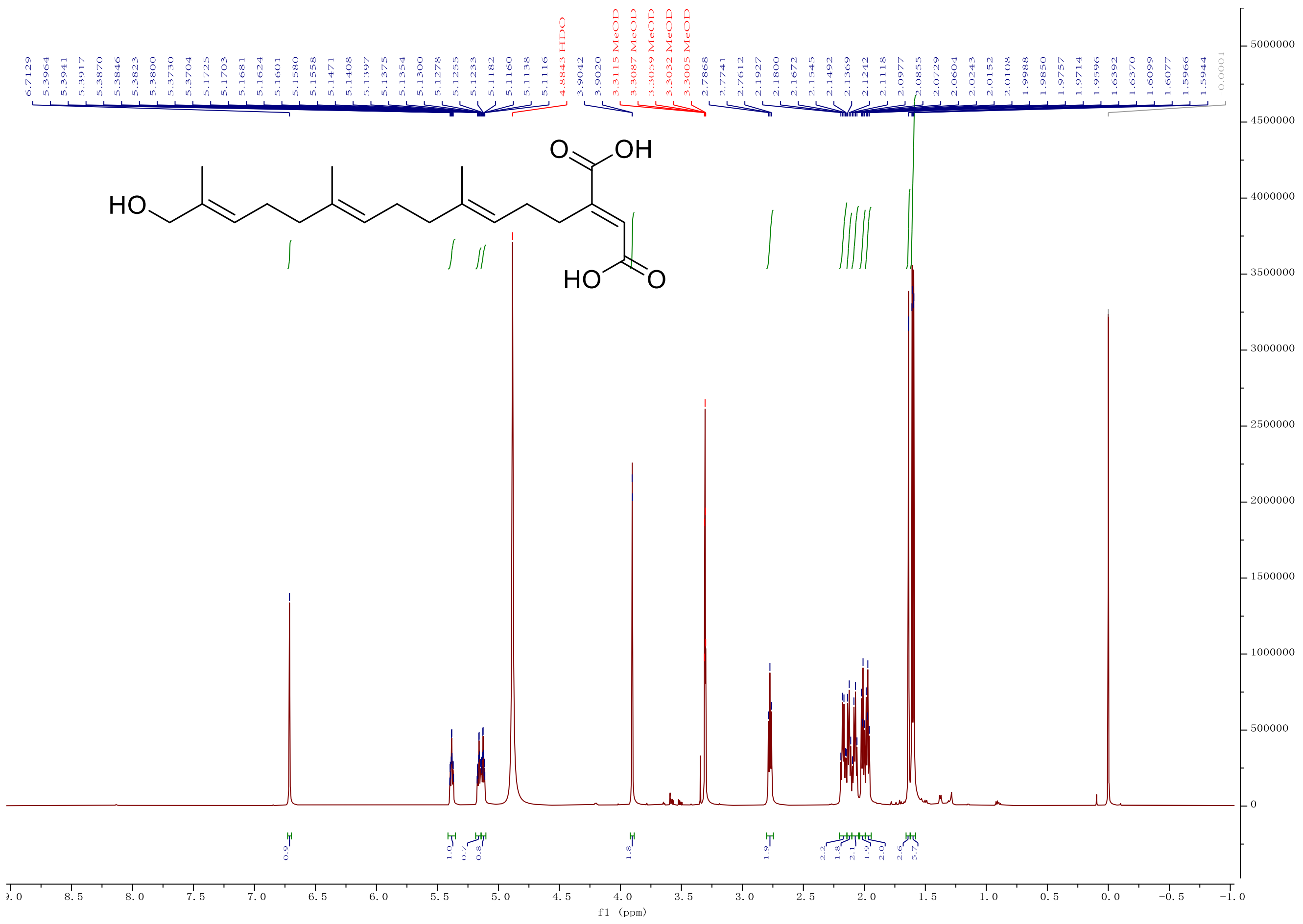

Figure S91. ^{1}H NMR spectrum of compound **12** in MeOH (600 MHz).

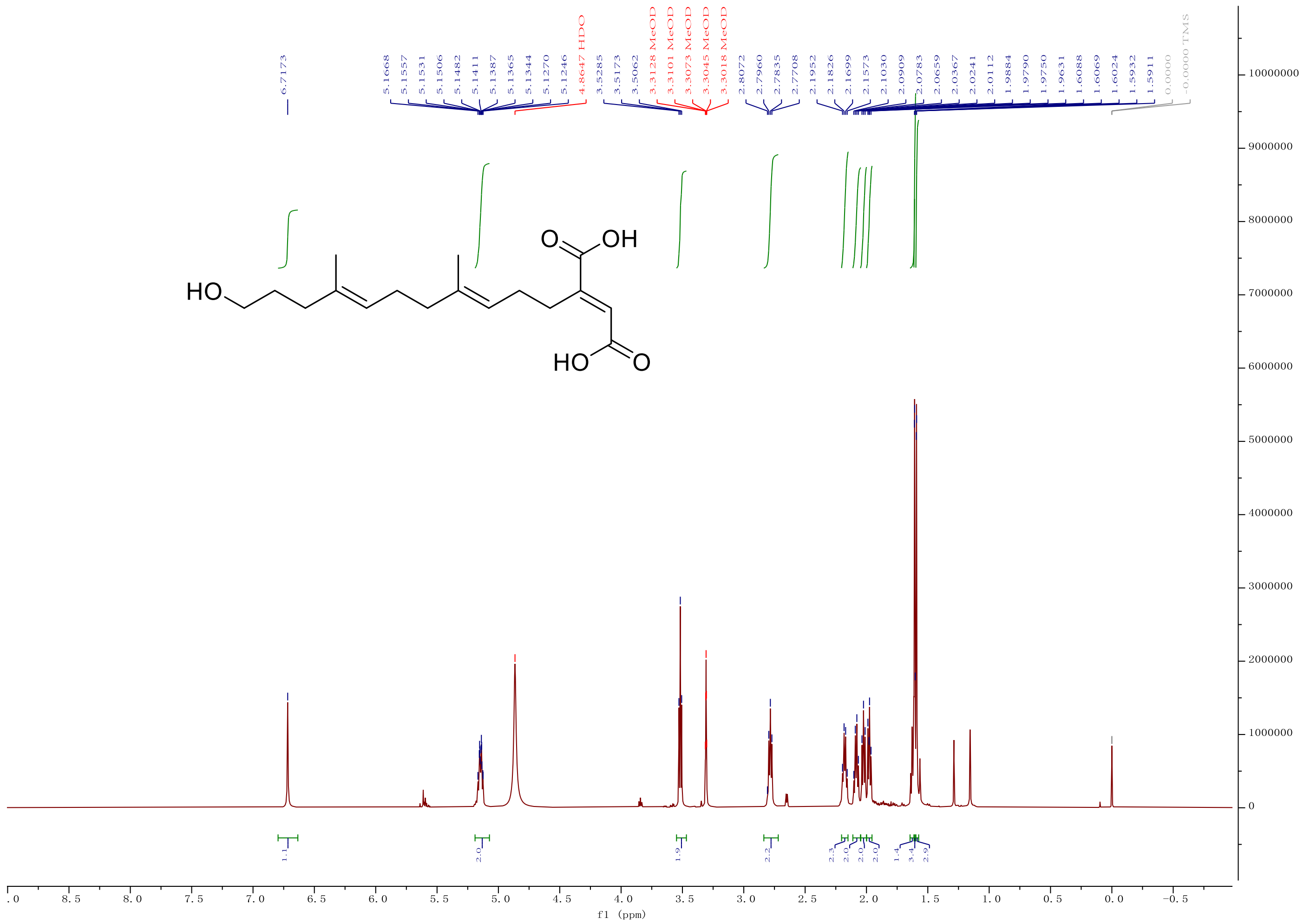

Figure S92. ^{1}H NMR spectrum of compound **13** in MeOH (600 MHz).

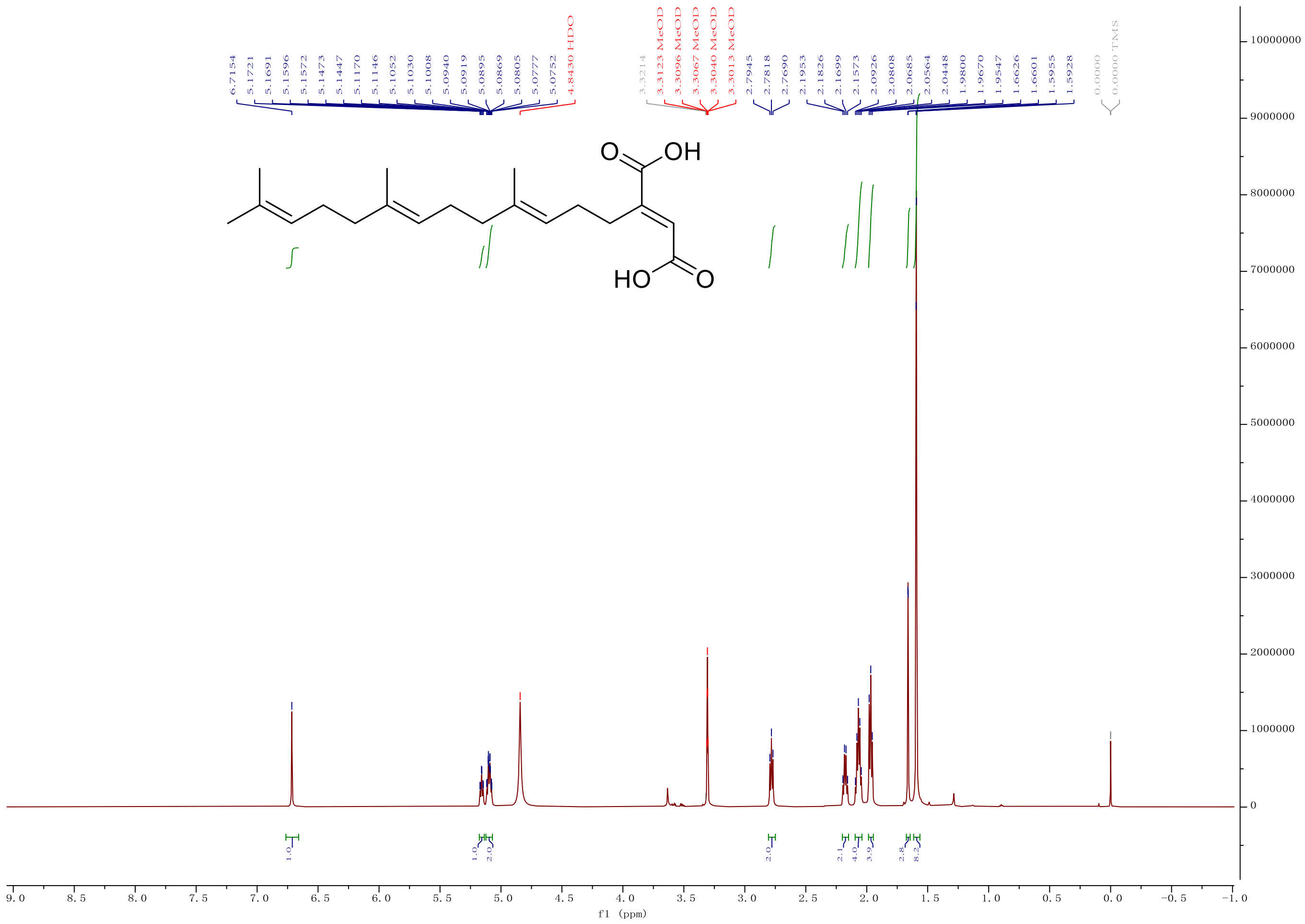

Figure S93. ^{1}H NMR spectrum of compound **14** in MeOH (600 MHz).

ITS sequences of *Schizophyllum commune* NJFU21

AAGAATCAAACAGTTCATCTTGTTCTGATCCTGTGCACCTTATGTAGTCCC
AAAGCCTTCACGGGCGGCGGTTGACTACGTCTACCTCACACCTTAAAGTA
TGTTAACGAATGTAATCATGGTCTTGACAGACCCTAAAAAGTTAATACAA
CTTTCGACAACGGATCTCTTGGCTCTCGCATCGATGAAGAACGCAGCGAA
ATGCGATAAGTAATGTGAATTGCAGAATTCAGTGAATCATCGAATCTTTG
AACGCACCTTGCGCCCTTTGGTATTCCGAGGGGCATGCCTGTTTGAGTGTC
ATTAAATACCATCAACCCTCTTTTGACTTCGGTCTCGAGAGTGGCTTGGAA
GTGGAGGTCTGCTGGAGCCTAACGGAGCCAGCTCCTCTTAAATGTATTAG
CGGATTTCCCTTGCGGGATCGCGTCTCCGATGTGATAATTTCTACGTCGTT
GACCATCTCGGGGCTGACCTAGTCAGTTTCAATAGGAGTCTGCTTCTAACC
GTCTCTTGACTGAGACTAGCGACTTGTGCGCTAACTTTTGACTTGACCTCA
AATCAGGTAGGACTACCCGCTGAACTTAAGCATATCAATAAGGCGGAGGA
ATTA

Table S1. The coordinate for the lowest-energy conformer compound **4-*S*** in VCD calculations.

	X	Y	Z
O	1.70700000	0.97500000	2.03900000
C	2.32000000	1.99200000	1.20900000
C	3.66900000	2.30900000	1.85700000
C	4.03300000	0.99900000	2.51300000
C	2.68400000	0.50100000	2.98100000
C	1.39400000	3.20800000	1.20100000
C	2.48700000	1.47000000	-0.20700000
C	2.32200000	0.19700000	-0.60500000
C	2.43100000	-0.29400000	-2.01900000
C	1.11700000	-0.88900000	-2.49100000
C	0.83500000	-2.18400000	-2.24200000
C	-0.40300000	-2.96000000	-2.60000000
C	-1.32100000	-3.19200000	-1.38900000
C	-2.21000000	-2.00800000	-1.07900000
C	0.20200000	0.07400000	-3.19500000
C	-3.42900000	-1.92600000	-1.92300000
O	-3.69600000	-2.67200000	-2.84900000
O	-4.27000000	-0.93500000	-1.58400000
C	-2.00100000	-1.05700000	-0.14800000
C	-0.83000000	-0.97100000	0.73700000
O	-0.05500000	-1.85400000	1.04700000
O	-0.71600000	0.29600000	1.16900000
H	4.43100000	2.65900000	1.15300000
H	3.55800000	3.07700000	2.63300000
H	4.75400000	1.10400000	3.32800000
H	4.45500000	0.31000000	1.77200000
H	2.63600000	-0.59000000	3.04100000
H	2.42700000	0.91600000	3.96200000
H	1.82600000	4.04400000	0.64100000
H	0.42200000	2.96100000	0.75800000
H	1.18700000	3.54600000	2.22300000
H	2.74700000	2.21700000	-0.95400000
H	2.08400000	-0.56900000	0.13100000
H	2.74700000	0.50600000	-2.70000000
H	3.22500000	-1.05100000	-2.06200000
H	1.57400000	-2.77000000	-1.69100000
H	-0.94800000	-2.50300000	-3.43100000
H	-0.07100000	-3.93800000	-2.97300000
H	-1.95900000	-4.06100000	-1.60100000
H	-0.73500000	-3.50100000	-0.51800000
H	-0.76200000	-0.36300000	-3.46400000
H	-0.00500000	0.93700000	-2.55300000

H	0.67100000	0.43300000	-4.11600000
H	-5.00600000	-1.02600000	-2.22400000
H	-2.69500000	-0.23100000	-0.02200000
H	0.16400000	0.35500000	1.62200000

Table S2. The coordinate for the lowest-energy conformer compound **4-*R*** in VCD calculations.

	X	Y	Z
O	2.21600000	1.24500000	-1.67400000
C	1.48900000	1.34500000	-2.92500000
C	2.20600000	2.41200000	-3.75200000
C	2.79600000	3.31000000	-2.69100000
C	3.17300000	2.31700000	-1.61500000
C	1.54100000	-0.02800000	-3.59400000
C	0.04500000	1.71900000	-2.64400000
C	-0.42400000	2.22000000	-1.48800000
C	-1.87200000	2.45000000	-1.15600000
C	-2.30600000	1.47900000	-0.07300000
C	-2.11900000	1.79100000	1.22700000
C	-2.34100000	0.91400000	2.43000000
C	-1.01700000	0.43700000	3.05500000
C	-0.19200000	-0.43700000	2.13500000
C	-2.84900000	0.17200000	-0.58400000
C	0.80900000	0.28300000	1.30500000
O	1.27200000	1.38200000	1.56000000
O	1.12400000	-0.38100000	0.17900000
C	-0.26200000	-1.77700000	2.02200000
C	-1.21600000	-2.62400000	2.74600000
O	-2.13400000	-2.28500000	3.46600000
O	-0.94900000	-3.91800000	2.50300000
H	1.55400000	2.94700000	-4.44900000
H	3.02300000	1.96800000	-4.33600000
H	3.64600000	3.90000000	-3.04100000
H	2.03300000	4.00200000	-2.31700000
H	3.16800000	2.75700000	-0.61400000
H	4.16300000	1.88700000	-1.80200000
H	1.05500000	-0.02200000	-4.57500000
H	2.57900000	-0.35800000	-3.72400000
H	1.05500000	-0.78800000	-2.97100000
H	-0.66200000	1.51500000	-3.44600000
H	0.27000000	2.44600000	-0.68100000
H	-1.99500000	3.48600000	-0.81800000
H	-2.51100000	2.34300000	-2.04100000
H	-1.68800000	2.76400000	1.46400000
H	-2.88900000	1.50100000	3.17600000
H	-2.98000000	0.05600000	2.20300000
H	-0.43100000	1.30900000	3.37300000
H	-1.23400000	-0.09200000	3.99000000
H	-2.10900000	-0.32400000	-1.22100000
H	-3.11400000	-0.53100000	0.20700000

H	-3.75100000	0.34900000	-1.17800000
H	1.64200000	0.26000000	-0.37200000
H	0.39700000	-2.31700000	1.34900000
H	-1.63100000	-4.40000000	3.01500000
